# The effects of a semen cuscutae flavonoids-based antidepressant treatment on microbiome and metabolome in mice

**DOI:** 10.3389/fmicb.2025.1558833

**Published:** 2025-05-15

**Authors:** Qianfeng Shao, Sheng Zhou, Yue Li, Lin Jin, Xiaowei Fu, Tong Liu, Jing Wang, Shaohui Du, Che Chen

**Affiliations:** ^1^Shenzhen Traditional Chinese Medicine Hospital, Shenzhen, China; ^2^The Fourth Clinical Medical College of Guangzhou University of Chinese Medicine, Guangzhou, China; ^3^Qingdao Ruyi Software Co., Ltd., Medical Data Analysis Center, Qingdao, China; ^4^Centre for Translational Medicine, Shenzhen Bao'an Chinese Medicine Hospital, Guangzhou University of Chinese Medicine, Shenzhen, China; ^5^School of Traditional Chinese Medicine, Beijing University of Chinese Medicine, Beijing, China; ^6^School of Pharmaceutical Sciences, Guangzhou University of Chinese Medicine, Guangzhou, China; ^7^School of Traditional Chinese Medicine, Ningxia Medical University, Yinchuan, China

**Keywords:** SCFs, CUMS, feces, metabolites, microbiota, depression

## Abstract

**Background:**

Depression is a prevalent psychiatric disorder and one of the leading causes of disability worldwide. Previous studies have shown that Semen Cuscutae flavonoids (SCFs) exert antidepressant effects by modulating the microbiota-neuroinflammation axis and ameliorating hippocampal metabolic disturbances. However, the impact of SCFs on gut microbiota and related metabolomics remains largely undefined. Given that the gut microbiota has been proven to play a significant role in the etiology of depression and serves as a promising target for its treatment in humans, this study aims to elucidate the antidepressant effects of SCFs and to investigate how they modulate microbial and metabolic pathways to alleviate depressive symptoms.

**Materials and methods:**

Chronic unpredictable mild stress (CUMS)-induced mice were used as a depression model. The normal mice and CUMS-induced mice were treated with either vehicle or with SCFs. A range of standardized behavioral assays and physiological indicators were employed to evaluate the antidepressant effects of SCFs. Upon the confirmation of the effectiveness of the SCFs treatment, the composition, richness, and diversity of the fecal microbiota were assessed using 16S rRNA gene sequencing. Additionally, fecal metabolic profiling was analyzed using UHPLC-MS/MS-based metabolomics. Multivariate data analysis was subsequently performed to identify differential metabolites and characterize alterations in fecal metabolites. Furthermore, a correlation analysis between differential metabolites and key microbiota was conducted.

**Results:**

SCFs significantly ameliorated depressive behaviors and the dysregulated diversity of fecal microbiota induced by CUMS. SCFs enhanced the gut microbiota structure in the CUMS group by increasing the *Firmicutes*/*Bacteroidota* ratio, significantly elevating the abundance of *Firmicutes, Lactobacillus, Limosilactobacillus*, and *Actinobacteria* while reducing the abundance of *Bacteroidota* and *Bacteroides* in CUMS-treated mice. Fecal metabolomics analyses revealed that SCFs could modulate metabolic pathways, including aldosterone synthesis and secretion, arachidonic acid metabolism, and primary bile acid biosynthesis.

**Conclusions:**

Mice with depression induced by CUMS exhibited disturbances in both their gut microbiota and fecal metabolism. However, SCFs restored the balance of the microbial community and corrected metabolic disturbances in feces, exerting antidepressant effects through a multifaceted mechanism.

## 1 Introduction

Depression is a prevalent psychiatric disorder and one of the leading causes of disability worldwide (WHO, 2017). In addition to its direct impact on work performance and life satisfaction, depression is linked to a range of adverse health outcomes (Choudhary et al., 2022; Harshfield et al., 2020; Matcham et al., 2016). Depression is characterized by persistent sadness, hopelessness, loss of appetite, and anhedonia, which, if left untreated, can escalate to suicidal ideation, presenting a significant challenge to mental health (Ren and Xiao, 2023; Pravesh Kumar, 2024; Varesi et al., 2023). Despite the well-documented symptoms, the pathophysiological mechanisms underlying depression remain largely unclear.

Numerous studies have highlighted a strong association between gut dysbiosis and the onset of depression, emphasizing the crucial role of gut microbiota in regulating central nervous system (CNS) homeostasis, immune function, and metabolic processes, all of which significantly influence overall health (Clemente et al., 2012; Castro-Mejía et al., 2015). Disruptions in the gut microbiota, particularly those induced by stress, profoundly affect the brain-gut axis, influencing cognitive and social behaviors (Mayer et al., 2015; Varesi et al., 2023; Cryan and Dinan, 2012; Kong et al., 2022), and exacerbating depressive symptoms (Jiang et al., 2015). Animal models have shown that stress profoundly disrupts the microbiota-gut-brain axis (Bharwani et al., 2016; Gareau et al., 2007; Golubeva et al., 2015; O'Mahony et al., 2011; Partrick et al., 2018; Bailey and Coe, 1999; Bailey et al., 2004), altering the diversity and composition of gut microbiota and leading to dysbiosis. Fecal transplantation experiments further support this causal relationship, demonstrating that the transplantation of “depression-associated microbiota” into germ-free mice or microbiota-depleted rats induces depression-like behaviors (Kelly et al., 2016a; Zheng et al., 2016). Collectively, these findings underscore the critical role of gut microbiota in CNS function and suggest potential therapeutic targets for diagnosing and treating depression.

The therapeutic response to antidepressants is closely associated with the gut microbiota, and modulation of the gut microbiota is one of the key mechanisms underlying the action of antidepressant drugs (Zhu et al., 2022). However, conventional antidepressants exhibit several limitations, including insufficient efficacy in approximately 30% of patients, long-term effectiveness concerns, a variety of side effects, and the development of drug-resistant depression (Dhir, 2017; Tang et al., 2024). Therefore, alternative approaches to treat or complement antidepressants by enhancing gut microbiota to achieve antidepressant effects have been explored.

The gut microbiota interacts bidirectionally with the brain through the gut-brain axis. For examples, gut microbiota dysbiosis is closely linked to depression, whereas effective antidepressant treatments are often associated with the reduction of the severity or the disappearance of gut microbiota dysbiosis (Maiuolo et al., 2021). In particular, the transplantation of gut microbiota from healthy individuals to depressive patients could lead to beneficial effects (Rao et al., 2021), while the restoration of the homeostasis of the residential gut microbiota of depressive patients could also lead to antidepressive effects. Recently, it has been demonstrated that certain herb remedies, in particular those that are rich in flavonoids, could exert antidepressant effects. Some flavonoids or their metabolic derivatives can have impact on the brain by either directly affecting the brain or indirectly by affecting the gut microbiota via the gut-brain axis. In some cases, the gut microbiota is involved in converting the flavonoids into active metabolites. For an example, the biotransformation of isoflavone glycosides into their bioactive aglycones requires β-glucosidase enzymes that are produced by gut microbiota, particularly *Lactobacillus* and *Bifidobacterium* strains (Stojanov and Kreft, 2020). Semen Cuscutae flavonoids (SCFs), which contain Hyperoside, Isoquercitrin, Astragalin, Quercetin, Kaempferol, and Isorhamnetin as the major components, have been demonstrated of having antidepressant effects in animal models of depression (Ke and Duan, 2013; Shao et al., 2024; Hou et al., 2023). Our previous study demonstrated that SCFs alleviated depressive behaviors and hippocampal metabolic disturbances in CUMS (chronic unpredictable stress) mice, while activating the cAMP-PKA-CREB-BDNF signaling pathway, which is involved in neuroplasticity and mood regulation (Shao et al., 2024). A recent study further demonstrated that SCFs alleviate depression-like behaviors in CUS-induced mice by modulating the gut microbiota-neuroinflammation axis and altering gut microbiota composition (Hou et al., 2023). However, this study only demonstrated microbiome changes between the Semen Cuscutae-150 and Sal groups, excluding comparisons with the control and the CUS groups. Additionally, the mechanisms by which SCFs influence gut microbiota-driven metabolic pathways, particularly those reflected in fecal metabolites, remain unclear.

Fecal metabolites reflect the metabolic activity of both the gut microbiome and the host, making fecal metabolomics a functional indicator of the gut microbiome (Lamichhane et al., 2018; Krautkramer et al., 2021). Growing evidence indicates that gut microbes produce a wide range of chemically diverse metabolites that act as key messengers in regulating stress responses, mood, and behavior (Yamaoka et al., 2022; Leigh et al., 2023; Wiley et al., 2017). Therefore, fecal metabolomics analysis is incorporated into this study to investigate how SCFs influence gut microbiota-driven metabolic pathways.

Based on our previous findings, 28 days of CUMS exposure effectively established a stable depression model in mice, characterized by significant depressive behaviors (Shao et al., 2024). Building on this model, our study utilizes 16S rRNA gene sequencing and non-targeted ultra-high-performance liquid chromatography-tandem mass spectrometry (UHPLC-MS/MS) to analyze gut microbiota composition and fecal metabolites. The objective is to elucidate how SCFs modulate microbial and metabolic pathways to alleviate depressive symptoms. Our findings are anticipated to provide valuable insights into the antidepressant properties of SCFs and to establish a foundation for the development of novel therapeutic agents targeting microbiota-associated pathways in depression.

## 2 Materials and method

### 2.1 Reagents

SCFs preparation was sourced from Chengdu DeSiTe Biological Technology Co., Ltd., China. The SCFs preparation and quantitative analysis were consistent with those used in the previous study (Shao et al., 2024).

### 2.2 Animals and drug administration

**Figure 1:**
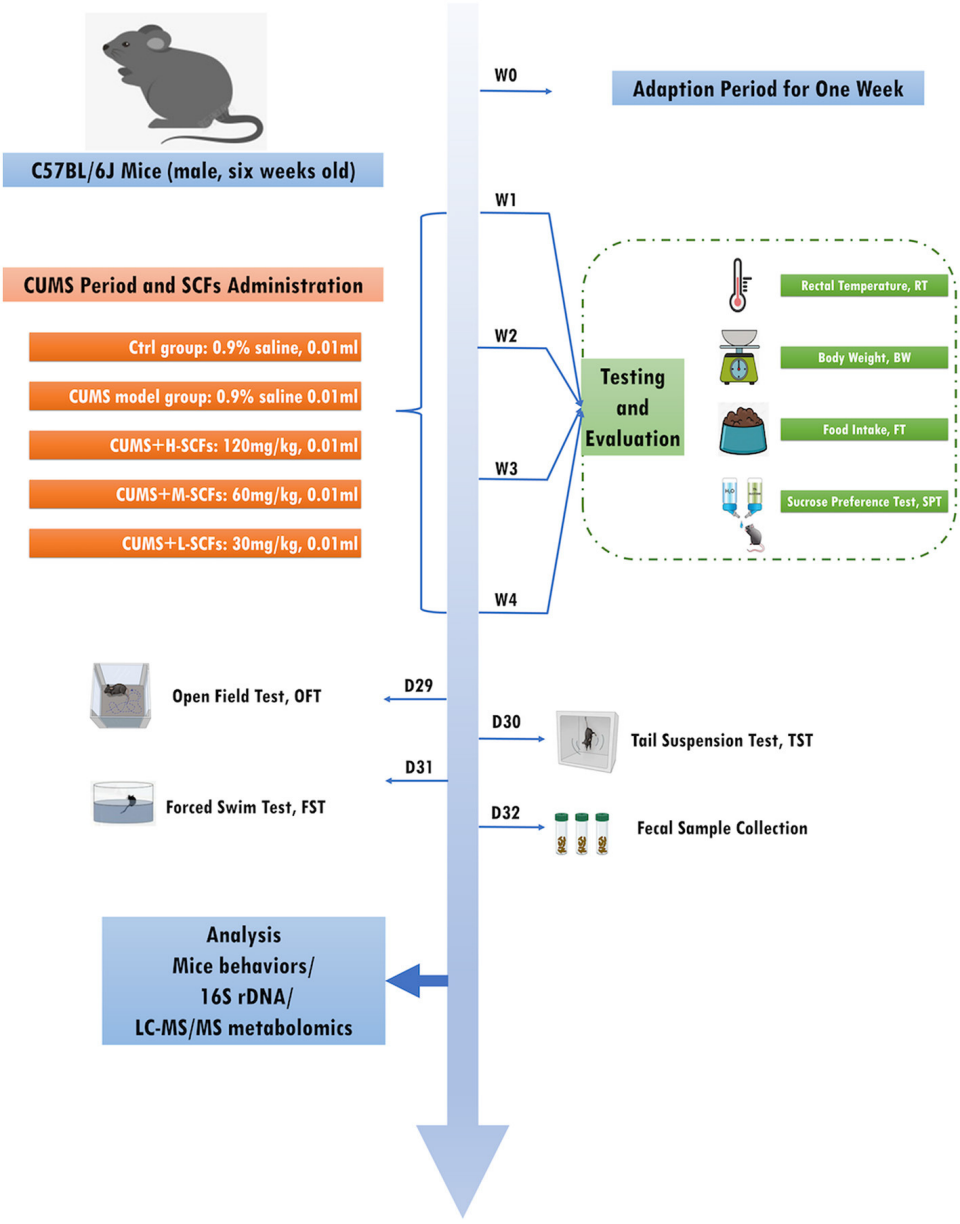
Experimental design flow.

Male C57BL/6J mice (*n* = 50, 6 weeks old, weighing 20–22 g) were obtained from Beijing Vital River Laboratory Animal Technology Co., Ltd (License No.: SCXK [Jing], 2016-0006). The mice were housed in a pathogen-free facility maintained at 22°C ± 1°C with 60% ± 5% relative humidity, fed a standard rodent diet, and under a 12-h light/dark cycle. Following a 7-day acclimation period, baseline measurements of body weight (BW), food intake (FI), rectal temperature (RT), and sucrose preference test (SPT) were recorded. The mice were randomly assigned into five groups: the normal control (CON, *n* = 10), CUMS model (CUMS, *n* = 10), and three SCFs treatment groups-high dose (H-SCFs, 120 mg/kg), medium dose (M-SCFs, 60 mg/kg), and low dose (L-SCFs, 30 mg/kg), each with *n* = 10. All groups, except the CON group, underwent a 28-day chronic unpredictable mild stress (CUMS) protocol, with SCFs administered concurrently during CUMS exposure, excluding the CON and CUMS groups. BW, FI, RT, and SPT were measured on a weekly basis. Behavioral tests, including the open field test (OFT), tail suspension test (TST), and forced swim test (FST), were performed on days 29, 30, and 31, following 28 days of CUMS treatment. Fecal samples were collected on the final experimental day. All procedures were conducted in accordance with the National Research Council's Guide for the Care and Use of Laboratory Animals (Figure 1).

### 2.3 CUMS modeling procedure

The CUMS modeling protocol followed the same methodology as outlined in the previous study (Shao et al., 2024). The CON group mice were group-housed under standard conditions, while the other four groups were individually housed and exposed to two different stressors daily for 28 days, with no stressor repeated consecutively to prevent predictability (Supplementary Table 1).

### 2.4 Measurements of behavioral test

To comprehensively assess the condition of the depression model mice and the antidepressant effects of SCFs, we monitored physiological parameters, including BW, FI, RT, and behavioral indicators (SPT, OFT, TST, and FST).

#### 2.4.1 Physiological parameters

Body weight was measured in a quiet, dry environment to minimize stress. The mouse was placed in a weighing pan to ensure it remained still, and the weight was recorded weekly at 7 a.m. Weight changes were tracked in a data table for analysis. Rectal temperature was measured by gently restraining the mouse, inserting the thermometer 1–2 cm, and recording the temperature; the thermometer was disinfected after use. Food intake was measured by weighing each food container at 8 a.m. the day before testing, providing equal food amounts for 24 h, and reweighing the containers. Calculate food intake as Food intake = Initial weight – Remaining weight for each mouse.

#### 2.4.2 Sucrose preference test

The SPT is used for assessing anhedonia and evaluating antidepressant efficacy (Willner et al., 1987). Mice were housed individually and acclimatized to two bottles of 1% sucrose solution on the first day. On the following day, one bottle was replaced with water, and the positions of the bottles were swapped every 4 h to prevent habituation. After a 24-h fasting period, the mice were given a pre-weighed 1% sucrose solution and water, and liquid intake was measured after 2 h. The sucrose preference rate was calculated as: sucrose intake/(sucrose + water intake) × 100%. Sucrose preference tests were conducted weekly throughout the CUMS procedure.

#### 2.4.3 Open field test

The OFT in this study is used to assess the anxiety of CUMS-induced mice (Walsh and Cummins, 1976). The OFT was conducted in a 50 cm × 50 cm × 40 cm open field apparatus (Model: ZS-KC, No. 1056025), divided into nine squares, with the center square designated as the central area and the others as the peripheral areas. Each mouse was placed in the center and allowed to explore for 5 min, while video tracking software (Tracking Master V3.1.62., Beijing Zhongshi Dichuang) recorded the total distance traveled and the time spent in the center. The apparatus was cleaned with 75% alcohol between tests to prevent contamination from previous trials.

#### 2.4.4 Tail suspension test

The TST aims to assess depression-like behaviors (Cryan et al., 2005). Each mouse was suspended 40 cm above the floor for 6 min using adhesive tape on the edge of a rod in a soundproof, visually isolated chamber. Immobility time, defined as the absence of escape attempts, was recorded using video tracking software (Tracking Master V3.1.62.). After a 1-min habituation period, immobility time was measured during the final 5 min.

#### 2.4.5 Forced swim test

The FST is used in depression research to assess depression-like behaviors by observing the immobility of rodents when placed in an inescapable water environment (Porsolt et al., 1977). Each mouse was placed in a transparent glass cylinder (45 cm high, 20 cm diameter) filled with water to a depth of 25 cm at 24°C ± 1°C. After a 1-min adaptation period, immobility time was recorded over the next 5 min using a video system (Tracking Master V3.1.62). Immobility was defined as the absence of movement with passive floating.

### 2.5 16s rRNA gene sequencing analysis of fecal microbiota

Twenty-one fecal samples were processed using the QIAamp DNA Stool Mini Kit (Qiagen, USA). Total genomic DNA was extracted using the CTAB method. PCR amplification targeted 16S rRNA genes using specific primers (16SV4:515F-806R). Sequencing was performed on an Illumina MiSeq platform, and the data were analyzed using QIIME, DADA2, and Mothur. Taxonomic classification was performed, and OTU phylogenetic relationships were analyzed using MUSCLE software. Relative taxon abundance was determined using the SVG function and the R package microeco, and phylogenetic relationships of OTUs were visualized using heatmap and VennDiagram functions in R.

### 2.6 Untargeted metabolomics analyses

#### 2.6.1 Metabolites extraction

For each fecal sample, 100 mg of frozen tissue was ground with liquid nitrogen, and the homogenate was resuspended in prechilled 80% methanol with vortexing. The samples were incubated on ice for 5 min and then centrifuged at 15,000 g at 4°C for 20 min. The supernatant was diluted with water to a final concentration of 53% methanol, followed by centrifugation at 15,000 g at 4°C for 20 min. The supernatant was subsequently used for LC-MS/MS analysis.

#### 2.6.2 UHPLC-MS/MS analyses

UHPLC-MS/MS analyses were performed using a Vanquish UHPLC system (ThermoFisher, Germany) coupled with an Orbitrap Q ExactiveTM HF mass spectrometer or Orbitrap Q ExactiveTMHF-X mass spectrometer (Thermo Fisher, Germany) at Novogene Co., Ltd. (Beijing, China) as described. 18 samples were injected onto a Hypersil Goldcolumn (100 × 2.1 mm, 1.9 μm) using a 12-min linear gradient at a 0.2 mL/min flow rate. The eluents for the positive and negative polarity modes were eluent A (0.1% formic acid in water) and eluent B (methanol). The solvent gradient was set as follows: 2% B, 1.5 min; 2%−85% B, 3 min; 85%−100% B, 10 min; 100%−2% B, 10.1 min; 2% B, 12 min. Q ExactiveTM HF mass spectrometer was operated in both positive and negative polarity modes with a spray voltage of 3.5 kV, a capillary temperature of 320°C, a sheath gas flow rate of 35 psi, and an aux gas flow rate of 10 L/min, S-lens RF level of 60, Aux gas heater temperature of 350°C.

#### 2.6.3 Metabolite identification and quantification

The raw UHPLC-MS/MS data were processed using Compound Discoverer 3.3 (CD3.3, ThermoFisher) for peak alignment, peak picking, and quantification as described. Key parameters included peak area correction with the first quality control (QC), a mass tolerance of 5 ppm, a signal intensity tolerance of 20%, and minimum intensity settings. Peak intensities were normalized to total spectral intensity, and molecular formulas were predicted based on additive ions, molecular ion peaks, and fragment ions. Peaks were matched against mzCloud, mzVault, and MassList databases for accurate identification and quantification. Statistical analyses were conducted using R (version 3.4.3), Python (version 2.7.6), and CentOS (release 6.6). Data that were not normally distributed were standardized by relative peak areas, with compounds having CVs over 20% in QC samples removed.

### 2.7 Data and statistical analysis

#### 2.7.1 16Sr DNA gene sequencing analyses

Microbial alpha diversity indices were calculated using QIIME software (version 1.7.0). Beta diversity was assessed using the Weighted UniFrac distance and visualized through Principal Coordinate Analysis (PCoA) in R software. The Adonis test was used to evaluate grouping significance. LEfSe software (version 1.0) was used to identify biomarkers, and the MetaStat method analyzed species abundance differences.

#### 2.7.2 Untargeted metabolomic analyses

Metabolite identification and annotation were conducted using the KEGG, HMDB, and LIPIDMaps databases. Principal component analysis (PCA) and orthogonal projections to latent structures discriminant analysis (OPLS-DA) were performed using the metaX metabolomics data processing software to determine the variable importance in projection (VIP) of metabolites. S-plot and permutation tests using SIMCA-P 14.1 software. Univariate analysis (*t*-test) was employed to calculate the statistical significance (*P*-value) of metabolites and to assess the fold change (FC) between groups. Spearman's correlation test using the R (version 2.15.3) psych package for calculation and illustration examined the correlation between gut microbiota, fecal metabolites, and depression-related indicators at the taxonomic level.

## 3 Results

### 3.1 SCFs treatment ameliorates depressive-like behaviors in CUMS mice

**Figure 2:**
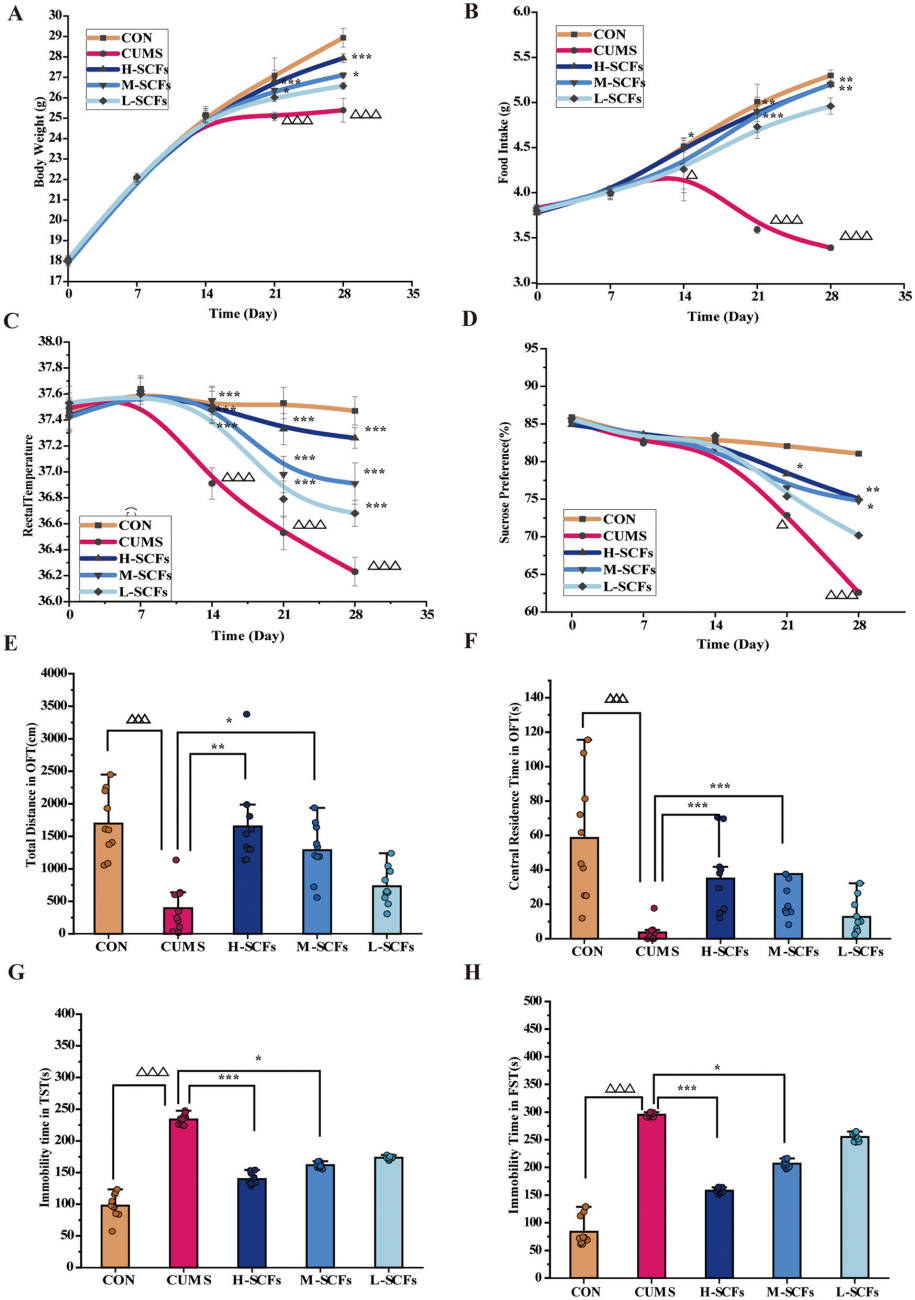
SCFs relieved depression-like behaviors and physiological indicators in mice exposed to CUMS. **(A)** Weekly body weight changes of the mice during 28 days. **(B)** Weekly food intake changes during 28 days. **(C)** Weekly rectal temperature changes during 28 days. **(D)** Weekly the sucrose preference rate changes during 28 days. **(E)** Total distance in OFT on the day 29. **(F)** The time spent in the central area in the OFT on the day 29. **(G)** The immobility time in the TST on the day 30. **(H)** The immobility time in the FST on the day 31. Compared to the CON group, ^Δ^*P* < 0.05, ^ΔΔΔ^*P* < 0.001; compared to the CUMS group, *P < 0.05, **P < 0.01, ***P < 0.001.

The results demonstrated the ameliorative effects of SCFs treatment on CUMS-induced depressive-like behaviors in mice, as assessed by body weight, food intake, thermoregulation, and various behavioral tests. Mice exposed to CUMS exhibited a trend of reduced weight gain from day 14, with a significant decrease in weight observed between days 21 and 28 (*P* < 0.001; Figure 2A), which was closely associated with decreased food intake (Figure 2B). From day 14 onwards, food intake in the CUMS group was significantly lower than that in the CON group (*P* < 0.05), with further declines observed on days 21 and 28 (*P* < 0.001). These results suggest that the CUMS-induced depression model affects body weight through reduced appetite and food intake.

In contrast, starting from day 14, the H-SCFs group showed a significant improvement in food intake (*P* < 0.05 on day 14; *P* < 0.01 on days 21 and 28) and a significant increase in body weight on days 21 and 28 (*P* < 0.001), suggesting that high-dose SCFs treatment restored body weight in CUMS mice by enhancing appetite and food intake. Similarly, the M-SCFs group showed significant improvements in food intake and body weight starting from day 21 (*P* < 0.001 for both food intake and body weight on day 21; *P* < 0.001 on day 28), suggesting that medium-dose SCFs treatment also mitigated the CUMS-induced suppression of weight and appetite. Although the L-SCFs group displayed a trend toward increased food intake and body weight, these changes were not statistically significant, suggesting a weaker effect of the low-dose SCFs treatment in the CUMS model. Overall, these findings suggest that medium and high doses of SCFs treatment provide substantial protection against CUMS-induced reductions in body weight and appetite, likely through appetite stimulation and subsequent mitigation of weight loss, with the high dose demonstrating the most pronounced effects. Thus, SCFs treatment shows potential as a preventive antidepressant intervention during the early stages of stress, particularly at higher doses.

Thermoregulation was another key physiological dysfunction observed in CUMS mice (Figure 2C). Starting from day 14, rectal temperatures in the CUMS group were significantly lower than those in the CON group (*P* < 0.001) and continued to decline through days 21 and 28 (*P* < 0.001), highlighting the systemic impact of CUMS. SCFs treatment at high, medium, and low doses significantly elevated rectal temperatures starting from day 14 (*P* < 0.001), restoring normal body temperature levels. These findings suggest that SCFs treatment not only improved food intake and body weight in CUMS mice but also alleviated physiological stress responses induced by CUMS through thermoregulation.

Additionally, the SPT revealed that the sucrose preference in the CUMS group gradually decreased from day 7, with a significant reduction from day 21 to day 28 (*P* < 0.05, *P* < 0.001, respectively), reflecting clear depressive-like behaviors (Figure 2D). In contrast, the SCFs-treated groups exhibited dose-dependent improvement. Compared to the CUMS group, the sucrose preference in the H-SCFs group significantly increased from day 21 (*P* < 0.05) and continued to improve by day 28 (*P* < 0.01). The M-SCFs group showed significant improvement by day 28 (*P* < 0.05), whereas the L-SCFs group showed an upward trend without reaching statistical significance. This indicated that SCFs could alleviate CUMS-induced depressive-like behaviors. The recovery in sucrose preference was also consistent with increased food intake and body weight, further demonstrating the potential of SCFs to combat depressive symptoms in multiple dimensions.

After 28 days of CUMS modeling, compared to the CON group, the total distance traveled and time spent in the central area in the OFT were significantly reduced in the CUMS group (*P* < 0.001, *P* < 0.001, respectively), reflecting avoidance of the new environment and heightened anxiety in the CUMS mice (Figures 2E, F). The immobility time in both the TST and FST was significantly increased (*P* < 0.001, *P* < 0.001, respectively), indicating that 28 days of CUMS modeling induced feelings of helplessness and despair in the mice. In the high and medium dose SCFs groups, the total distance traveled (*P* < 0.01, *P* < 0.05, respectively) and time spent in the central area (*P* < 0.001, *P* < 0.001, respectively) were significantly increased in the OFT, with the H-SCFs group nearly reaching the levels of the CON group. Both high and medium doses of SCFs significantly improved the immobility time in the TST (Figure 2G) and FST (Figure 2H; *P* < 0.001, *P* < 0.05; *P* < 0.001, *P* < 0.05, respectively). These findings suggested that SCFs may improve the emotional state of mice by promoting exploratory behavior and reducing helplessness, with particularly pronounced effects at higher doses, showing strong antidepressant effects. Although the low-dose SCFs group showed a trend of improvement, it did not reach statistical significance. Therefore, SCFs significantly alleviated CUMS-induced depressive-like behaviors, with high and medium doses showing especially strong effects.

Overall, SCFs demonstrated significant antidepressant effects in multiple behavioral tests at high and medium doses, with the high-dose group showing more pronounced improvements in both physiological indicators and depressive behaviors.

### 3.2 Regulatory effects of SCFs on the fecal microbiota in CUMS- induced mice

Due to the pronounced therapeutic effects of the high-dose SCFs on depressive behaviors, only the H-SCFs group was selected for 16S rRNA gene sequencing analysis along with the CON and CUMS groups to assess gut microbiota changes after CUMS modeling and SCFs administration.

#### 3.2.1 SCFs significantly regulated the abnormal diversity of gut microbiota induced by CUMS

**Figure 3:**
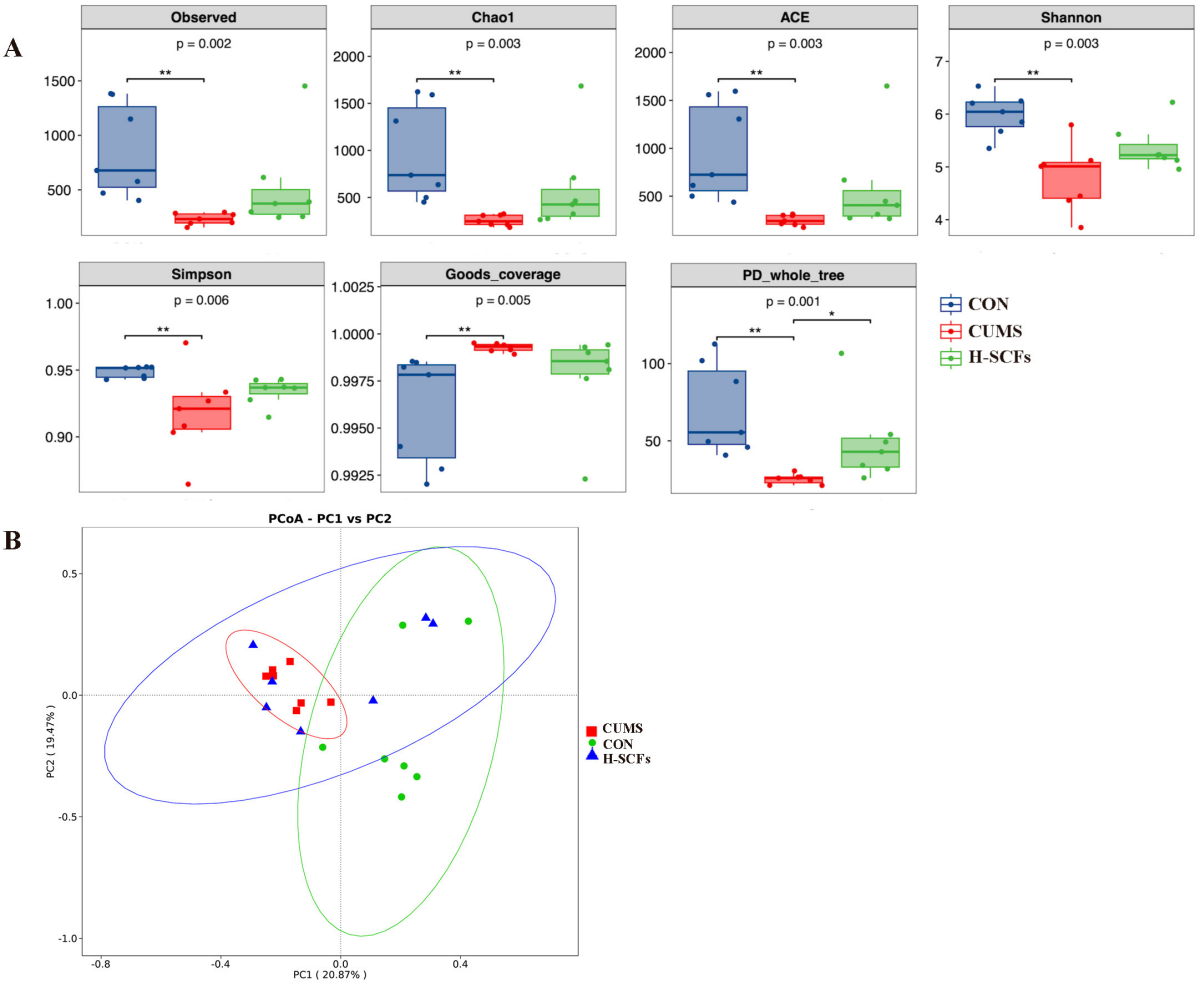
**(A)** Alpha-phylogenetic diversity analysis showed that depressive-like mice induced by CUMS had lower microbial diversity and richness in six indexes relative to the CON (*P* < 0.01, respectively); these indexes had increased when administrated with H-SCFs. **(B)** Beta diversity analysis. PCoA revealed that the fecal microbiome composition in CUMS mice was markedly distinct from that of CON, and the fecal microbial composition was restored after being treated with H-SCFs. *P < 0.05, **P < 0.01.

In the alpha diversity analysis, the fecal microbiota community revealed that findings on diversity measurements (Shannon, PD_whole_tree, and Simpson indices; *P* < 0.01, respectively), as well as richness assessments (Chao1, ACE, and observed indices; *P* < 0.01, respectively) in CUMS group, were significantly lower than those in the CON group, while SCFs increased the microbial diversity and richness indices induced by CUMS-exposure, especially significantly increased PD_whole_tree indices (*P* < 0.05). The Good's coverage estimate showed that the coverage of all samples exceeded 95%, indicating that the sequencing depth was sufficient (Figure 3A). Beta diversity analysis focuses on comparing the diversity of microbiota among different samples. PCoA analysis based on the weighted UniFrac distance matrix (PCo1 = 18.8%, PCo2 = 20.7%) showed significant separation between the CON and CUMS group, while the SCFs group overlaps with both but seems closer to the CON group, indicating potential restoration or normalization of the microbial composition in the SCFs-treated group (Figure 3B). The results demonstrated that CUMS led to significant disruptions in the fecal microbial community, while SCFs treatment effectively mitigated the dysbiosis induced by CUMS in mice.

#### 3.2.2 SCFs significantly regulated the abnormal abundances of potential microbial biomarkers altered by CUMS

**Figure 4:**
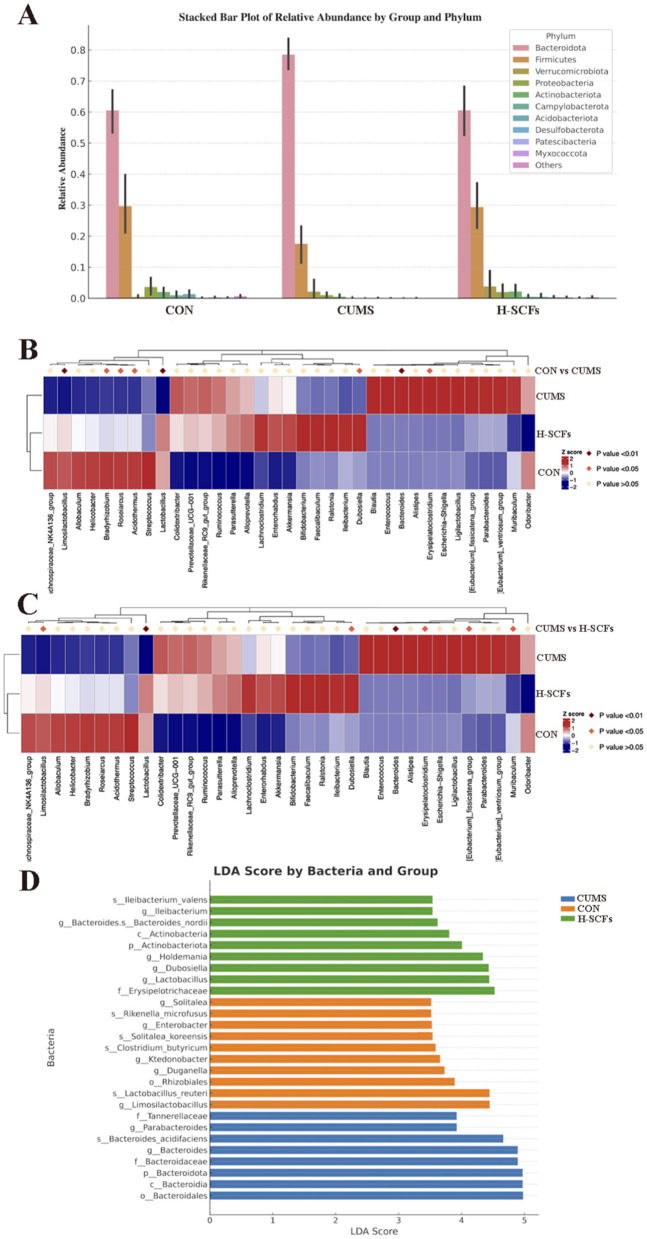
**(A)** The relative abundance of the top 10 gut microbiota at the phylum level. **(B)** The metastat analysis on the top 10 differential species at the phylum between the CON and CUMS groups. **(C)** The metastat analysis on the top 10 differential species at the phylum between the CUMS and H-SCFs groups. **(D)** Column chart of linear discriminant analysis (LDA). Linear discriminant analysis effect size (LEfSe) analysis was conducted with a threshold of LDA > 3.5. The results were visualized using a histogram and showed 27 responsible for discriminating in CON, CUMS, and H-SCFs groups.

To further investigate the specific impacts of the CUMS model and SCFs intervention on the fecal microbiota of mice, we analyzed changes in the composition and abundance of microbial taxa across the groups. We focused on the top 10 phyla based on their abundance to calculate relative abundance, which was then visualized using stacked bar charts (Figure 4A). The relative abundance of gut microbiota at the phylum level was as follows: *Bacteroidetes* (*Bacteroidota*), *Firmicutes, Verrucomicrobiota*, and *Proteobacteria* were the most abundant phylum among all groups, accounting for more than 90% of the total bacterial community (Figure 4A). Additionally, in the CON group, *Bacteroidota* and *Firmicutes* levels were 60.53% and 29.68%, with an F/B ratio of 53.28%. In the CUMS group, these levels shifted to 78.51% and 17.55%, and the F/B ratio decreased to 23.25%. After SCFs treatment, the levels returned to 60.54% and 29.40%, with an F/B ratio of 51.83%, indicating significant restoration of gut microbiota in CUMS-treated mice (Supplementary Table 2). We further conducted a metastat analysis on the top 10 differential species at the phylum level (Figures 4B, C). Compared with the CON group, the relative abundance of *Bacteroidota* was significantly increased in the CUMS group (*P* < 0.01), while the relative abundances of *Firmicutes* (*P* < 0.05), *Patescibacteria* (*P* < 0.01), *Myxococcota* (*P* < 0.05), and *Acldobacteriota* (*P* < 0.05) were significantly decreased. After H-SCFs treatment, the relative abundance of *Bacteroidota* was significantly decreased (*P* < 0.01), while the relative abundances of *Patescibacteria* (*P* < 0.01) and *Firmicutes* (*P* < 0.01) were significantly increased.

**Figure 5:**
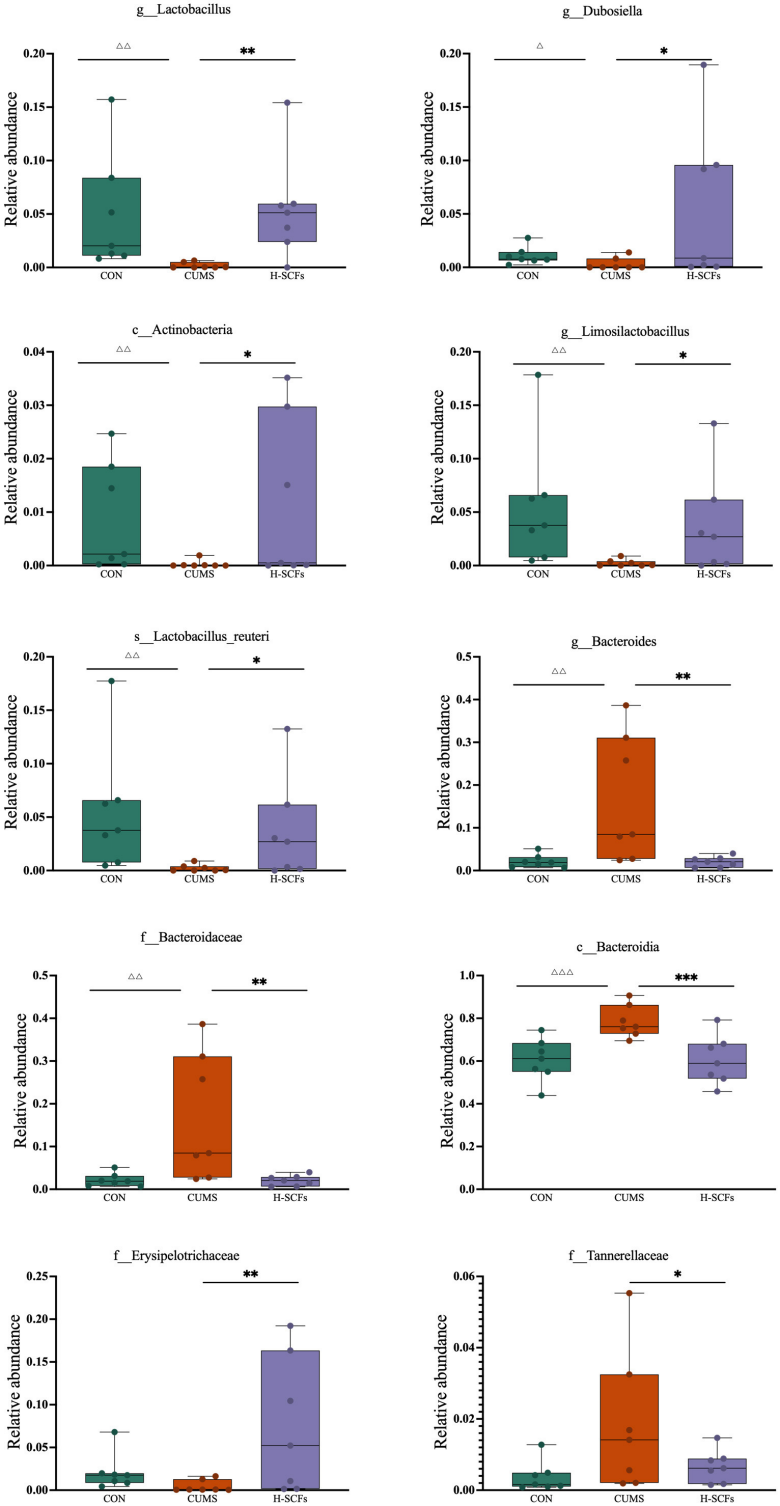
The metastat analysis of the dominant bacteria among the CON, CUMS, and H-SCFs groups. Compared to the CON group, ^Δ^*P* < 0.05, ^ΔΔ^*P* < 0.01, ^ΔΔΔ^*P* < 0.001; compared to the CUMS group, *P < 0.05, **P < 0.01, ***P < 0.001.

LEfSe was used to identify the characteristics that best explain the differences between groups and assess the extent to which these characteristics influence those differences. We performed LDA scoring to evaluate the impact of individual bacterial abundances and identified significant differences among the three groups (LDA scores > 3.5, *P* < 0.05; Figure 4D). A total of 27 bacterial branches showed statistically significant differences. The CON group presented 10 distinct taxa, including g_*Limosilactobacillus*, s_*Lactobacillus reuteri*, o_*Rhizobiales*, g_*Duganella*, g_*Ktedonobacter*, s_*Clostridium butyricum*, s_*Solitalea koreensis*, g_*Enterobacter*, s_*Rikenella microfusus*, and g_*Solitalea*. In the CUMS group, 8 bacterial taxa were notably different, such as *o_Bacteroidales, c_Bacteroidia, p_Bacteroidota, g_Bacteroides, f_Bacteroidaceae, s_Bacteroides acidifaciens, g_Parabacteroides*, and *f_Tannerellaceae*. The SCFs group presented 9 different taxonomic levels, including *f_Erysipelotrichaceae, g_Lactobacillus, g_Dubosiella, g_Holdemania, p_Actinobacteriota, c_Actinobacteria, s_Bacteroides nordii, g_Ileibacterium*, and *s_Ileibacterium valens*. Following this, we conducted a metastat analysis to further explore the relative abundance changes of dominant bacteria between the groups (Figure 5). The results showed that, compared to the CON group, the relative abundance of *Lactobacillus* (*P* < 0.01), *Dubosiella* (*P* < 0.05), *Actinobacteria* (*P* < 0.01), *Limosilactobacillus* (*P* < 0.01), and *Lactobacillus_reuteri* (*P* < 0.01) decreased after CUMS intervention, while the relative abundance of *Bacteroides* (*P* < 0.01), *Bacteroidaceae* (*P* < 0.01), *Bacteroidia* (*P* < 0.01) increased. The SCFs treatment increased the relative abundance of *Lactobacillus* (*P* < 0.01), (*P* < 0.01), *Actinobacteria* (*P* < 0.01), *Limosilactobacillus* (*P* < 0.05), and *Lactobacillus_reuteri* (*P* < 0.05), while decreasing the relative abundance of *Bacteroides* (*P* < 0.01), *Bacteroidaceae* (*P* < 0.01), *Tannerellaceae* (*P* < 0.05), and *Bacteroidia* (*P* < 0.01).

### 3.3 Impact of SCFs intervention on fecal metabolic profiles

In this study, we first verified the significant effects of high-dose SCFs in alleviating multiple depression-like symptoms and modulating gut microbiota composition in CUMS mice. Subsequently, we conducted a comprehensive UHPLC-MS/MS-based metabolomic analysis of fecal samples from three groups of mice: the CON, CUMS, and H-SCFs groups. By studying the metabolomics and microbiota of these fecal samples, we aimed to gain deeper insights into how gut microbes influence overall metabolic processes and their relationship with depression. This approach not only revealed SCFs-induced metabolic changes in the CUMS mice but also complemented our previous findings on hippocampal metabolism, offering a more comprehensive perspective for understanding the multifaceted mechanisms of depression and the potential role of SCFs in its treatment.

#### 3.3.1 PCA-score

**Figure 6:**
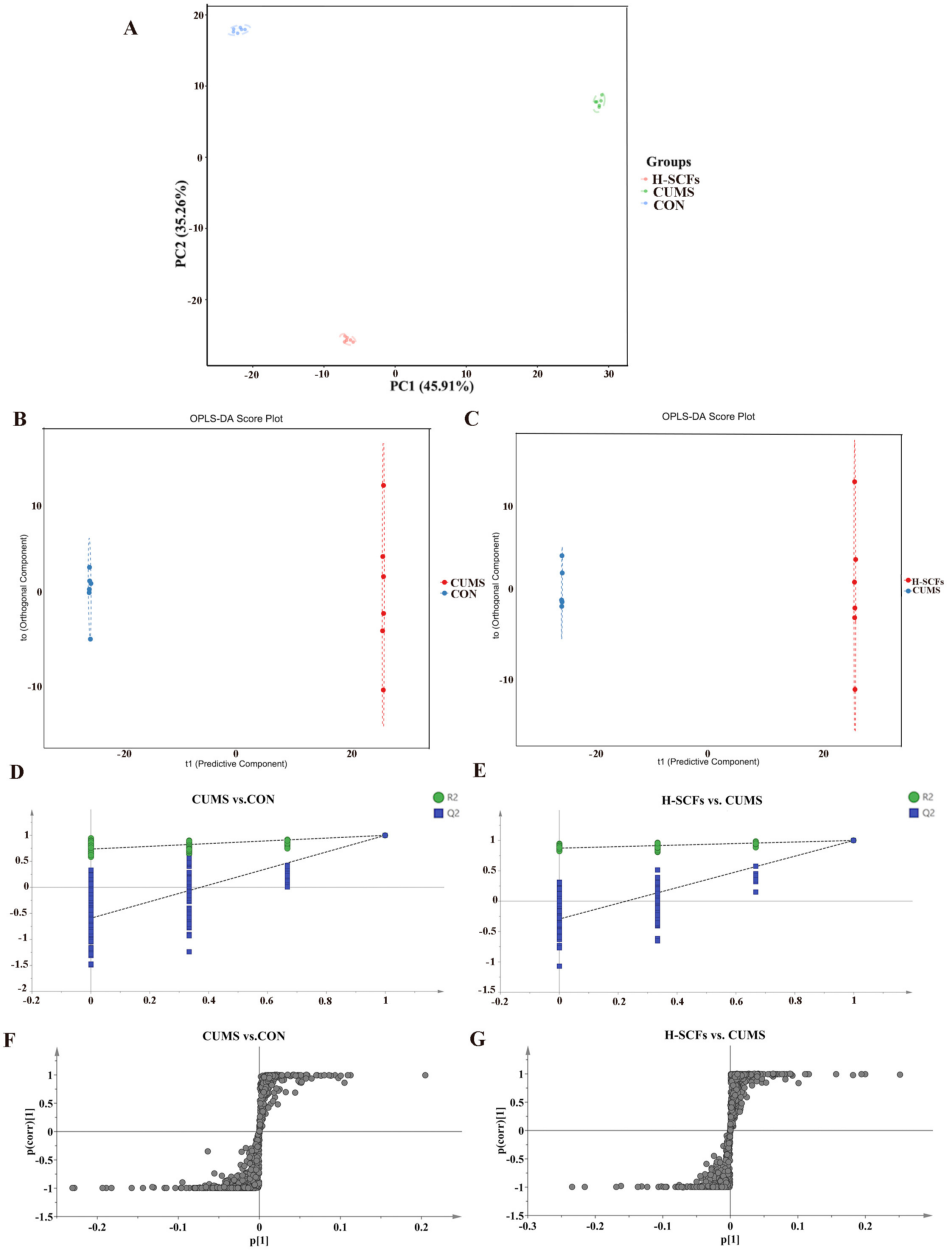
Effects of H-SCFs on the metabolic profile of feces in CUMS mice. **(A)** PCA score plot of fecal samples from CON, CUMS, and H-SCFs Groups. **(B)** Fecal OPLS-DA score of CUMS and CON groups. **(C)** Fecal OPLS-DA score of H-SCFs and CUMS groups. **(D, E)** OPLS-DA replacement test (200 times) for CON vs. CUMS and H-SCFs vs. CUMS group. **(F, G)** S-plot for CON vs. CUMS and H-SCFs vs. CUMS in the fecal metabolites.

The PCA score plot of the fecal metabolomics data illustrated the metabolic differences among the CON, CUMS, and H-SCFs groups. Notably, samples from the CON and CUMS groups were significantly separated, with PC1 accounting for 45.91% of the total variance and PC2 explaining 35.26%. This separation indicated that the CUMS-induced depression model results in significant metabolic disruptions. In contrast, the H-SCFs group was positioned closer to the CON group, suggesting that treatment with SCFs effectively reverses some metabolic abnormalities induced by CUMS. These findings supported the potential role of SCFs in alleviating depressive symptoms, highlighting their therapeutic effects on the metabolic profile of mice (Figure 6A).

#### 3.3.2 Identification of metabolite biomarkers in feces among groups

OPLS-DA was used to differentiate metabolite profiles and identify potential biomarker metabolites for the CON/CUMS and CUMS/H-SCFs pairs in feces (Figures 6B, C). The results indicated that the model had high explanatory capacity and reliable predictive performance, with R^2^X = 0.735, R^2^Y =1, and Q^2^ = 0.998 in the CON/CUMS pair; R^2^X = 0.725, R^2^Y =1, and Q^2^ = 0.998 in CUMS/H-SCFs pair, respectively. The OPLS-DA model score plots revealed distinct separation of fecal metabolic profiles among the three groups, indicating significant differences in metabolites. The robustness and statistical significance of the model were confirmed by 200 permutation tests (opls-perm), which showed no overfitting (Figures 6D, E). S-plot analyses were then conducted to identify potential differential metabolites (Figures 6F, G).

The analyses resulted in the identification of 416 metabolites in the CON/CUMS pair and 347 metabolites in the CUMS/H-SCFs pair, respectively, based on a VIP > 1 in the S-Plot and a *t*-test *P*-value < 0.05 (with a fold change > 2 or < 0.50) from the OPLS-DA model. Among the identified metabolites, 239 fecal biomarkers were commonly regulated across the three groups (Supplementary Tables 3–5), suggesting that the changes in these metabolites were potential biomarkers for the effectiveness of SCFs treatment in CUMS mice.

#### 3.3.3. Analyses of metabolic pathways

To further understand the metabolic differences among the three groups in fecal samples, we used MetaboAnalyst 4.0 to perform KEGG enrichment analysis on the differential metabolite data from the CON/CUMS and CUMS/H-SCFs pairs. The analysis identified several significant metabolic pathways across the two pairs (*P* < 0.05). The CON/CUMS pair showed enrichment in pathways related to Aldosterone synthesis and secretion, Arachidonic acid metabolism, Cortisol synthesis and secretion, Cushing syndrome, parathyroid hormone synthesis, secretion, and action, and Vascular smooth muscle contraction (Supplementary Table 6). Meanwhile, in the CUMS/H-SCFs pair, the enriched pathways are Taurine and hypotaurine metabolism, Biosynthesis of unsaturated fatty acids, and Primary bile acid biosynthesis, all of which can be classified as lipid metabolism-related pathways (Supplementary Table 7). Based on the results of the KEGG enrichment analysis, we found that the differential metabolites between the CON and CUMS groups in fecal samples were significantly enriched in the “aldosterone synthesis and secretion” pathway. Additionally, the two metabolite datasets identified KEGG pathways showed substantial enrichment in numerous pathways related to “lipid metabolism.” This indicated that these metabolic pathways may play important roles in the development of depression and the efficacy of SCFs in antidepressant treatment.

**Figure 7:**
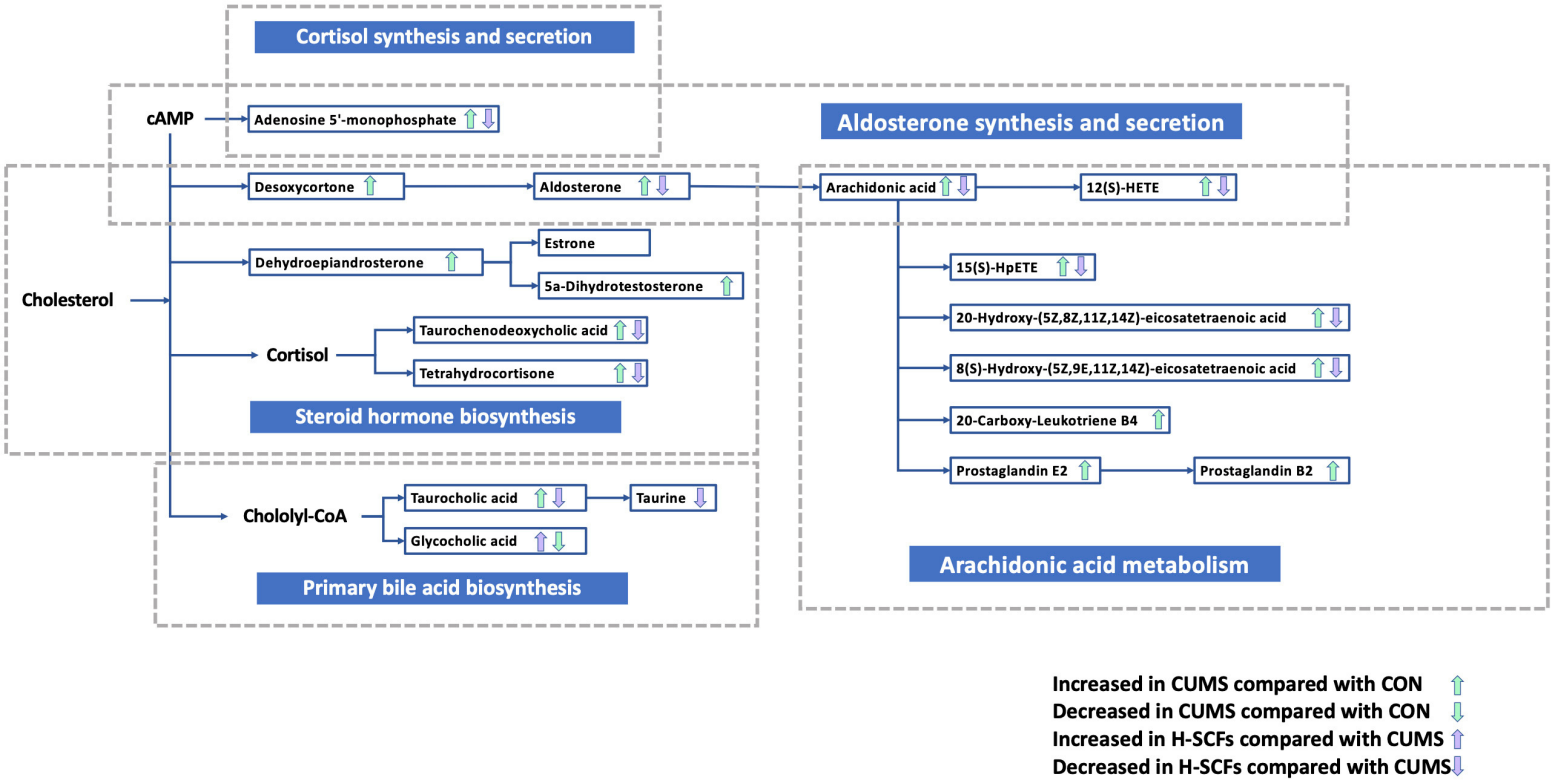
The perturbed major metabolic pathways of the fecal samples. Metabolites are annotated to indicate significant changes between groups. The green upward (↑) and downward (↓) arrows represent metabolites increased or decreased in the CUMS group compared with the CON group, respectively. The blue upward (↑) and downward (↓) arrows represent metabolites increased or decreased in the H-SCFs group compared with the CUMS group, respectively. The metabolic pathways highlighted the effects of CUMS and the therapeutic impact of SCFs on gut microbiota-associated metabolites in the fecal samples.

Consequently, we created a metabolic pathway diagram based on these KEGG relationships (Figure 7). The results revealed changes in lipid metabolism pathways, particularly focusing on steroid metabolism and arachidonic acid pathways, which are central to lipid metabolism. These metabolites may serve as potential biomarkers, highlighting the relevant changes in lipid metabolism driven by these pathways.

#### 3.3.4 Correlations between potential microbial biomarkers and differential metabolites involving the anti-depression effects of SCFs

**Figure 8:**
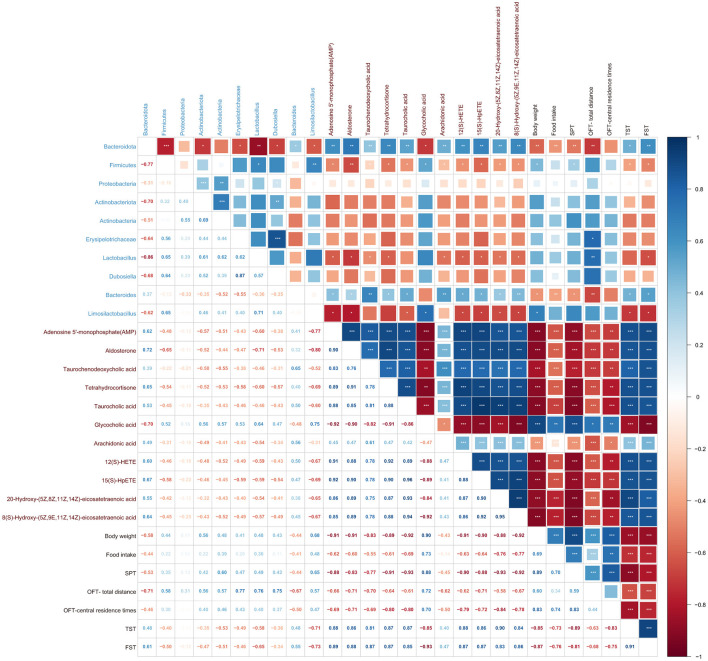
Spearman correlations analysis illustrating the relationships among gut microbiota, fecal metabolites, and depression-related indicators. Heatmap of Spearman correlations among microbial taxa, metabolic pathways, and depression-related indicators. Colors indicate correlation direction and strength (blue: positive, red: negative), with square sizes reflecting magnitude. Labels represent microbial taxa (blue), pathways (brown), and phenotypes (black). Significant correlations are annotated. Hierarchical clustering highlights related patterns.

To further investigate the correlations among the gut microbiota, fecal metabolites, and depression-related indicators, we analyzed the Spearman correlation (Figure 8). *Bacteroidota* and *Bacteroides* were negatively correlated with body weight, food intake, SPT, and OFT-total distance, but positively correlated with TST and FST. *Firmicutes* showed positive correlations with body weight and SPT, and negative correlations with TST and FST. *Lactobacillus* was positively correlated with OFT-total distance and negatively correlated with FST. *Limosilactobacillus* was positively correlated with body weight but negatively correlated with TST and FST. Additionally, *Bacteroidota* was negatively correlated with *Lactobacillus, Firmicutes, Actinobacteriota, Dubosiella, Erysipelotrichaceae*, and *Limosilactobacillus*. *Firmicutes* were positively correlated with *Lactobacillus* and *Limosilactobacillus*.

AMP, Aldosterone, Taurochenodeoxycholic acid, Tetrahydrocortisone, Taurocholic acid, Arachidonic acid, 12(S)-HETE, 15(S)-HpETE, 20-Hydroxy-(5Z,8Z,11Z,14Z)-eicosatetraenoic acid, and 8(S)-Hydroxy-(5Z,9E,11Z,14Z)-eicosatetraenoic acid were negatively correlated with body weight, food intake, SPT, OFT-total distance, and OFT-central residence times, but positively correlated with TST and FST. Glycocholic acid was positively correlated with body weight, food intake, SPT, OFT-total distance, and OFT-central residence times, but negatively correlated with TST and FST.

*Firmicutes*, including its genera *Lactobacillus* and *Limosilactobacillus*, showed significant negative correlations with the fecal metabolites such as Aldosterone, AMP, 12(S)-HETE, 20-Hydroxy-(5Z,8Z,11Z,14Z)-eicosatetraenoic acid, 8(S)-Hydroxy-(5Z,9E,11Z,14Z)-eicosatetraenoic acid, and Taurocholic acid, while exhibiting a significant positive correlation with Glycocholic acid. Similarly, *Bacteroidota* and its genus *Bacteroides* showed strong positive correlations with metabolites including Taurochenodeoxycholic acid, Arachidonic acid, Taurocholic acid, 12(S)-HETE, 15(S)-HpETE, 8(S)-Hydroxy-(5Z,9E,11Z,14Z)-eicosatetraenoic acid, AMP, Tetrahydrocortisone, 20-Hydroxy-(5Z,8Z,11Z,14Z)-eicosatetraenoic acid, and Aldosterone, while being significantly negatively correlated with Glycocholic acid (Supplementary Table 8).

## 4 Discussion

Recent studies have demonstrated a robust correlation between gut dysbiosis and both the onset and progression of depression (Qu et al., 2019; Lach et al., 2018), with the microbiome playing a pivotal role in modulating the condition (Zheng et al., 2016; Kelly et al., 2016b). Stress can induce alterations in animals' fecal microbiota, neuroendocrine dysregulation, and gut microbial metabolic disorders (O'Mahony et al., 2009; Maes et al., 2008; Dunlop et al., 2003). The microbiota-gut-brain axis plays a crucial role, and feces, as the end product of both metabolism and gut microbiota activity, can reflect the improvement of depression and its underlying mechanisms of action (Loh et al., 2024). Our preliminary research indicated that 28 days of CUMS exposure effectively established a depression model in mice, accompanied by hippocampal metabolic disorders, which were significantly ameliorated by a high dose of SCFs, along with the alleviation of depressive behaviors (Shao et al., 2024). In this study, the same CUMS modeling method was employed to establish the depression mice model, and UHPLC-MS/MS-based non-targeted metabolomics and 16S rRNA sequencing technologies were utilized to elucidate the mechanisms underlying the antidepressant effects of SCFs, with a focus on fecal microbiota and metabolites.

Behavioral assessments of depressed mice are crucial tools in depression research, aiding in advancing our understanding of the pathological mechanisms underlying depression. Anhedonia, a core symptom of depression, is typically measured by SPT and food consumption (Qiao et al., 2020), and can also be assessed through the OFT. Other core symptoms of depression, including reduced exploratory behavior, diminished activity, and feelings of despair, are primarily evaluated using the OFT, TST, and FST. Additionally, rectal temperature measurements indicated that prolonged stress may increase energy expenditure and disrupt metabolism, leading to a decrease in body temperature. In this study, the results showed that mice exposed to 28-day CUMS reduced body weight, food intake, and sucrose preference rate compared to the CON group. The CUMS group also exhibited significantly reduced exploratory behavior and rectal temperature, along with increased helplessness. Notably, SCFs intervention, particularly at high doses, almost completely reversed these stress-induced behavioral impairments, underscoring their therapeutic potential. Furthermore, the behavioral recovery correlated with changes in gut microbiota, indicating that SCFs may exert antidepressant effects by modulating the gut microbiota. The finding that CUMS mice had reduced body weight than the control normal mice is consistent with a previous report (Shin et al., 2024). The finding that SCFs-treated mice exhibited increased body weight gain, is intriguing. In humans, the relationship between depression and body weight gain appears to be quite complex and can be affected by a number of variables including age, gender, body mass index, etc (Sutin and Zonderman, 2012; Jung et al., 2017). Perhaps, in the CUMS mice that were derived from relatively young male mice, reduction in body weight gain reflected a typical response of depression, while the SCFs treatment then ameliorated the depressive symptoms and consequently leading to the restoration toward the normal body weight gain trend.

A previous study compared the gut microbiota of CUS mice before and after the treatment with *Semen Cuscuta* extract, demonstrating that the treatment increased the relative abundance of *Firmicutes* and *Lactobacillus* in the CUS model (Hou et al., 2023). In the present study, we found that CUMS mice exhibited alterations in gut microbiota compared to their normal counterparts, which aligns with findings in humans that depression is associated with significant changes in gut microbiota. Notably, by analyzing and comparing the gut microbiota profiles of both CUMS and normal mice, we found that SCFs treatment diminished these changes. This finding clearly indicates that the primary effect of SCFs on the gut microbiota is the restoration of its homeostasis, aligning it with that of normal animals, rather than inducing a completely different composition.

Specifically, we found that the microbiota dysbiosis in the CUMS mice is characterized by an increase in *Bacteroidetes* and *Proteobacteria* and a marked reduction in *Firmicutes, Actinobacteria*, and *Patescibacteria*. These findings align with previous reports linking depression to gut microbiota dysbiosis, characterized by alterations in the abundance of several microbial phyla present in both rodents and humans (Qiu et al., 2017; Jiang et al., 2015). Including an increased relative abundance of *Bacteroidetes* (*Bacteroidota*; Liu et al., 2020) and Proteobacteria (Jiang et al., 2015) and a decreased abundance of *Firmicutes* (Jiang et al., 2015; Huang et al., 2018) and *Actinobacteria* (Jiang et al., 2015). Most significantly, our study clearly demonstrated that SCFs treatment reversed these changes.

Subsequently, this study employed LEfSe analysis to identify unique microbial characteristics specific to each group, highlighting the impact of chronic stress and the potential therapeutic effects of SCFs on gut microbiota composition. *Bacteroides_acidifaciens*, along with the *Bacteroides* genus, *Bacteroidaceae* family, *Bacteroidia* class, and *Bacteroidetes* phylum, emerged as a dominant species in the CUMS group, exhibiting significant upregulation. This finding aligns with previous reports of elevated *Bacteroides* levels in depression patients (Zhang et al., 2022). This increase in *Bacteroides* abundance is associated with heightened inflammation, contributing to the exacerbation of depressive symptoms (Jiang et al., 2015). In contrast, SCFs intervention significantly downregulated these taxa, suggesting their potential role in reducing inflammation and improving depressive outcomes.

SCFs also increased the abundance of beneficial bacteria, including *Lactobacillus, Limosilactobacillus*, and *Actinobacteria*. These bacteria are known for their anti-inflammatory properties (Ye et al., 2024; Li et al., 2024; Hashikawa-Hobara et al., 2022). *Lactobacillus* is a prominent probiotic genus recognized for its positive effects on the central nervous system and its potential to alleviate depression (Aizawa et al., 2016; Hashikawa-Hobara et al., 2022). Studies have demonstrated that the abundance of *Lactobacillus* is significantly decreased in patients with major depression (MDD; Bravo et al., 2011; Herp et al., 2019), and oral intake of *Lactobacillus* can improve stress-induced behaviors and mild depression (Marin et al., 2017), while also enhance the richness and diversity of the gut microbiota (Xiao et al., 2021). Additionally, *Lactobacillus_reuteri*, a key species within this genus, has been shown to improve stress-induced behaviors and alterations in serotonin metabolism induced by chronic social defeat stress (Xie et al., 2020), further supporting the role of *Lactobacillus* in alleviating depression.

SCFs also enriched specific taxa capable of producing short-chain fatty acids (SCFAs), such as *Erysipelotrichaceae, Lactobacillus, Dubosiella*, and *Actinobacteria*, which are strongly implicated in alleviating depression (Hadinia et al., 2022; Estaki et al., 2016; Chen et al., 2022; Liu et al., 2023; Wei et al., 2024). SCFAs, components of lipid metabolism, are key mediators in the gut-brain axis (Oleskin and Shenderov, 2019) and ameliorate inflammatory responses in the central nervous system (Fung et al., 2017; Tankou et al., 2018; Tian et al., 2019; Maslowski and Mackay, 2011). Additionally, *Actinobacteria* play a crucial role in maintaining gut homeostasis, as their ability to produce substantial quantities of SCFAs supports the integrity of the gut barrier (Binda et al., 2018). These results suggest that the antidepressant effects of SCFs may be related to lipid metabolism, particularly through their influence on SCFA production and the abundance of associated microbiota. Moreover, our correlation analysis indicates that the strains regulated by SCFs are significantly associated with improvements in depressive behaviors.

Our metabolomics analysis revealed significant alterations in the metabolic pathways of both the gut and its microbiota. In the gut, several pathways were affected, particularly those involved in aldosterone synthesis and secretion, arachidonic acid metabolism, and primary bile acid biosynthesis, and the dysregulation of these pathways were closely associated with depression. For example, depression is closely associated with the abnormal regulation of aldosterone synthesis and secretion pathway, particularly in terms of neuroendocrine function and inflammatory responses (Franklin et al., 2015). Excessive activation of the HPA axis may lead to increased aldosterone levels, which intensify the body's stress response and negatively affect mood by disrupting electrolyte balance and blood pressure regulation (Zhou et al., 2022). In this study, the levels of AMP and aldosterone in the fecal samples of the CUMS mice were higher compared to the CON group, while these levels were reversed following SCFs treatment. The upregulation of AMP and aldosterone in the CUMS group likely reflected the overactivation of the HPA axis, a typical characteristic of depression, and is associated with disruptions in electrolyte and blood pressure regulation, as well as increased oxidative stress and inflammatory responses, which may also indicate abnormalities in cellular energy metabolism and signal transduction (Vargas et al., 2001; Daimon et al., 2016). SCFs treatment decreased AMP and aldosterone levels, suggesting that SCFs effectively alleviated the overactivation of the HPA axis, improved cellular metabolism and signal transduction, restored physiological balance, and reduced oxidative stress and inflammation, thereby contributing to the improvement of depressive symptoms. This study also found a negative correlation between body weight and AMP and aldosterone levels. Dietary AMP supplementation may enhance oxygen consumption and energy expenditure (Wu et al., 2022), potentially explaining the weight loss observed in CUMS mice.

Correlation analysis revealed that *Firmicutes*, including the genera *Lactobacillus* and *Limosilactobacillus*, were negatively correlated with AMP and aldosterone, suggesting that the antidepressant effects of SCFs treatment are associated with the abundance of *Firmicutes, Lactobacillus*, and *Limosilactobacillus*. However, further studies are needed to determine the relationship between these two effects of SCFs treatment.

In the arachidonic acid (AA) metabolism pathway, it was found that the levels of AA and its metabolites, such as 12(S)-HETE, 15(S)-HpETE, 20-Hydroxy-(5Z,8Z,11Z,14Z)-eicosatetraenoic acid, and 8(S)-Hydroxy-(5Z,9E,11Z,14Z)-eicosatetraenoic acid, were higher in the fecal samples of CUMS mice compared to the CON group, while the levels were lower in the SCFs group compared to the CUMS group. Our correlation analysis reveals that *Bacteroidota* and its genus *Bacteroides* exhibited strong positive correlations with the aforementioned metabolites, while *Firmicutes*, including its genera *Lactobacillus* and *Limosilactobacillus*, showed negative correlations with 12(S)-HETE, 20-Hydroxy-(5Z,8Z,11Z,14Z)-eicosatetraenoic acid, and 8(S)-Hydroxy-(5Z,9E,11Z,14Z)-eicosatetraenoic acid. Metabolites of AA are typically produced through the cyclooxygenase (COX) and lipoxygenase (LOX) pathways, and the activation of these pathways is associated with various inflammatory diseases and depression (Kursun et al., 2022; Gorica and Calderone, 2022). AA and its relative metabolites played a crucial role in the mediation of inflammation (Meng et al., 2015; Gorica and Calderone, 2022), and the increase in these metabolites in the CUMS group likely reflect an enhanced inflammatory response in the gut, consistent with the inflammatory hypothesis of depression. In addition, the metabolites produced in the AA metabolic pathway have the ability to promote oxidative stress (Bao et al., 2023), and the observed increase in the levels of these metabolites in fecal samples may indicate heightened oxidative stress in the state of depression.

Furthermore, in the primary bile acid biosynthesis pathway, two metabolites exhibited with significant differences among the three groups. The increased levels of taurocholic acid and decreased levels of glycocholic acid in the CUMS group indicate that bile acid metabolism was affected by stress. In contrast, the SCFs group showed reduced taurocholic acid levels and restored glycocholic acid levels, suggesting that SCFs regulate bile acid metabolism and contribute to the recovery of gut function. The primary bile acids, chenodeoxycholic acid (CDCA) and cholic acid (CA), are synthesized in the liver (Russell and Setchell, 1992; Chiang, 2009) and are conjugated to either glycine or taurine before being released into the intestine via the gallbladder (Hofmann, 1999; Chiang, 2009). Within the intestine, bile acids not only play a critical role in the normal physiology of the intestine, but also exhibited a complex bidirectional relationship with the gut microbiota. First, the primary bile acids can be converted to secondary bile acids, such as deoxycholic acid (DCA), and lithocholic acid (LCA; Hofmann, 1999). On the other hand, the composition and the relative concentrations of the bile acids and their derivative affect the structure and the homeostasis of the microbiota. For examples, bile acids can exert direct influences, both positive and negative, on gut bacteria (Begley et al., 2005). Moreover, bile resistance, tolerance, and susceptibility are strain-specific (Chateau et al., 1994; Zárate et al., 2000). For examples, gram-negative bacteria are currently thought to be more resistant to bile acids than Gram-positive microorganisms (Begley et al., 2005); while primary bile acids may also serve as a control mechanism to prevent outgrowth of pathogenic Gram-negative bacteria in the small intestine (Kakiyama et al., 2013). The concentration of CA can also regulate gut community structure. Specifically, increased CA concentrations resulted in increases in Firmicutes, and a decrease of Bacteroidetes (Islam et al., 2011; Ridlon et al., 2013). Furthermore, bile acids can also have indirect antimicrobial effects mediated by FXRα (Inagaki et al., 2006).

Metabolic products derived from the gut microbiota play a pivotal role in gut-brain communication (Lourenço et al., 2019). Studies have shown that microbial-derived compounds like bile acids may impact psychiatric disorders through the gut-microbiota-brain axis (Chang et al., 2022; Hashimoto, 2022). Dysbiosis in the gut microbiota is linked to systemic inflammation, with bile acids serving as key regulators of microbiota composition. Taurine and hypotaurine metabolism are intricately connected to bile acid metabolism, which plays a vital role in lipid digestion and absorption. The biosynthesis of unsaturated fatty acids is directly involved in lipid metabolism, as fatty acids are the fundamental components of lipids. Primary bile acid biosynthesis is a part of lipid metabolism because bile acids are derived from cholesterol and are involved in the digestion and metabolism of lipids. All three pathways are connected to lipid metabolism. Overall, the bile acid-gut-microbiota axis is considered a key modulator of immune function and overall health (Hashimoto, 2022).

Taken together, it appears that SCFs modulate the gut microbiota by promoting beneficial bacteria, including those involved in SCFA production, while reducing pro-inflammatory taxa and restoring microbial diversity and homeostasis. SCFs also significantly modulated several metabolic pathways, including aldosterone synthesis, arachidonic acid metabolism, and bile acid biosynthesis, thereby alleviating neuroendocrine dysfunction and inflammation associated with depression. These findings further support the concept of SCFs as potential candidates for the development of effective antidepressant therapies.

The major components of SCFs include Hyperoside, Quercetin, Astragalin, Kaempferol. These compounds have been reported to alleviate depressive symptoms in mice through various mechanisms. For instance, Hyperoside significantly mitigates depression-like behaviors in chronic stress-induced mice by inhibiting the NLRP1 inflammasome and modulating the CXCL1/CXCR2/BDNF signaling pathway, leading to improved anhedonia and reduced immobility time (Song et al., 2022). Quercetin improves depression-like behaviors by activating the ERK/Nrf2 pathway and reducing neuronal apoptosis, which results in alleviated anhedonia and decreased immobility time (Ge et al., 2024). Astragalin activates the SIRT1 signaling pathway, inhibits NLRP3 inflammasome activation, and reduces neuroinflammation, which manifests as increased sucrose preference and reduced immobility time in the tail suspension and forced swimming tests (Tong et al., 2020). Kaempferol alleviates depression-like behaviors in chronic stress-induced rats by inhibiting autophagy, oxidative stress, and neuroinflammation, thus resulting in reduced anhedonia and immobility time (Zhang et al., 2019). These mechanisms may underlie the antidepressant effects of SCFs, contributing to the improvement of depressive behaviors and restoration of body weight in mice. It remains unclear, however, how might these known mechanisms be related to the mechanisms through which SCFs ameliorated the depressive symptoms in CUMS mice via their effects on gut microbiota. Future experiments are needed to address this question.

This study has certain limitations. First, a single depression model (CUMS) was employed, which may not fully encompass the complexity of depression in humans. Future studies should further validate these findings in other models, such as the chronic social defeat stress model, and explore the effects of SCFs on brain inflammation and specific gut-brain signaling pathways. Moreover, while our study highlights the role of gut microbiota and metabolism, additional experiments are required to establish a causal relationship between these factors and the antidepressant effects of SCFs.

## 5 Conclusion

SCFs exhibit significant antidepressant effects by modulating gut microbiota composition, particularly increasing the abundance of *Firmicutes, Lactobacillus, Limosilactobacillus*, and *Actinobacteria* while reducing *Bacteroidota* and Bacteroides. SCFs also modulate critical metabolic pathways, including aldosterone synthesis and bile acid metabolism, thereby contributing to their therapeutic effects.

This study, for the first time, integrates 16S rRNA sequencing and metabolomics to elucidate the antidepressant mechanisms of SCFs, thereby underscoring their potential as psychobiotic candidates. Future research should investigate the direct effects of SCFs on brain inflammation and their interactions with the gut microbiota, in order to further validate their clinical applicability.

## Data Availability

The original contributions presented in the study are publicly available. Raw and processed data is available through the SRA (NCBI BioProject PRJNA1256388) and the study is also identified in the MetaboLights repository, accession number MTBLS12435. Further inquiries can be directed to the corresponding authors.
