# Supplementary material for: The effects of a semen cuscutae flavonoids-based antidepressant treatment on microbiome and metabolome in mice
Source: Front Microbiol. 2025 May 15;16:1558833. doi: 10.3389/fmicb.2025.1558833 (PMC12119544; doi:10.3389/fmicb.2025.1558833)
Supplement: Supplementary file 1 [file Table_1.docx]

**Supplementary Table 1 CUMS protocol modeling design**

| Weeks/Days | 1 | 2 | 3 | 4 | 5 | 6 | 7 |
| --- | --- | --- | --- | --- | --- | --- | --- |
| 1 | FW+D | R+CT | I+W | C+T | CL+CS | I+O | FW+CL |
| 2 | W+T | FW+C | CL+D | CT+CS | R+I | O+T | CS+D |
| 3 | CL+I | O+W | FW+CT | R+D | C+W | R+CS | CT+T |
| 4 | C+CS | R+O | D+W | FW+O | CL+ T | I+CT | R+C |

All the stressors were applied individually and continuously, with no sequence repetition between weeks and being unpredictable. FW, water and food deprivation (24 h); R reversed light/dark cycle (24 h); CL,24 h constant light; C, confinement in a tube for 2 h; I, ice water swimming (4 ◦C, 1 min); CT, cage tilt (45◦, 12 h); CS, cage shaking for 10 min; O, oven (45 ◦C, 5 min); D, damp bedding (150 mL water +200 g bedding 24 h); W, white noise (85 dB, 5 min); T, tail pinch (1 min).

**Supplementary Table 2 The relative abundance of Firmicutes/Bacteroidota ratio among CON, CUMS and H-SCFs groups**

| **Group** | **Sample** | **Bacteroidota** | **Firmicutes** | **Firmicutes/Bacteroidota** |
| --- | --- | --- | --- | --- |
| CON | 1 | 0.6845 | 0.1438 | 0.2101 |
| CON | 2 | 0.7447 | 0.2362 | 0.3172 |
| CON | 3 | 0.5632 | 0.2810 | 0.4989 |
| CON | 4 | 0.5503 | 0.3532 | 0.6418 |
| CON | 5 | 0.6449 | 0.3380 | 0.5241 |
| CON | 6 | 0.6112 | 0.1811 | 0.2963 |
| CON | 7 | 0.4386 | 0.5444 | 1.2412 |
| X±SD |  | 60.53%±9.99% | 29.68%±13.55% | 53.28%±34.68% |
| CUMS | 1 | 0.7607 | 0.2336 | 0.3071 |
| CUMS | 2 | 0.7540 | 0.0987 | 0.1309 |
| CUMS | 3 | 0.7893 | 0.2054 | 0.2602 |
| CUMS | 4 | 0.7280 | 0.2547 | 0.3499 |
| CUMS | 5 | 0.6949 | 0.2726 | 0.3923 |
| CUMS | 6 | 0.9066 | 0.0408 | 0.0450 |
| CUMS | 7 | 0.8624 | 0.1225 | 0.1420 |
| X±SD |  | 78.51%±7.50% | 17.55%±8.84% | 23.25%±12.86% |
| H-SCFs | 1 | 0.5187 | 0.4478 | 0.8633 |
| H-SCFs | 2 | 0.5897 | 0.2035 | 0.3451 |
| H-SCFs | 3 | 0.6804 | 0.2990 | 0.4394 |
| H-SCFs | 4 | 0.5359 | 0.3983 | 0.7432 |
| H-SCFs | 5 | 0.6625 | 0.2013 | 0.3038 |
| H-SCFs | 6 | 0.4584 | 0.3175 | 0.6926 |
| H-SCFs | 7 | 0.7921 | 0.1904 | 0.2404 |
| X±SD |  | 60.54%±11.40% | 29.40%±10.22% | 51.83%±24.48% |

**Supplementary Table 3 Differential metabolites between the CUMS and CON groups**

| **NO** | **Metabolite** | **t_R_(min)** | **Formula** | **Ionization mode** | **VIP** | **P** | **Fold** |
| --- | --- | --- | --- | --- | --- | --- | --- |
|  |  |  |  |  | **score** | **value** | **change** |
| 1 | Gluconic acid | 1366 | C₆ H₁₂ O₇ | NEG | 1.15348252 | △△△ | 0.037643101 |
| 2 | 2-Hydroxycaproic acid | 6075 | C₆ H₁₂ O₃ | NEG | 1.164545798 | △△△ | 0.051598474 |
| 3 | β-Muricholic acid | 7374 | C₂₄ H₄₀ O₅ | NEG | 1.155827417 | △△△ | 2.108470926 |
| 4 | Phenylacetaldehyde | 5974 | C₈ H₈ O | NEG | 1.160925316 | △△△ | 3.411110352 |
| 5 | (±)11(12)-EET | 8799 | C₂₀ H₃₂ O₃ | NEG | 1.161000648 | △△△ | 0.138373862 |
| 6 | (±)11-HETE | 863 | C₂₀ H₃₂ O₃ | NEG | 1.163964324 | △△△ | 0.086769491 |
| 7 | L-Threonic acid | 1387 | C₄ H₈ O₅ | NEG | 1.144961693 | △△△ | 0.309343991 |
| 8 | N-Acetylaspartic acid | 2038 | C₆ H₉ N O₅ | NEG | 1.164264971 | △△△ | 0.115536202 |
| 9 | 2'-Deoxyinosine | 5095 | C₁₀ H₁₂ N₄ O₄ | NEG | 1.15172293 | △△△ | 3.69893746 |
| 10 | 8(S)-Hydroxy-(5Z,9E,11Z,14Z)-eicosatetraenoic acid | 9028 | C₂₀ H₃₂ O₃ | NEG | 1.152846776 | △△△ | 0.166713008 |
| 11 | Benzoic acid | 5847 | C₇ H₆ O₂ | NEG | 1.150050626 | △△△ | 2.025901173 |
| 12 | Succinic acid | 2956 | C₄ H₆ O₄ | NEG | 1.161455016 | △△△ | 0.248240046 |
| 13 | ent-Prostaglandin F2α | 7055 | C₂₀ H₃₄ O₅ | NEG | 1.159940088 | △△△ | 0.160277578 |
| 14 | D-(-)-Lyxose | 1479 | C₅ H₁₀ O₅ | NEG | 1.160162374 | △△△ | 0.203135976 |
| 15 | 15(S)-HpETE | 8127 | C₂₀ H₃₂ O₄ | NEG | 1.156005901 | △△△ | 0.14101203 |
| 16 | 12-epi Leukotriene B4 | 8451 | C₂₀ H₃₂ O₄ | NEG | 1.164920025 | △△△ | 0.06556012 |
| 17 | 20-Hydroxy-(5Z,8Z,11Z,14Z)-eicosatetraenoic acid | 9721 | C₂₀ H₃₂ O₃ | NEG | 1.149148683 | △△△ | 0.167199809 |
| 18 | 3-Coumaric acid | 5889 | C₉ H₈ O₃ | NEG | 1.117520037 | △△△ | 2.632323457 |
| 19 | Suberic acid | 6037 | C₈ H₁₄ O₄ | NEG | 1.155719363 | △△△ | 0.473209993 |
| 20 | δ-Gluconic acid δ-lactone | 1482 | C₆ H₁₀ O₆ | NEG | 1.162731115 | △△△ | 0.406113243 |
| 21 | Genistein | 6465 | C₁₅ H₁₀ O₅ | NEG | 1.162988968 | △△△ | 0.426848663 |
| 22 | (±)10(11)-EpDPA | 9139 | C₂₂ H₃₂ O₃ | NEG | 1.162010968 | △△△ | 0.191440607 |
| 23 | D-α-Hydroxyglutaric acid | 2131 | C₅ H₈ O₅ | NEG | 1.138277209 | △△△ | 0.396316884 |
| 24 | Adipic acid | 554 | C₆ H₁₀ O₄ | NEG | 1.157402381 | △△△ | 0.154576466 |
| 25 | Glycodeoxycholic acid | 81 | C₂₆ H₄₃ N O₅ | NEG | 1.155302774 | △△△ | 4.468583235 |
| 26 | DL-Tryptophan | 5529 | C₁₁ H₁₂ N₂ O₂ | NEG | 1.117493737 | △△△ | 0.442242043 |
| 27 | 3-Hydroxy-3-methylglutaric acid | 3417 | C₆ H₁₀ O₅ | NEG | 1.161151169 | △△△ | 0.38751292 |
| 28 | Ethyl-β-D-glucuronide | 1487 | C₈ H₁₄ O₇ | NEG | 1.165092527 | △△△ | 0.308237556 |
| 29 | Glycocholic acid | 6816 | C₂₆ H₄₃ N O₆ | NEG | 1.161029144 | △△△ | 40.93484813 |
| 30 | 20-hydroxy Leukotriene B4 | 7006 | C₂₀ H₃₂ O₅ | NEG | 1.164581685 | △△△ | 0.236113133 |
| 31 | Xylitol | 1412 | C₅ H₁₂ O₅ | NEG | 1.121631694 | △△△ | 0.12075218 |
| 32 | trans-Cinmic acid | 6083 | C₉ H₈ O₂ | NEG | 1.14506728 | △△△ | 0.490688414 |
| 33 | 1,2,3-cyclopropanetricarboxylic acid | 2258 | C₆ H₆ O₆ | NEG | 1.151406012 | △△△ | 0.473337955 |
| 34 | 8(S),15(S)-DiHETE | 867 | C₂₀ H₃₂ O₄ | NEG | 1.161544785 | △△△ | 0.052816429 |
| 35 | Thymidine 5'-monophosphate | 295 | C₁₀ H₁₅ N₂ O₈ P | NEG | 1.162403406 | △△△ | 0.0569696 |
| 36 | 4-Hydroxybutyric acid (GHB) | 3905 | C₄ H₈ O₃ | NEG | 1.165466515 | △△△ | 0.339540637 |
| 37 | δ-Ribono-1,4-lactone | 2447 | C₅ H₈ O₅ | NEG | 1.13387885 | △△△ | 0.498240716 |
| 38 | Guanosine monophosphate (GMP) | 1885 | C₁₀ H₁₄ N₅ O₈ P | NEG | 1.115007419 | △△△ | 0.238639104 |
| 39 | Tretinoin | 8384 | C₂₀ H₂₈ O₂ | NEG | 1.083471368 | △△△ | 0.07297909 |
| 40 | Docosahexaenoic acid | 10303 | C₂₂ H₃₂ O₂ | NEG | 1.161178908 | △△△ | 0.206064616 |
| 41 | Taurochenodeoxycholic acid | 7105 | C₂₆ H₄₅ N O₆ S | NEG | 1.160083733 | △△△ | 0.400666865 |
| 42 | D-Raffinose | 1473 | C₁₈ H₃₂ O₁₆ | NEG | 1.154475294 | △△△ | 0.443938185 |
| 43 | Xanthosine | 4175 | C₁₀ H₁₂ N₄ O₆ | NEG | 1.153335659 | △△△ | 2.327613245 |
| 44 | Adenosine 5'-monophosphate | 1813 | C₁₀ H₁₄ N₅ O₇ P | NEG | 1.151739066 | △△△ | 0.147756403 |
| 45 | 13,14-Dihydro-15-keto Prostaglandin A2 | 7262 | C₂₀ H₃₀ O₄ | NEG | 1.151632531 | △△△ | 0.041818514 |
| 46 | Guanosine | 4861 | C₁₀ H₁₃ N₅ O₅ | NEG | 1.153031845 | △△△ | 2.62381992 |
| 47 | Thymidine | 5239 | C₁₀ H₁₄ N₂ O₅ | NEG | 1.138897133 | △△△ | 2.082376152 |
| 48 | Uridine | 2649 | C₉ H₁₂ N₂ O₆ | NEG | 1.144848175 | △△△ | 9.349842207 |
| 49 | 5-Hydroxyindole-3-acetic acid | 5849 | C₁₀ H₉ N O₃ | NEG | 1.16324224 | △△△ | 0.491320459 |
| 50 | ST 24:1;O4;T | 7368 | C₂₆ H₄₅ N O₇ S | NEG | 1.16167541 | △△△ | 0.077047556 |
| 51 | LPE O-16:1 | 10167 | C₂₁ H₄₄ N O₆ P | NEG | 1.166025448 | △△△ | 0.07625374 |
| 52 | 8Z,11Z,14Z-Eicosatrienoic acid | 10875 | C₂₀ H₃₄ O₂ | NEG | 1.151328924 | △△△ | 0.296690117 |
| 53 | LPE O-18:2 | 10348 | C₂₃ H₄₆ N O₆ P | NEG | 1.157501557 | △△△ | 0.056800337 |
| 54 | D-(-)-Fructose | 141 | C₆ H₁₂ O₆ | NEG | 1.163146359 | △△△ | 0.213379275 |
| 55 | LPC 22:6 | 9477 | C₃₀ H₅₀ N O₇ P | NEG | 1.164617007 | △△△ | 0.025108338 |
| 56 | LPC 18:0 | 10752 | C₂₆ H₅₄ N O₇ P | NEG | 1.165016729 | △△△ | 0.078024717 |
| 57 | D-Glucose 6-phosphate | 1237 | C₆ H₁₃ O₉ P | NEG | 1.094736021 | △△△ | 0.023755326 |
| 58 | 17(S)-HpDHA | 8128 | C₂₂ H₃₂ O₄ | NEG | 1.111704815 | △△△ | 0.085177951 |
| 59 | 1-Methylxanthine | 1365 | C₆ H₆ N₄ O₂ | NEG | 1.004775306 | △△△ | 0.129376672 |
| 60 | LPG O-14:1 | 10295 | C₂₀ H₄₁ O₈ P | NEG | 1.081663675 | △△△ | 0.13476158 |
| 61 | LPI 20:4 | 10331 | C₂₉ H₄₉ O₁₂ P | NEG | 1.164837015 | △△△ | 0.2216799 |
| 62 | LPE O-18:3 | 9861 | C₂₃ H₄₄ N O₆ P | NEG | 1.165605415 | △△△ | 0.036869392 |
| 63 | Glycerophospho-N-palmitoyl ethanolamine | 10907 | C₂₁ H₄₄ N O₇ P | NEG | 1.165014525 | △△△ | 0.275161701 |
| 64 | 13,14-Dihydro-15-keto prostaglandin F2α | 7898 | C₂₀ H₃₄ O₅ | NEG | 1.142940543 | △△△ | 0.10373275 |
| 65 | (±)8-HEPE | 8589 | C₂₀ H₃₀ O₃ | NEG | 1.157851495 | △△△ | 0.179068731 |
| 66 | (+/-)5(6)-DiHET | 8601 | C₂₀ H₃₄ O₄ | NEG | 1.163382121 | △△△ | 0.054936098 |
| 67 | (+/-)13-HODE | 9408 | C₁₈ H₃₂ O₃ | NEG | 1.162106017 | △△△ | 0.358045415 |
| 68 | 1a,1b-Dihomo prostaglandin E1 | 8744 | C₂₂ H₃₈ O₅ | NEG | 1.150576272 | △△△ | 2.47151921 |
| 69 | (3-Methoxy-4-hydroxyphenyl)ethylene glycol sulfate | 5617 | C₉ H₁₂ O₇ S | NEG | 1.164174377 | △△△ | 5.164253182 |
| 70 | 12-Epileukotriene B4 | 9083 | C₂₀ H₃₂ O₄ | NEG | 1.165192639 | △△△ | 0.094910268 |
| 71 | (+/-)9,10-dihydroxy-12Z-octadecenoic acid | 8023 | C₁₈ H₃₄ O₄ | NEG | 1.014388774 | △△△ | 0.473104682 |
| 72 | FAHFA 2:0/18:1 | 8935 | C₂₀ H₃₆ O₄ | NEG | 1.13110317 | △△△ | 0.39107215 |
| 73 | LPS 18:1 | 10512 | C₂₄ H₄₆ N O₉ P | NEG | 1.033821439 | △△△ | 0.130962304 |
| 74 | 3-(2-thienyl)-1,2,4-oxadiazole-5-carbohydrazide | 1479 | C₇ H₆ N₄ O₂ S | NEG | 1.073203308 | △△△ | 0.450591194 |
| 75 | (2S)-4-Oxo-2-phenyl-3,4-dihydro-2H-chromen-7-yl beta-D-glucopyranoside | 771 | C₂₁ H₂₂ O₈ | NEG | 1.164317282 | △△△ | 0.059600762 |
| 76 | LPS 15:0 | 9602 | C₂₁ H₄₂ N O₉ P | NEG | 1.161629558 | △△△ | 0.112874703 |
| 77 | 11-dehydro Thromboxane B2 | 6831 | C₂₀ H₃₂ O₆ | NEG | 1.130107069 | △△△ | 0.264276632 |
| 78 | LPG 16:1 | 10262 | C₂₂ H₄₃ O₉ P | NEG | 1.023446484 | △△△ | 0.084008007 |
| 79 | 2-Oxobutyric acid | 2142 | C₄ H₆ O₃ | NEG | 1.138277209 | △△△ | 0.396316884 |
| 80 | NSI-189 | 6552 | C₂₂ H₃₀ N₄ O | NEG | 1.144546272 | △△△ | 0.248857282 |
| 81 | Ala-Leu | 5461 | C₉ H₁₈ N₂ O₃ | NEG | 1.051687617 | △△△ | 0.131776167 |
| 82 | (+/-)7(8)-DiHDPA | 8362 | C₂₂ H₃₄ O₄ | NEG | 1.159191589 | △△△ | 0.166296799 |
| 83 | Acardic acid | 9728 | C₂₂ H₃₆ O₃ | NEG | 1.164743809 | △△△ | 0.0696019 |
| 84 | (±)11(12)-DiHET | 816 | C₂₀ H₃₄ O₄ | NEG | 1.162361383 | △△△ | 0.16975655 |
| 85 | PG 4:0_14:0 | 11035 | C₂₄ H₄₇ O₁₀ P | NEG | 1.16510742 | △△△ | 0.156160356 |
| 86 | Aldosterone | 6703 | C₂₁ H₂₈ O₅ | NEG | 1.159408178 | △△△ | 0.070066531 |
| 87 | (+/-)19(20)-DiHDPA | 8194 | C₂₂ H₃₄ O₄ | NEG | 1.164762061 | △△△ | 0.235192779 |
| 88 | Prostaglandin B2 | 802 | C₂₀ H₃₀ O₄ | NEG | 1.154322023 | △△△ | 0.164298221 |
| 89 | 15(R)-Prostaglandin E2 | 6525 | C₂₀ H₃₂ O₅ | NEG | 1.112195877 | △△△ | 0.315693234 |
| 90 | 3-Methylglutaric acid | 4204 | C₆ H₁₀ O₄ | NEG | 1.163787968 | △△△ | 0.272714767 |
| 91 | Lysopc 17:0 | 10752 | C₂₅ H₅₂ N O₇ P | NEG | 1.164211674 | △△△ | 0.075051487 |
| 92 | 4-Toluenesulfonic acid | 5618 | C₇ H₈ O₃ S | NEG | 1.160833875 | △△△ | 2.663845375 |
| 93 | LPE O-18:1 | 10466 | C₂₃ H₄₈ N O₆ P | NEG | 1.049605017 | △△△ | 0.080643637 |
| 94 | Phloretin | 6409 | C₁₅ H₁₄ O₅ | NEG | 1.165176378 | △△△ | 0.014994986 |
| 95 | LPE O-15:1 | 9769 | C₂₀ H₄₂ N O₆ P | NEG | 1.108860874 | △△△ | 0.352292829 |
| 96 | Hexadecanedioic acid | 7009 | C₁₆ H₃₀ O₄ | NEG | 1.152528867 | △△△ | 2.885154019 |
| 97 | Ursolic acid | 10117 | C₃₀ H₄₈ O₃ | NEG | 1.161072969 | △△△ | 4.589525123 |
| 98 | LPC 20:3 | 99 | C₂₈ H₅₂ N O₇ P | NEG | 1.164537818 | △△△ | 0.085622581 |
| 99 | LPG 18:2 | 10835 | C₂₄ H₄₅ O₉ P | NEG | 1.161721443 | △△△ | 0.16517502 |
| 100 | 16-Hydroxyhexadecanoic acid | 9993 | C₁₆ H₃₂ O₃ | NEG | 1.164601272 | △△△ | 0.376599186 |
| 101 | Uridine 5'-Diphospho-N-Acetylgalactosamine | 1622 | C₁₇ H₂₇ N₃ O₁₇ P₂ | NEG | 1.117343021 | △△△ | 2.671974841 |
| 102 | Thromboxane B3 | 7584 | C₂₀ H₃₂ O₆ | NEG | 1.140956986 | △△△ | 0.15971478 |
| 103 | Stachyose | 1482 | C₂₄ H₄₂ O₂₁ | NEG | 1.163649109 | △△△ | 0.343628342 |
| 104 | LPE O-18:0 | 1109 | C₂₃ H₅₀ N O₆ P | NEG | 1.162283506 | △△△ | 0.057893796 |
| 105 | Pipecolic acid | 5649 | C₆ H₁₁ N O₂ | NEG | 1.161825046 | △△△ | 0.159765903 |
| 106 | Gly-Tyr-Ala | 5597 | C₁₄ H₁₉ N₃ O₅ | NEG | 1.157317034 | △△△ | 7.600982176 |
| 107 | 4-Isopropylbenzoic acid | 5855 | C₁₀ H₁₂ O₂ | NEG | 1.1560874 | △△△ | 2.525151438 |
| 108 | Punicic Acid | 8887 | C₁₈ H₃₀ O₂ | NEG | 1.155988964 | △△△ | 0.073672804 |
| 109 | LPS 18:2 | 9826 | C₂₄ H₄₄ N O₉ P | NEG | 1.164920298 | △△△ | 0.201040486 |
| 110 | DL-m-Tyrosine | 5367 | C₉ H₁₁ N O₃ | NEG | 1.148548884 | △△△ | 0.313042189 |
| 111 | Esculin | 5339 | C₁₅ H₁₆ O₉ | NEG | 1.152178261 | △△△ | 2.768741423 |
| 112 | alpha-D-Glucopyranosyl 2-O-(2-methylbutanoyl)-alpha-D-glucopyranoside | 681 | C₁₇ H₃₀ O₁₂ | NEG | 1.121270485 | △△△ | 3.233331548 |
| 113 | DGMG (18:2) | 9269 | C₃₃ H₅₈ O₁₄ | NEG | 1.126459729 | △△△ | 0.318033149 |
| 114 | PE O-16:1_22:4 | 8705 | C₄₃ H₇₈ N O₇ P | NEG | 1.162034009 | △△△ | 3.27215215 |
| 115 | LPS 14:0 | 9233 | C₂₀ H₄₀ N O₉ P | NEG | 1.157944498 | △△△ | 0.078656129 |
| 116 | 2-[(3S)-1-(Cyclohexylmethyl)-3-pyrrolidinyl]-5-fluoro-1H-benzimidazole | 5486 | C₁₈ H₂₄ F N₃ | NEG | 1.153465781 | △△△ | 0.241190885 |
| 117 | Lauric acid ethyl ester | 8168 | C₁₄ H₂₈ O₂ | NEG | 1.11963336 | △△△ | 0.462882731 |
| 118 | Ethylmalonic acid | 2123 | C₅ H₈ O₄ | NEG | 1.159554547 | △△△ | 0.465052808 |
| 119 | LPG O-15:1 | 10933 | C₂₁ H₄₃ O₈ P | NEG | 1.165184158 | △△△ | 0.267249365 |
| 120 | LPE O-17:1 | 10535 | C₂₂ H₄₆ N O₆ P | NEG | 1.16564898 | △△△ | 0.069640242 |
| 121 | Methyl-beta-galactopyranoside | 1528 | C₇ H₁₄ O₆ | NEG | 1.151070612 | △△△ | 0.179461048 |
| 122 | (±)19(20)-DiHDPA | 7869 | C₂₂ H₃₄ O₄ | NEG | 1.161069024 | △△△ | 0.203703239 |
| 123 | Orotic acid | 1644 | C₅ H₄ N₂ O₄ | NEG | 1.127308012 | △△△ | 0.275676551 |
| 124 | N1-(3-amino-4-chlorophenyl)-2-[2,4-di(tert-pentyl)phenoxy]acetamide | 8123 | C₂₄ H₃₃ Cl N₂ O₂ | NEG | 1.163336289 | △△△ | 2.42083069 |
| 125 | L-Arabinitol | 2418 | C₅ H₁₂ O₅ | NEG | 1.130852135 | △△△ | 0.363036042 |
| 126 | N2-Acetyl-L-ornithine | 1469 | C₇ H₁₄ N₂ O₃ | NEG | 1.155363845 | △△△ | 0.481688221 |
| 127 | LPG 14:0 | 9681 | C₂₀ H₄₁ O₉ P | NEG | 1.165566244 | △△△ | 0.192962119 |
| 128 | Jasmonic acid | 8086 | C₁₂ H₁₈ O₃ | NEG | 1.116690166 | △△△ | 0.057553903 |
| 129 | 8-iso-15-keto Prostaglandin F2α | 529 | C₂₀ H₃₂ O₅ | NEG | 1.09046277 | △△△ | 0.183449688 |
| 130 | Vanillyl alcohol | 5437 | C₈ H₁₀ O₃ | NEG | 1.145579196 | △△△ | 0.228189233 |
| 131 | D-Glucono-1,5-lactone | 1845 | C₆ H₁₀ O₆ | NEG | 1.159938399 | △△△ | 0.234180972 |
| 132 | trans-Petroselinic Acid | 8241 | C₁₈ H₃₄ O₂ | NEG | 1.080765586 | △△△ | 0.445159974 |
| 133 | FAHFA 18:1/3:0 | 11124 | C₂₁ H₃₈ O₄ | NEG | 1.151293102 | △△△ | 0.215839989 |
| 134 | LPC 22:5 | 9752 | C₃₀ H₅₂ N O₇ P | NEG | 1.127230784 | △△△ | 0.477531145 |
| 135 | Pentadecanoic acid | 9135 | C₁₅ H₃₀ O₂ | NEG | 1.14852928 | △△△ | 0.403504785 |
| 136 | 1-(3-phenylpropanoyl)-4-piperidinecarboxylic acid | 7104 | C₁₅ H₁₉ N O₃ | NEG | 1.14587823 | △△△ | 3.149167475 |
| 137 | LPC 18:3 | 9181 | C₂₆ H₄₈ N O₇ P | NEG | 1.104126847 | △△△ | 0.397608272 |
| 138 | Tyrosylalanine | 5514 | C₁₂ H₁₆ N₂ O₄ | NEG | 1.048186168 | △△△ | 0.497999184 |
| 139 | (+/-)8(9)-DiHET | 8212 | C₂₀ H₃₄ O₄ | NEG | 1.165224697 | △△△ | 0.170484463 |
| 140 | Oxytetracycline | 1444 | C₂₂ H₂₄ N₂ O₉ | NEG | 1.098899909 | △△△ | 0.348880043 |
| 141 | LPG 20:3 | 11409 | C₂₆ H₄₇ O₉ P | NEG | 1.144009123 | △△△ | 0.115469054 |
| 142 | Maltotriose | 5011 | C₁₈ H₃₂ O₁₆ | NEG | 1.162848043 | △△△ | 0.307274602 |
| 143 | FAHFA 18:2/20:4 | 10477 | C₃₈ H₆₂ O₄ | NEG | 1.119716822 | △△△ | 2.366193399 |
| 144 | L-Methionine sulfone | 1835 | C₅ H₁₁ N O₄ S | NEG | 1.164220708 | △△△ | 6.157361376 |
| 145 | LPA 21:2 | 9505 | C₂₄ H₄₅ O₇ P | NEG | 1.158195597 | △△△ | 0.248516731 |
| 146 | 4-(4-cyclohexylphenyl)-4-oxobut-2-enoic acid | 1879 | C₁₆ H₁₈ O₃ | NEG | 1.158378923 | △△△ | 0.154799393 |
| 147 | Calcitriol | 9788 | C₂₇ H₄₄ O₃ | NEG | 1.120714812 | △△△ | 0.231689407 |
| 148 | Mevalonic acid | 4694 | C₆ H₁₂ O₄ | NEG | 1.150083135 | △△△ | 0.341760548 |
| 149 | 1-(2,4-diphenyl-2,3-dihydro-1H-1,5-benzodiazepin-1-yl)propan-1-one | 8965 | C₂₄ H₂₂ N₂ O | NEG | 1.163875719 | △△△ | 0.115239745 |
| 150 | 10-Hydroxydecanoic acid | 7292 | C₁₀ H₂₀ O₃ | NEG | 1.137268879 | △△△ | 3.185325956 |
| 151 | FAHFA 16:0/18:2 | 10839 | C₃₄ H₆₂ O₄ | NEG | 1.123879243 | △△△ | 0.354812 |
| 152 | Dl-3,4-Dihydroxymandelic Acid | 5331 | C₈ H₈ O₅ | NEG | 1.154087134 | △△△ | 0.374407943 |
| 153 | Gluconolactone | 206 | C₆ H₁₀ O₆ | NEG | 1.158826594 | △△△ | 0.357733242 |
| 154 | 5-(3-chloro-4-methylanilino)-1-methyl-1H-pyrazol-3-ol | 5248 | C₁₁ H₁₂ Cl N₃ O | NEG | 1.098834002 | △△△ | 0.484324079 |
| 155 | Estriol 17-sulfate | 1916 | C₁₈ H₂₄ O₆ S | NEG | 1.125019876 | △△△ | 0.117557618 |
| 156 | Kojic acid | 1572 | C₆ H₆ O₄ | NEG | 1.113721302 | △△△ | 0.432540009 |
| 157 | 2-Hydroxy-2-methylbutanedioic acid | 5641 | C₅ H₈ O₅ | NEG | 1.137865362 | △△△ | 0.438122629 |
| 158 | (+/-)-CP 47,497-C7-Hydroxy metabolite | 9622 | C₂₁ H₃₄ O₃ | NEG | 1.146726353 | △△△ | 0.158102074 |
| 159 | α,α-Trehalose | 1924 | C₁₂ H₂₂ O₁₁ | NEG | 1.163446468 | △△△ | 0.35471549 |
| 160 | Cyclic ADP-ribose | 1837 | C₁₅ H₂₁ N₅ O₁₃ P₂ | NEG | 1.13398209 | △△△ | 3.324430267 |
| 161 | N-(4-chlorophenyl)-N'-(2-phenoxyphenyl)urea | 2011 | C₁₉ H₁₅ Cl N₂ O₂ | NEG | 1.154021111 | △△△ | 0.056086338 |
| 162 | ethyl 3-cyano-2-hydroxy-6-phenylisonicotite | 5643 | C₁₅ H₁₂ N₂ O₃ | NEG | 1.153010582 | △△△ | 0.411700645 |
| 163 | Glycerol-3-phosphate | 1393 | C₃ H₉ O₆ P | NEG | 1.156363163 | △△△ | 0.112705411 |
| 164 | N,5-Bis(3-(trifluoromethyl)phenyl)oxazol-2-amine | 1504 | C₁₇ H₁₀ F₆ N₂ O | NEG | 1.07900499 | △△△ | 0.224459034 |
| 165 | Methyl alpha-D-glucopyranoside | 1893 | C₇ H₁₄ O₆ | NEG | 1.164887368 | △△△ | 0.147225939 |
| 166 | nor-6β-Oxycodol | 3974 | C₁₇ H₂₁ N O₄ | NEG | 1.152260967 | △△△ | 0.377249798 |
| 167 | N-(4-chlorophenethyl)-1-adamantanecarboxamide | 3712 | C₁₉ H₂₄ Cl N O | NEG | 1.164463769 | △△△ | 0.341016983 |
| 168 | Reduced glutathione | 5529 | C₁₀ H₁₇ N₃ O₆ S | NEG | 1.127243592 | △△△ | 0.305027863 |
| 169 | 5-amino-1-(4-chlorobenzyl)-1H-1,2,3-triazole-4-carboxamide | 5367 | C₁₀ H₁₀ Cl N₅ O | NEG | 1.150683502 | △△△ | 0.285190372 |
| 170 | D-Glucosamine 6-phosphate | 1296 | C₆ H₁₄ N O₈ P | NEG | 1.129259736 | △△△ | 0.277424646 |
| 171 | LPC 18:1 | 1014 | C₂₆ H₅₂ N O₇ P | NEG | 1.165314415 | △△△ | 0.217341577 |
| 172 | LPC 16:0 | 9951 | C₂₄ H₅₀ N O₇ P | NEG | 1.165709354 | △△△ | 0.12071356 |
| 173 | Trehalose | 5061 | C₁₂ H₂₂ O₁₁ | NEG | 1.150371523 | △△△ | 0.348565906 |
| 174 | MAG (18:3) | 8449 | C₂₁ H₃₆ O₄ | NEG | 1.12118504 | △△△ | 0.259495272 |
| 175 | LPE 18:0 | 10593 | C₂₃ H₄₈ N O₇ P | NEG | 1.163353952 | △△△ | 0.03618026 |
| 176 | Isorhapontigenin | 6638 | C₁₅ H₁₄ O₄ | NEG | 1.14786468 | △△△ | 0.384673301 |
| 177 | (+/-)12(13)-DiHOME | 1046 | C₁₈ H₃₄ O₄ | NEG | 1.15012888 | △△△ | 0.399960253 |
| 178 | Ethylmalote | 1472 | C₅ H₈ O₄ | NEG | 1.122970859 | △△△ | 0.330434886 |
| 179 | LPE 18:2 | 9521 | C₂₃ H₄₄ N O₇ P | NEG | 1.159538636 | △△△ | 0.265049981 |
| 180 | ringenin | 6267 | C₁₅ H₁₂ O₅ | NEG | 1.164523621 | △△△ | 0.265269024 |
| 181 | Arachidonic acid | 10371 | C₂₀ H₃₂ O₂ | NEG | 1.159118743 | △△△ | 0.322465168 |
| 182 | 7-Hydroxy-3,4-dihydrocarbostyril | 5635 | C₉ H₉ N O₂ | NEG | 1.045083721 | △△△ | 0.336305785 |
| 183 | LPC 16:1 | 9347 | C₂₄ H₄₈ N O₇ P | NEG | 1.151662954 | △△△ | 0.029093094 |
| 184 | N-lactoyl-phenylalanine | 6136 | C₁₂ H₁₅ N O₄ | NEG | 1.143342131 | △△△ | 0.43700856 |
| 185 | Oxoadipic Acid | 1541 | C₆ H₈ O₅ | NEG | 1.126762104 | △△△ | 0.375772167 |
| 186 | Acetylcarnitine | 6137 | C₉ H₁₇ N O₄ | NEG | 1.113972537 | △△△ | 0.473883516 |
| 187 | 2-(Dimethylamino)Guanosine | 1922 | C₁₂ H₁₇ N₅ O₅ | NEG | 1.163480851 | △△△ | 0.181835717 |
| 188 | LysoPE 18:2 | 10272 | C₂₃ H₄₄ N O₇ P | NEG | 1.165084025 | △△△ | 0.179243456 |
| 189 | Porphobilinogen | 5291 | C₁₀ H₁₄ N₂ O₄ | NEG | 1.158635589 | △△△ | 6.165190246 |
| 190 | LPE 20:5 | 9059 | C₂₅ H₄₂ N O₇ P | NEG | 1.152516609 | △△△ | 0.128080261 |
| 191 | 10-Nitrolinoleate | 7032 | C₁₈ H₃₁ N O₄ | NEG | 1.159122828 | △△△ | 0.325682322 |
| 192 | Kinetin 9-riboside | 5501 | C₁₅ H₁₇ N₅ O₅ | NEG | 1.139035553 | △△△ | 0.048215094 |
| 193 | Lysopg 18:1 | 9071 | C₂₄ H₄₇ O₉ P | NEG | 1.161719776 | △△△ | 0.383296049 |
| 194 | 5-Hydroxytryptophan | 3646 | C₁₁ H₁₂ N₂ O₃ | NEG | 1.16348884 | △△△ | 0.053712193 |
| 195 | 13-Hpotre(R) | 7865 | C₁₈ H₃₀ O₄ | NEG | 1.164334869 | △△△ | 0.274939465 |
| 196 | Nonoic acid | 6162 | C₉ H₁₈ O₂ | NEG | 1.162217466 | △△△ | 0.481819487 |
| 197 | 2-Phenylpropionic acid | 5915 | C₉ H₁₀ O₂ | NEG | 1.135820781 | △△△ | 0.268269374 |
| 198 | Hydrocortisone | 6256 | C₂₁ H₃₀ O₅ | NEG | 1.164716773 | △△△ | 0.155904107 |
| 199 | 6-Deoxy-D-glucose | 1532 | C₆ H₁₂ O₅ | NEG | 1.157932932 | △△△ | 0.328346984 |
| 200 | Lysope 14:0 | 6579 | C₁₉ H₄₀ N O₇ P | NEG | 1.142573405 | △△△ | 2.065650981 |
| 201 | 23-Nordeoxycholic acid | 9084 | C₂₃ H₃₈ O₄ | NEG | 1.163896434 | △△△ | 6.485012844 |
| 202 | 5-Methyluridine | 4148 | C₁₀ H₁₅ N₃ O₅ | NEG | 1.15585417 | △△△ | 3.917055715 |
| 203 | 20-Carboxy-Leukotriene B4 | 6089 | C₂₀ H₃₀ O₆ | NEG | 1.149953505 | △△△ | 0.391175286 |
| 204 | LPE 22:6 | 9402 | C₂₇ H₄₄ N O₇ P | NEG | 1.162659577 | △△△ | 0.101031936 |
| 205 | Stearic acid | 1166 | C₁₈ H₃₆ O₂ | NEG | 1.146545018 | △△△ | 0.415400389 |
| 206 | LPC 20:1 | 10936 | C₂₈ H₅₆ N O₇ P | NEG | 1.162182368 | △△△ | 0.087489784 |
| 207 | LysoPE 18:0 | 9949 | C₂₃ H₄₈ N O₇ P | NEG | 1.165662224 | △△△ | 0.115577105 |
| 208 | 1-Methylguanosine | 1486 | C₁₁ H₁₅ N₅ O₅ | NEG | 1.14272369 | △△△ | 0.339309995 |
| 209 | Cytidine-5'-monophosphate | 1499 | C₉ H₁₄ N₃ O₈ P | NEG | 1.098000952 | △△△ | 0.413115863 |
| 210 | 4-Pregnen-17alpha,20alpha-Diol-3-One | 8703 | C₂₁ H₃₂ O₃ | NEG | 1.152713611 | △△△ | 0.085309745 |
| 211 | LPE 20:3 | 9793 | C₂₅ H₄₆ N O₇ P | NEG | 1.159512853 | △△△ | 0.064233817 |
| 212 | Phenylacetylglutamine | 5908 | C₁₃ H₁₆ N₂ O₄ | NEG | 1.142270182 | △△△ | 3.651912142 |
| 213 | 11-Ketoetiocholanolone | 8085 | C₁₉ H₂₈ O₃ | NEG | 1.148056582 | △△△ | 0.087949224 |
| 214 | Estradiol Benzoate | 7924 | C₂₅ H₂₈ O₃ | NEG | 1.127950436 | △△△ | 0.338796568 |
| 215 | Methyltestosterone | 10461 | C₂₀ H₃₀ O₂ | NEG | 1.165314678 | △△△ | 0.13710121 |
| 216 | gamma-Glutamylcysteine | 5305 | C₈ H₁₄ N₂ O₅ S | NEG | 1.067046209 | △△△ | 3.207128585 |
| 217 | Dehydroepiandrosterone | 7843 | C₁₉ H₂₈ O₂ | NEG | 1.022225122 | △△△ | 0.072792934 |
| 218 | LPE 18:1 | 10023 | C₂₃ H₄₆ N O₇ P | NEG | 1.165224277 | △△△ | 0.092561743 |
| 219 | Corey Lactone Diol | 5903 | C₈ H₁₂ O₄ | NEG | 1.139831498 | △△△ | 0.462946242 |
| 220 | Asaraldehyde | 5922 | C₁₀ H₁₂ O₄ | NEG | 1.13618029 | △△△ | 0.271346941 |
| 221 | Prostaglandin E2 | 87 | C₂₀ H₃₂ O₅ | NEG | 1.161338601 | △△△ | 0.032874772 |
| 222 | Glu-Glu | 1506 | C₁₀ H₁₆ N₂ O₇ | NEG | 1.141903507 | △△△ | 0.442811382 |
| 223 | 2-Amino-1,3,4-octadecanetriol | 8404 | C₁₈ H₃₉ N O₃ | POS | 1.164753209 | △△△ | 0.109907071 |
| 224 | Cortisol | 6802 | C₂₁ H₃₀ O₅ | POS | 1.161534527 | △△△ | 0.056666282 |
| 225 | 4-Phenylbutyric acid | 10008 | C₁₀ H₁₂ O₂ | POS | 1.138406929 | △△△ | 0.355854191 |
| 226 | 5,7-dihydroxy-3-(4-hydroxyphenyl)-4H-chromen-4-one | 6095 | C₁₅ H₁₀ O₅ | POS | 1.162657462 | △△△ | 0.232724267 |
| 227 | N6,N6,N6-Trimethyl-L-lysine | 129 | C₉ H₂₀ N₂ O₂ | POS | 1.159804454 | △△△ | 0.319726519 |
| 228 | 3'-Adenosine monophosphate (3'-AMP) | 1817 | C₁₀ H₁₄ N₅ O₇ P | POS | 1.091464818 | △△△ | 0.346520525 |
| 229 | Creatine | 1444 | C₄ H₉ N₃ O₂ | POS | 1.164341381 | △△△ | 0.034284015 |
| 230 | Daidzein | 627 | C₁₅ H₁₀ O₄ | POS | 1.165468954 | △△△ | 0.340813054 |
| 231 | Eicosapentaenoic acid | 8661 | C₂₀ H₃₀ O₂ | POS | 1.163902116 | △△△ | 0.201924047 |
| 232 | Glycitein | 6293 | C₁₆ H₁₂ O₅ | POS | 1.165628591 | △△△ | 0.305509719 |
| 233 | N-Acetylhistamine | 1897 | C₇ H₁₁ N₃ O | POS | 1.1632212 | △△△ | 21.10507926 |
| 234 | Histamine | 1217 | C₅ H₉ N₃ | POS | 1.161779453 | △△△ | 18.335503 |
| 235 | Taurocholic acid | 7208 | C₂₆ H₄₅ N O₇ S | POS | 1.15186796 | △△△ | 0.061587602 |
| 236 | Adenosine | 4524 | C₁₀ H₁₃ N₅ O₄ | POS | 1.16477486 | △△△ | 2.071199598 |
| 237 | LPC 18:1-SN1 | 10178 | C₂₆ H₅₂ N O₇ P | POS | 1.165433874 | △△△ | 0.17544683 |
| 238 | LPC 22:6-SN1 | 9511 | C₃₀ H₅₀ N O₇ P | POS | 1.159563144 | △△△ | 0.039473303 |
| 239 | LPC 20:4-SN1 | 9573 | C₂₈ H₅₀ N O₇ P | POS | 1.164676563 | △△△ | 0.130635592 |
| 240 | PC O-20:4 | 9412 | C₂₈ H₅₀ N O₇ P | POS | 1.163924398 | △△△ | 0.055913645 |
| 241 | PC O-16:0 | 9762 | C₂₄ H₅₀ N O₇ P | POS | 1.164297838 | △△△ | 0.200611662 |
| 242 | PC O-18:1 | 9968 | C₂₆ H₅₂ N O₇ P | POS | 1.160776484 | △△△ | 0.154033556 |
| 243 | LPC 22:6-SN2 | 9353 | C₃₀ H₅₀ N O₇ P | POS | 1.165128366 | △△△ | 0.013369295 |
| 244 | Palmitoylcarnitine | 8827 | C₂₃ H₄₅ N O₄ | POS | 1.165644543 | △△△ | 0.207095532 |
| 245 | CAR 18:1 | 8983 | C₂₅ H₄₈ N O₄ | POS | 1.165620393 | △△△ | 0.424010495 |
| 246 | PC O-18:0 | 10527 | C₂₆ H₅₄ N O₇ P | POS | 1.164135467 | △△△ | 0.164295636 |
| 247 | N1,N1-dicyclohexyl-3-(1-phthyl)acrylamide | 6745 | C₂₅ H₃₁ N O | POS | 1.15743097 | △△△ | 230.8871619 |
| 248 | CAR 18:2 | 8597 | C₂₅ H₄₆ N O₄ | POS | 1.159323433 | △△△ | 0.366495623 |
| 249 | 6-Methylquinoline | 5548 | C₁₀ H₉ N | POS | 1.152228269 | △△△ | 3.77468933 |
| 250 | 5-[(10Z)-14-(3,5-dihydroxyphenyl)tetradec-10-en-1-yl]benzene-1,3-diol | 8369 | C₂₆ H₃₆ O₄ | POS | 1.163275066 | △△△ | 4.838249844 |
| 251 | CAR 20:4 | 8553 | C₂₇ H₄₆ N O₄ | POS | 1.155010559 | △△△ | 0.421742891 |
| 252 | 5,6-dimethoxy-2-(2-methoxyphenyl)-4H-chromen-4-one | 6013 | C₁₈ H₁₆ O₅ | POS | 1.165773849 | △△△ | 3.646298503 |
| 253 | PC O-18:2 | 9459 | C₂₆ H₅₀ N O₇ P | POS | 1.165682878 | △△△ | 0.160269431 |
| 254 | 16(R)-HETE | 8863 | C₂₀ H₃₂ O₃ | POS | 1.152735395 | △△△ | 0.2801875 |
| 255 | FMH | 5128 | C₂₀ H₂₇ N₅ O₄ S | POS | 1.163633048 | △△△ | 0.400331096 |
| 256 | 3-(2-Hydroxyethyl)indole | 6174 | C₁₀ H₁₁ N O | POS | 1.165587226 | △△△ | 6.907051434 |
| 257 | Andamide (AEA) | 9732 | C₂₂ H₃₇ N O₂ | POS | 1.161495239 | △△△ | 2.338685222 |
| 258 | Tetrahydrocortisone | 7102 | C₂₁ H₃₂ O₅ | POS | 1.164878869 | △△△ | 0.050570564 |
| 259 | 2-Methoxybenzaldehyde | 6012 | C₈ H₈ O₂ | POS | 1.165773849 | △△△ | 3.646298503 |
| 260 | 25-hydroxycholecalciferol | 7066 | C₂₇ H₄₄ O₂ | POS | 1.155613464 | △△△ | 0.489096623 |
| 261 | CAR 18:0 | 9414 | C₂₅ H₅₀ N O₄ | POS | 1.16527857 | △△△ | 0.184365145 |
| 262 | Valine | 1919 | C₅ H₁₁ N O₂ | POS | 1.018332548 | △△△ | 2.052360254 |
| 263 | LPC O-14:0 | 9686 | C₂₂ H₄₈ N O₆ P | POS | 1.16521035 | △△△ | 0.003487245 |
| 264 | N-Acetyl-D-lactosamine | 1437 | C₁₄ H₂₅ N O₁₁ | POS | 1.150187968 | △△△ | 2.250791437 |
| 265 | 3-amino-2-phenyl-2H-pyrazolo[4,3-c]pyridine-4,6-diol | 6286 | C₁₂ H₁₀ N₄ O₂ | POS | 1.165633138 | △△△ | 2.75606022 |
| 266 | Mycophenolic acid | 1425 | C₁₇ H₂₀ O₆ | POS | 1.163507264 | △△△ | 0.249281489 |
| 267 | LPC 17:0-SN1 | 10408 | C₂₅ H₅₂ N O₇ P | POS | 1.14154221 | △△△ | 0.162553281 |
| 268 | LPC 14:0-SN1 | 9174 | C₂₂ H₄₆ N O₇ P | POS | 1.164463734 | △△△ | 0.094550896 |
| 269 | CAR 12:0 | 7605 | C₁₉ H₃₈ N O₄ | POS | 1.165624913 | △△△ | 0.192961822 |
| 270 | LPC 15:0-SN1 | 9563 | C₂₃ H₄₈ N O₇ P | POS | 1.164785416 | △△△ | 0.147881613 |
| 271 | CAR 22:6 | 8519 | C₂₉ H₄₆ N O₄ | POS | 1.160531363 | △△△ | 0.036823754 |
| 272 | R-1 Methandamide phosphate | 569 | C₂₃ H₄₀ N O₅ P | POS | 1.150535805 | △△△ | 2.635971368 |
| 273 | Cyclopentyl fentanyl-d5 | 7544 | C₂₅ H₂₇ [₂]H₅ N₂ O | POS | 1.035519984 | △△△ | 0.280445127 |
| 274 | U-44069 | 9497 | C₂₁ H₃₄ O₄ | POS | 1.054893363 | △△△ | 0.17365248 |
| 275 | PC O-16:1 | 9203 | C₂₄ H₄₈ N O₇ P | POS | 1.154151219 | △△△ | 0.086094161 |
| 276 | LPC 20:1-SN1 | 11016 | C₂₈ H₅₆ N O₇ P | POS | 1.164964374 | △△△ | 0.119022934 |
| 277 | 22(S)-Hydroxycholesterol | 966 | C₂₇ H₄₆ O₂ | POS | 1.122005427 | △△△ | 2.162233885 |
| 278 | CAR 16:3 | 7835 | C₂₃ H₄₀ N O₄ | POS | 1.146146455 | △△△ | 0.328064505 |
| 279 | 9-Oxo-10(E),12(E)-octadecadienoic acid | 8868 | C₁₈ H₃₀ O₃ | POS | 1.050844286 | △△△ | 97.51205054 |
| 280 | tert-Butyl N-[1-(aminocarbonyl)-3-methylbutyl]carbamate | 5961 | C₁₁ H₂₂ N₂ O₃ | POS | 1.16041076 | △△△ | 0.307802628 |
| 281 | Prostaglandin K2 | 8276 | C₂₀ H₃₀ O₅ | POS | 1.165514551 | △△△ | 0.086129216 |
| 282 | 6-(Dimethylamino)purine | 5197 | C₇ H₉ N₅ | POS | 1.161467022 | △△△ | 3.164053443 |
| 283 | 2-Arachidonoyl glycerol | 9819 | C₂₃ H₃₈ O₄ | POS | 1.161555451 | △△△ | 0.220000805 |
| 284 | VLK | 5391 | C₁₇ H₃₄ N₄ O₄ | POS | 1.165397301 | △△△ | 0.129892307 |
| 285 | Lysopc 18:2 | 10508 | C₂₆ H₅₀ N O₇ P | POS | 1.164410035 | △△△ | 0.152309298 |
| 286 | LPC 20:3-SN1 | 9934 | C₂₈ H₅₂ N O₇ P | POS | 1.162580911 | △△△ | 0.116727597 |
| 287 | LysoPC 20:2 | 10437 | C₂₈ H₅₄ N O₇ P | POS | 1.163226416 | △△△ | 0.147016788 |
| 288 | N-(2,4-Dimethylphenyl)formamide | 5155 | C₉ H₁₁ N O | POS | 1.163992693 | △△△ | 0.215142436 |
| 289 | LPC 22:4-SN1 | 10252 | C₃₀ H₅₄ N O₇ P | POS | 1.129768598 | △△△ | 0.158164491 |
| 290 | Hydroxyprogesterone caproate | 7089 | C₂₇ H₄₀ O₄ | POS | 1.020347702 | △△△ | 0.344000638 |
| 291 | Glycerol 1-hexadecanoate | 10197 | C₁₉ H₃₈ O₄ | POS | 1.163987789 | △△△ | 0.348597941 |
| 292 | Kahweol | 6168 | C₂₀ H₂₆ O₃ | POS | 1.164771737 | △△△ | 0.159628976 |
| 293 | CAR 20:1 | 9541 | C₂₇ H₅₂ N O₄ | POS | 1.160471469 | △△△ | 0.430241567 |
| 294 | 4-[4-(aminocarbonyl)piperidino]-4-oxobut-2-enoic acid | 5286 | C₁₀ H₁₄ N₂ O₄ | POS | 1.164604425 | △△△ | 3.784008843 |
| 295 | Canbidiolic acid | 7603 | C₂₂ H₃₀ O₄ | POS | 1.157459598 | △△△ | 0.085635177 |
| 296 | LLK | 5569 | C₁₈ H₃₆ N₄ O₄ | POS | 1.164955147 | △△△ | 0.090814978 |
| 297 | 1,3-Dihydro-1,3,3-trimethyl-2H-indol-2-ylidene acetaldehyde | 5828 | C₁₃ H₁₅ N O | POS | 1.162922494 | △△△ | 0.295267388 |
| 298 | L-threo-3-Phenylserine | 1536 | C₉ H₁₁ N O₃ | POS | 1.058731365 | △△△ | 0.328171641 |
| 299 | N1-(4-methylidene-3-thia-1-azaspiro[45]dec-2-yliden)-2-chloroaniline | 1322 | C₁₅ H₁₇ Cl N₂ S | POS | 1.142468048 | △△△ | 0.111405674 |
| 300 | INK | 5184 | C₁₆ H₃₁ N₅ O₅ | POS | 1.163315641 | △△△ | 0.429374372 |
| 301 | CAR 20:5 | 824 | C₂₇ H₄₄ N O₄ | POS | 1.125223936 | △△△ | 0.361373045 |
| 302 | 5-trans prostaglandin F2β | 7369 | C₂₀ H₃₄ O₅ | POS | 1.164793122 | △△△ | 0.145179105 |
| 303 | 15-Deoxy-_12,14-prostaglandin J2-2-glycerol ester | 8196 | C₂₃ H₃₄ O₅ | POS | 1.045341134 | △△△ | 0.174027582 |
| 304 | Ala-Val | 3411 | C₈ H₁₆ N₂ O₃ | POS | 1.162756735 | △△△ | 0.162076604 |
| 305 | Muramic acid | 1591 | C₉ H₁₇ N O₇ | POS | 1.097551171 | △△△ | 0.445126854 |
| 306 | KLK | 8216 | C₁₈ H₃₇ N₅ O₄ | POS | 1.162604364 | △△△ | 0.047874223 |
| 307 | (S)-AL 8810 | 6386 | C₂₄ H₃₁ F O₄ | POS | 1.149636901 | △△△ | 0.051081043 |
| 308 | N-[(4-hydroxy-3-methoxyphenyl)methyl]-8-methylnomide | 7595 | C₁₈ H₂₉ N O₃ | POS | 1.165612718 | △△△ | 6.042404847 |
| 309 | 5α-Dihydrotestosterone | 8282 | C₁₉ H₃₀ O₂ | POS | 1.158696116 | △△△ | 0.331311291 |
| 310 | 15(R)-Prostaglandin D2 | 7058 | C₂₀ H₃₂ O₅ | POS | 1.165890308 | △△△ | 0.246259505 |
| 311 | Isopropyl myristate | 8293 | C₁₇ H₃₄ O₂ | POS | 1.155216325 | △△△ | 2.744676834 |
| 312 | Tropine | 5414 | C₈ H₁₅ N O | POS | 1.156267512 | △△△ | 2.463728675 |
| 313 | Arachidonoyl amide | 7813 | C₂₀ H₃₃ N O | POS | 1.146388402 | △△△ | 0.081819867 |
| 314 | Glu-Val-Phe | 5649 | C₁₉ H₂₇ N₃ O₆ | POS | 1.116891195 | △△△ | 0.408239154 |
| 315 | Palmitoleic Acid | 10181 | C₁₆ H₃₀ O₂ | POS | 1.15586569 | △△△ | 0.366441301 |
| 316 | Octadecamine | 9317 | C₁₈ H₃₉ N | POS | 1.164193879 | △△△ | 0.145141567 |
| 317 | Tetrahydroaldosterone | 6619 | C₂₁ H₃₂ O₅ | POS | 1.071804607 | △△△ | 0.378585064 |
| 318 | 11-Deoxy prostaglandin F1α | 8801 | C₂₀ H₃₆ O₄ | POS | 1.143983666 | △△△ | 0.20614972 |
| 319 | Deoxyinosine | 5091 | C₁₀ H₁₂ N₄ O₄ | POS | 1.151984731 | △△△ | 3.944242728 |
| 320 | 2,4-dihydroxyheptadec-16-en-1-yl acetate | 7655 | C₁₉ H₃₆ O₄ | POS | 1.162752287 | △△△ | 5.427509451 |
| 321 | PC 18:0_18:1 | 837 | C₄₄ H₈₆ N O₈ P | POS | 1.15661671 | △△△ | 37.48508427 |
| 322 | CAR 16:1 | 8637 | C₂₃ H₄₄ N O₄ | POS | 1.153661436 | △△△ | 0.283237995 |
| 323 | 11-Deoxy prostaglandin F1β | 8491 | C₂₀ H₃₆ O₄ | POS | 1.141160384 | △△△ | 0.318302398 |
| 324 | 4-Hexyloxyaniline | 7204 | C₁₂ H₁₉ N O | POS | 1.165420555 | △△△ | 0.047717893 |
| 325 | TNK | 5822 | C₁₄ H₂₇ N₅ O₆ | POS | 1.160042693 | △△△ | 0.49445559 |
| 326 | 2-(14,15-Epoxyeicosatrienoyl) glycerol | 939 | C₂₃ H₃₈ O₅ | POS | 1.164952674 | △△△ | 0.102565201 |
| 327 | Andrographolide | 6843 | C₂₀ H₃₀ O₅ | POS | 1.154041609 | △△△ | 0.326594905 |
| 328 | PC O-17:1 | 9583 | C₂₅ H₅₀ N O₇ P | POS | 1.146279833 | △△△ | 0.255438558 |
| 329 | 1-(3,4-dimethoxyphenyl)ethan-1-one oxime | 4608 | C₁₀ H₁₃ N O₃ | POS | 1.163989783 | △△△ | 0.417160186 |
| 330 | FRH | 10006 | C₂₁ H₃₀ N₈ O₄ | POS | 1.163635314 | △△△ | 0.052079371 |
| 331 | 2-hydroxy-6-[(8Z,11Z)-pentadeca-8,11,14-trien-1-yl]benzoic acid | 1052 | C₂₂ H₃₀ O₃ | POS | 1.1309038 | △△△ | 2.593899571 |
| 332 | PC 18:0_18:0 | 9666 | C₄₄ H₈₈ N O₈ P | POS | 1.155328517 | △△△ | 23.72654767 |
| 333 | 6 β-Hydroxycortisol | 5855 | C₂₁ H₃₀ O₆ | POS | 1.155957467 | △△△ | 0.083922832 |
| 334 | 2-[5-(2-hydroxypropyl)oxolan-2-yl]propanoic acid | 5835 | C₁₀ H₁₈ O₄ | POS | 1.157447399 | △△△ | 3.182576679 |
| 335 | ethyl 2-{[(1-methyl-3-propyl-1H-pyrazol-4-yl)carbonyl]amino}acetate | 5619 | C₁₂ H₁₉ N₃ O₃ | POS | 1.157096521 | △△△ | 0.361072956 |
| 336 | 13,14-dihydro Prostaglandin E1 | 7788 | C₂₀ H₃₆ O₅ | POS | 1.154492674 | △△△ | 0.275624249 |
| 337 | ALK | 5105 | C₁₅ H₃₀ N₄ O₄ | POS | 1.104895657 | △△△ | 0.181603077 |
| 338 | LPE 17:0 | 10239 | C₂₂ H₄₆ N O₇ P | POS | 1.078237879 | △△△ | 0.274292102 |
| 339 | Trenbolone acetate | 6685 | C₂₀ H₂₄ O₃ | POS | 1.124899369 | △△△ | 0.314828533 |
| 340 | CAR 17:1 | 866 | C₂₄ H₄₆ N O₄ | POS | 1.038711968 | △△△ | 0.45453839 |
| 341 | (±)-Abscisic acid | 1503 | C₁₅ H₂₀ O₄ | POS | 1.077157682 | △△△ | 0.332261727 |
| 342 | Caffeine | 1374 | C₈ H₁₀ N₄ O₂ | POS | 1.023098105 | △△△ | 0.054256248 |
| 343 | LPC 16:0-SN1 | 10523 | C₂₄ H₅₀ N O₇ P | POS | 1.165562725 | △△△ | 0.109234491 |
| 344 | PC O-20:5 | 899 | C₂₈ H₄₈ N O₇ P | POS | 1.165569268 | △△△ | 0.057229328 |
| 345 | PC O-20:1 | 10749 | C₂₈ H₅₆ N O₇ P | POS | 1.160488686 | △△△ | 0.182573699 |
| 346 | PC O-20:2 | 10238 | C₂₈ H₅₄ N O₇ P | POS | 1.156964695 | △△△ | 0.057052379 |
| 347 | LPC 18:0-SN1 | 11312 | C₂₆ H₅₄ N O₇ P | POS | 1.165503324 | △△△ | 0.068494275 |
| 348 | LPC 20:5-SN1 | 915 | C₂₈ H₄₈ N O₇ P | POS | 1.163420045 | △△△ | 0.03591499 |
| 349 | Oxohongdefil | 5746 | C₂₅ H₃₂ N₆ O₄ | POS | 1.145869303 | △△△ | 3.984924848 |
| 350 | Guanethidine Monosulfate | 7799 | C₁₀ H₂₄ N₄ O₄ S | POS | 1.162526944 | △△△ | 0.081930192 |
| 351 | LPC 22:5-SN1 | 9956 | C₃₀ H₅₂ N O₇ P | POS | 1.109174693 | △△△ | 0.424482329 |
| 352 | 1,4-dihydroxy-1,4-dimethyl-7-(propan-2-ylidene)-decahydroazulen-6-one | 8675 | C₁₅ H₂₄ O₃ | POS | 1.160805571 | △△△ | 0.169061099 |
| 353 | Pleuromutilin | 7974 | C₂₂ H₃₄ O₅ | POS | 1.10269374 | △△△ | 0.140988484 |
| 354 | SPH | 5159 | C₁₄ H₂₁ N₅ O₅ | POS | 1.097030198 | △△△ | 0.260318298 |
| 355 | SNH | 2362 | C₁₃ H₂₀ N₆ O₆ | POS | 1.163489402 | △△△ | 0.205799752 |
| 356 | Capryloylglycine | 4912 | C₁₀ H₁₉ N O₃ | POS | 1.161961202 | △△△ | 2.069808535 |
| 357 | 1-methyl-3-phenyl-1H-pyrazol-5-amine | 8353 | C₁₀ H₁₁ N₃ | POS | 1.158184857 | △△△ | 3.26564966 |
| 358 | 3-Acetyl-11-keto-β-boswellic acid | 9633 | C₃₂ H₄₈ O₅ | POS | 1.158128939 | △△△ | 0.433814743 |
| 359 | Dextrorphan | 10684 | C₁₇ H₂₃ N O | POS | 1.162010476 | △△△ | 48.07914426 |
| 360 | LPC 17:1-SN1 | 9803 | C₂₅ H₅₀ N O₇ P | POS | 1.148518511 | △△△ | 0.172437985 |
| 361 | KNH | 514 | C₁₆ H₂₇ N₇ O₅ | POS | 1.158325594 | △△△ | 0.484197992 |
| 362 | HLK | 5168 | C₁₈ H₃₂ N₆ O₄ | POS | 1.153449827 | △△△ | 0.410989516 |
| 363 | CAR 14:2 | 7863 | C₂₁ H₃₈ N O₄ | POS | 1.164102665 | △△△ | 0.163609162 |
| 364 | LPH | 5676 | C₁₇ H₂₇ N₅ O₄ | POS | 1.157398482 | △△△ | 0.274677796 |
| 365 | RNK | 5777 | C₁₆ H₃₂ N₈ O₅ | POS | 1.152331117 | △△△ | 6.222531116 |
| 366 | PPK | 239 | C₁₆ H₂₈ N₄ O₄ | POS | 1.156687653 | △△△ | 0.455351191 |
| 367 | QNK | 551 | C₁₅ H₂₈ N₆ O₆ | POS | 1.032468274 | △△△ | 0.433903527 |
| 368 | LPC 17:0 | 10294 | C₂₅ H₅₂ N O₇ P | POS | 1.025770278 | △△△ | 0.176705644 |
| 369 | dAMP | 3873 | C₁₀ H₁₄ N₅ O₆ P | POS | 1.164554366 | △△△ | 2.205766815 |
| 370 | rasin | 6534 | C₄₃ H₇₂ O₁₁ | POS | 1.161099628 | △△△ | 2.937852114 |
| 371 | 4-methyl-6-[(4-methylphenyl)thio]-2-(2-pyridyl)pyrimidine | 2419 | C₁₇ H₁₅ N₃ S | POS | 1.151652086 | △△△ | 0.184479212 |
| 372 | Stercobilin | 7046 | C₃₃ H₄₆ N₄ O₆ | POS | 1.133782071 | △△△ | 0.198311802 |
| 373 | (2R,3S,4S,5R,6R)-2-(hydroxymethyl)-6-(propan-2-yloxy)oxane-3,4,5-triol | 5199 | C₉ H₁₈ O₆ | POS | 1.143242758 | △△△ | 0.22328596 |
| 374 | nor-6α-_xycodol | 3957 | C₁₇ H₂₁ N O₄ | POS | 1.16543817 | △△△ | 0.417513907 |
| 375 | ELK | 2876 | C₁₇ H₃₂ N₄ O₆ | POS | 1.163850745 | △△△ | 0.492469932 |
| 376 | PPH | 1961 | C₁₆ H₂₃ N₅ O₄ | POS | 1.163917356 | △△△ | 0.190285132 |
| 377 | Piperine | 1585 | C₁₇ H₁₉ N O₃ | POS | 1.162753719 | △△△ | 0.177982155 |
| 378 | 16-Heptadecyne-1,2,4-triol | 8564 | C₁₇ H₃₂ O₃ | POS | 1.062524545 | △△△ | 0.456835807 |
| 379 | LPC O-16:1 | 10349 | C₂₄ H₅₀ N O₆ P | POS | 1.161996326 | △△△ | 0.088134233 |
| 380 | LPE 20:4 | 9499 | C₂₅ H₄₄ N O₇ P | POS | 1.164830486 | △△△ | 0.217235578 |
| 381 | N6-Succinyl Adenosine | 5333 | C₁₄ H₁₇ N₅ O₈ | POS | 1.165553798 | △△△ | 4.884722001 |
| 382 | LPC O-18:1 | 10695 | C₂₆ H₅₄ N O₆ P | POS | 1.165792379 | △△△ | 0.07446631 |
| 383 | Thr-Leu | 5474 | C₁₀ H₂₀ N₂ O₄ | POS | 1.140362018 | △△△ | 0.253730956 |
| 384 | Sphingosine (d18:1) | 807 | C₁₈ H₃₇ N O₂ | POS | 1.16169276 | △△△ | 0.408796044 |
| 385 | Feruloyl Putrescine | 5682 | C₁₄ H₂₀ N₂ O₃ | POS | 1.165682352 | △△△ | 0.19302638 |
| 386 | D-Ala-D-Ala | 1494 | C₆ H₁₂ N₂ O₃ | POS | 1.114153015 | △△△ | 0.356219436 |
| 387 | Docosahexaenoyl Ethanolamide | 9679 | C₂₄ H₃₇ N O₂ | POS | 1.165011584 | △△△ | 0.332929905 |
| 388 | Gly-Phe | 5555 | C₁₁ H₁₄ N₂ O₃ | POS | 1.163730594 | △△△ | 0.470127236 |
| 389 | L-Leucyl-L-Alanine | 5151 | C₉ H₁₈ N₂ O₃ | POS | 1.165265101 | △△△ | 0.159173257 |
| 390 | LPC O-15:0 | 10096 | C₂₃ H₅₀ N O₆ P | POS | 1.15891468 | △△△ | 0.126553502 |
| 391 | LPC O-15:1 | 9924 | C₂₃ H₄₈ N O₆ P | POS | 1.157180036 | △△△ | 0.217471518 |
| 392 | cis-7-Hexadecenoic Acid | 8862 | C₁₆ H₃₀ O₂ | POS | 1.165264295 | △△△ | 6.619668489 |
| 393 | LPE 16:1 | 9287 | C₂₁ H₄₂ N O₇ P | POS | 1.164580321 | △△△ | 0.133266185 |
| 394 | LPC O-18:3 | 10013 | C₂₆ H₅₀ N O₆ P | POS | 1.161554023 | △△△ | 0.083965421 |
| 395 | LPC O-18:0 | 11368 | C₂₆ H₅₆ N O₆ P | POS | 1.164160999 | △△△ | 0.071956221 |
| 396 | 12(S)-HETE | 8104 | C₂₀ H₃₂ O₃ | POS | 1.156101092 | △△△ | 0.24322431 |
| 397 | N6-Methyladenine | 5278 | C₆ H₇ N₅ | POS | 1.163840226 | △△△ | 4.220536957 |
| 398 | Lithocholic acid | 10355 | C₂₄ H₄₀ O₃ | POS | 1.150867794 | △△△ | 0.418991748 |
| 399 | JNJ-1661010 | 1459 | C₁₉ H₁₉ N₅ O S | POS | 1.138495081 | △△△ | 2.160767791 |
| 400 | Phe-Phe | 5898 | C₁₈ H₂₀ N₂ O₃ | POS | 1.166078297 | △△△ | 0.134597183 |
| 401 | LPE 22:5 | 9696 | C₂₇ H₄₆ N O₇ P | POS | 1.122825076 | △△△ | 0.265383502 |
| 402 | Methyl EudesMate | 593 | C₁₁ H₁₄ O₅ | POS | 1.160897005 | △△△ | 0.261979806 |
| 403 | Ne-(1-Carboxymethyl)-L-lysine | 2369 | C₈ H₁₆ N₂ O₄ | POS | 1.160756915 | △△△ | 0.149856743 |
| 404 | Desoxycortone | 7332 | C₂₁ H₃₀ O₃ | POS | 1.122529026 | △△△ | 0.36925791 |
| 405 | O-Phospho-L-serine | 1337 | C₃ H₈ N O₆ P | POS | 1.117521668 | △△△ | 0.431457163 |
| 406 | N-Acetyl-aspartic acid | 1571 | C₆ H₉ N O₅ | POS | 1.045088684 | △△△ | 0.156268961 |
| 407 | Isophorone | 6944 | C₉ H₁₄ O | POS | 1.023009988 | △△△ | 0.300329108 |
| 408 | Sodium cholate | 6985 | C₂₄ H₃₉ O₅ | POS | 1.151140413 | △△△ | 5.143912788 |
| 409 | LPC 20:2 | 10484 | C₂₈ H₅₄ N O₇ P | POS | 1.162254106 | △△△ | 0.205774189 |
| 410 | 17α-Hydroxyprogesterone | 5956 | C₂₁ H₃₀ O₃ | POS | 1.153241894 | △△△ | 0.147738974 |
| 411 | 2-Deoxyuridine | 4015 | C₉ H₁₂ N₂ O₅ | POS | 1.154930695 | △△△ | 4.666352927 |
| 412 | MAG (18:2) | 8552 | C₂₁ H₃₈ O₄ | POS | 1.010888707 | △△△ | 0.173249768 |
| 413 | Ala-Gln | 1466 | C₈ H₁₅ N₃ O₄ | POS | 1.148560254 | △△△ | 0.376281631 |
| 414 | LPC O-17:0 | 10957 | C₂₅ H₅₄ N O₆ P | POS | 1.154512154 | △△△ | 0.155058852 |
| 415 | S-Adenosyl-L-methionine | 6099 | C₁₅ H₂₂ N₆ O₅ S | POS | 1.149042587 | △△△ | 0.481728711 |
| 416 | L-beta-Imidazolelactic acid | 2625 | C₆ H₈ N₂ O₃ | POS | 1.159787859 | △△△ | 0.464506803 |

^△^*P*<0.05，^△△^*P*<0.01，^△△△^*P*<0.001 vs. the CON group

**Supplementary Table 4 Differential metabolites between the H-SCFs and CUMS groups**

| **NO** | **Metabolite** | **t_R_(min)** | **Formula** | **Ionization mode** | **VIP** | **P** | **Fold** |
| --- | --- | --- | --- | --- | --- | --- | --- |
|  |  |  |  |  | **score** | **value** | **change** |
| 1 | Gluconic acid | 1366 | C₆ H₁₂ O₇ | NEG | 1.170013472 | *** | 3.728770723 |
| 2 | 2-Hydroxycaproic acid | 6075 | C₆ H₁₂ O₃ | NEG | 1.168234374 | *** | 0.476764189 |
| 3 | β-Muricholic acid | 7374 | C₂₄ H₄₀ O₅ | NEG | 1.172453762 | *** | 3.578535067 |
| 4 | Phenylacetaldehyde | 5974 | C₈ H₈ O | NEG | 1.136561614 | *** | 3.949182223 |
| 5 | (±)11(12)-EET | 8799 | C₂₀ H₃₂ O₃ | NEG | 1.130667851 | *** | 0.422951566 |
| 6 | (±)11-HETE | 863 | C₂₀ H₃₂ O₃ | NEG | 1.169786261 | *** | 0.371006567 |
| 7 | Sucrose | 1424 | C₁₂ H₂₂ O₁₁ | NEG | 1.169442169 | *** | 3.360134783 |
| 8 | β-D-Glucopyranuronic acid | 1371 | C₆ H₁₀ O₇ | NEG | 1.170968178 | *** | 3.217107634 |
| 9 | 2'-Deoxyinosine | 5095 | C₁₀ H₁₂ N₄ O₄ | NEG | 1.172189499 | *** | 3.333726211 |
| 10 | 8(S)-Hydroxy-(5Z,9E,11Z,14Z)-eicosatetraenoic acid | 9028 | C₂₀ H₃₂ O₃ | NEG | 1.156697113 | *** | 0.278986759 |
| 11 | ent-Prostaglandin F2α | 7055 | C₂₀ H₃₄ O₅ | NEG | 1.169474052 | *** | 0.44279099 |
| 12 | 15(S)-HpETE | 8127 | C₂₀ H₃₂ O₄ | NEG | 1.144722359 | *** | 0.47088845 |
| 13 | 20-Hydroxy-(5Z,8Z,11Z,14Z)-eicosatetraenoic acid | 9721 | C₂₀ H₃₂ O₃ | NEG | 1.15153572 | *** | 0.293310895 |
| 14 | Monobenzyl phthalate | 6163 | C₁₅ H₁₂ O₄ | NEG | 1.17321971 | *** | 4.823586951 |
| 15 | (±)10(11)-EpDPA | 9139 | C₂₂ H₃₂ O₃ | NEG | 1.168157146 | *** | 0.297311751 |
| 16 | Ascorbic acid | 1425 | C₆ H₈ O₆ | NEG | 1.140178974 | *** | 3.390418122 |
| 17 | Adipic acid | 554 | C₆ H₁₀ O₄ | NEG | 1.15577988 | *** | 2.318347049 |
| 18 | Glycodeoxycholic acid | 81 | C₂₆ H₄₃ N O₅ | NEG | 1.096723179 | *** | 2.14566455 |
| 19 | 2-Isopropylmalic acid | 5793 | C₇ H₁₂ O₅ | NEG | 1.167586946 | *** | 2.254691223 |
| 20 | 3-Hydroxy-3-methylglutaric acid | 3417 | C₆ H₁₀ O₅ | NEG | 1.099645449 | *** | 2.464348866 |
| 21 | Glycocholic acid | 6816 | C₂₆ H₄₃ N O₆ | NEG | 1.167286383 | *** | 8.917803483 |
| 22 | Xylitol | 1412 | C₅ H₁₂ O₅ | NEG | 1.077459403 | *** | 0.347620759 |
| 23 | 8(S),15(S)-DiHETE | 867 | C₂₀ H₃₂ O₄ | NEG | 1.168683744 | *** | 0.20866493 |
| 24 | 3,5-Dihydroxybenzoic acid | 5393 | C₇ H₆ O₄ | NEG | 1.169513666 | *** | 2.725336229 |
| 25 | Thymidine 5'-monophosphate | 295 | C₁₀ H₁₅ N₂ O₈ P | NEG | 1.168856991 | *** | 0.211828124 |
| 26 | 5-Aminovaleric acid | 1903 | C₅ H₁₁ N O₂ | NEG | 1.14054469 | *** | 2.000750034 |
| 27 | Valeric acid | 5522 | C₅ H₁₀ O₂ | NEG | 1.072611219 | *** | 2.023849257 |
| 28 | Guanosine monophosphate (GMP) | 1885 | C₁₀ H₁₄ N₅ O₈ P | NEG | 1.067686698 | *** | 0.489456292 |
| 29 | Docosahexaenoic acid | 10303 | C₂₂ H₃₂ O₂ | NEG | 1.171131823 | *** | 0.13957783 |
| 30 | Taurochenodeoxycholic acid | 7105 | C₂₆ H₄₅ N O₆ S | NEG | 1.169627787 | *** | 0.42661746 |
| 31 | D-Raffinose | 1473 | C₁₈ H₃₂ O₁₆ | NEG | 1.164879963 | *** | 4.839427748 |
| 32 | Adrenic acid | 11044 | C₂₂ H₃₆ O₂ | NEG | 1.169245835 | *** | 0.077586712 |
| 33 | Taurine | 1837 | C₂ H₇ N O₃ S | NEG | 1.16417056 | *** | 0.474159822 |
| 34 | Uric acid | 1995 | C₅ H₄ N₄ O₃ | NEG | 1.170074249 | *** | 0.349775593 |
| 35 | Adenosine 5'-monophosphate | 1813 | C₁₀ H₁₄ N₅ O₇ P | NEG | 1.150997552 | *** | 0.313415843 |
| 36 | Guanosine | 4861 | C₁₀ H₁₃ N₅ O₅ | NEG | 1.15493014 | *** | 2.066318601 |
| 37 | Uridine | 2649 | C₉ H₁₂ N₂ O₆ | NEG | 1.091761785 | *** | 6.768374842 |
| 38 | 5-Hydroxyindole-3-acetic acid | 5849 | C₁₀ H₉ N O₃ | NEG | 1.172278302 | *** | 2.408646007 |
| 39 | N-Acetyl-L-methionine | 5623 | C₇ H₁₃ N O₃ S | NEG | 1.169093461 | *** | 0.457237697 |
| 40 | Elaidic acid | 11032 | C₁₈ H₃₄ O₂ | NEG | 1.162855746 | *** | 0.487504689 |
| 41 | ST 24:1;O4;T | 7368 | C₂₆ H₄₅ N O₇ S | NEG | 1.16147544 | *** | 0.452485756 |
| 42 | 3,8,9-trihydroxy-10-propyl-3,4,5,8,9,10-hexahydro-2H-oxecin-2-one | 5914 | C₁₂ H₂₀ O₅ | NEG | 1.168116334 | *** | 2.461288955 |
| 43 | LPE O-16:1 | 10167 | C₂₁ H₄₄ N O₆ P | NEG | 1.174443426 | *** | 0.203001299 |
| 44 | 8Z,11Z,14Z-Eicosatrienoic acid | 10875 | C₂₀ H₃₄ O₂ | NEG | 1.167679851 | *** | 0.058802477 |
| 45 | LPE O-18:2 | 10348 | C₂₃ H₄₆ N O₆ P | NEG | 1.162501618 | *** | 0.198976019 |
| 46 | LPC 22:6 | 9477 | C₃₀ H₅₀ N O₇ P | NEG | 1.171536756 | *** | 0.278677169 |
| 47 | LPC 18:0 | 10752 | C₂₆ H₅₄ N O₇ P | NEG | 1.172721921 | *** | 0.262352258 |
| 48 | D-Glucose 6-phosphate | 1237 | C₆ H₁₃ O₉ P | NEG | 1.100919518 | *** | 0.043639039 |
| 49 | LPI 20:4 | 10331 | C₂₉ H₄₉ O₁₂ P | NEG | 1.173790603 | *** | 0.106124394 |
| 50 | LPE O-18:3 | 9861 | C₂₃ H₄₄ N O₆ P | NEG | 1.174195841 | *** | 0.0653045 |
| 51 | (±)8-HEPE | 8589 | C₂₀ H₃₀ O₃ | NEG | 1.084476526 | *** | 0.48191871 |
| 52 | (+/-)5(6)-DiHET | 8601 | C₂₀ H₃₄ O₄ | NEG | 1.171429108 | *** | 0.12074937 |
| 53 | (+/-)13-HODE | 9408 | C₁₈ H₃₂ O₃ | NEG | 1.170605389 | *** | 0.384808983 |
| 54 | 1a,1b-Dihomo prostaglandin E1 | 8744 | C₂₂ H₃₈ O₅ | NEG | 1.147262598 | *** | 0.378041821 |
| 55 | Lactobionic acid | 1432 | C₁₂ H₂₂ O₁₂ | NEG | 1.171951487 | *** | 4.660024836 |
| 56 | (3-Methoxy-4-hydroxyphenyl)ethylene glycol sulfate | 5617 | C₉ H₁₂ O₇ S | NEG | 1.138577671 | *** | 2.228497137 |
| 57 | FAHFA 2:0/18:1 | 8935 | C₂₀ H₃₆ O₄ | NEG | 1.146197146 | *** | 0.312419059 |
| 58 | LPS 18:1 | 10512 | C₂₄ H₄₆ N O₉ P | NEG | 1.052830021 | *** | 0.081420884 |
| 59 | 3-(2-thienyl)-1,2,4-oxadiazole-5-carbohydrazide | 1479 | C₇ H₆ N₄ O₂ S | NEG | 1.169619582 | *** | 3.67334019 |
| 60 | LPS 15:0 | 9602 | C₂₁ H₄₂ N O₉ P | NEG | 1.164407019 | *** | 0.408452991 |
| 61 | LPG 16:1 | 10262 | C₂₂ H₄₃ O₉ P | NEG | 1.007145394 | *** | 0.167409243 |
| 62 | NSI-189 | 6552 | C₂₂ H₃₀ N₄ O | NEG | 1.146767576 | *** | 0.348553957 |
| 63 | Acardic acid | 9728 | C₂₂ H₃₆ O₃ | NEG | 1.17121039 | *** | 0.18123885 |
| 64 | (±)11(12)-DiHET | 816 | C₂₀ H₃₄ O₄ | NEG | 1.166955792 | *** | 0.395940347 |
| 65 | PG 4:0_14:0 | 11035 | C₂₄ H₄₇ O₁₀ P | NEG | 1.173720689 | *** | 0.162650868 |
| 66 | Cer 18:2;2O/18:0 | 10367 | C₃₆ H₆₉ N O₃ | NEG | 1.167508761 | *** | 0.366712644 |
| 67 | Aldosterone | 6703 | C₂₁ H₂₈ O₅ | NEG | 1.161373177 | *** | 0.301137245 |
| 68 | ST 24:2;O4 | 892 | C₂₄ H₃₈ O₄ | NEG | 1.074558092 | *** | 0.335088194 |
| 69 | 3-Methylglutaric acid | 4204 | C₆ H₁₀ O₄ | NEG | 1.170963812 | *** | 0.465853354 |
| 70 | Lysopc 17:0 | 10752 | C₂₅ H₅₂ N O₇ P | NEG | 1.171414445 | *** | 0.261393754 |
| 71 | 4-Hydroxybenzoic acid | 5673 | C₇ H₆ O₃ | NEG | 1.131091446 | *** | 2.175496628 |
| 72 | 4-Toluenesulfonic acid | 5618 | C₇ H₈ O₃ S | NEG | 1.171087497 | *** | 9.039083189 |
| 73 | LPE O-18:1 | 10466 | C₂₃ H₄₈ N O₆ P | NEG | 1.006958659 | *** | 0.258014882 |
| 74 | LPE O-15:1 | 9769 | C₂₀ H₄₂ N O₆ P | NEG | 1.169642279 | *** | 0.246121401 |
| 75 | FAHFA 22:6/3:0 | 10387 | C₂₅ H₃₆ O₄ | NEG | 1.168327286 | *** | 0.345880267 |
| 76 | CerP 15:0;2O/10:0 | 786 | C₂₅ H₅₂ N O₆ P | NEG | 1.039279144 | *** | 3.57035063 |
| 77 | Hexadecanedioic acid | 7009 | C₁₆ H₃₀ O₄ | NEG | 1.157759398 | *** | 2.694605356 |
| 78 | Ursolic acid | 10117 | C₃₀ H₄₈ O₃ | NEG | 1.167490681 | *** | 2.906830544 |
| 79 | LPC 20:3 | 99 | C₂₈ H₅₂ N O₇ P | NEG | 1.173065146 | *** | 0.052995707 |
| 80 | LPG 18:2 | 10835 | C₂₄ H₄₅ O₉ P | NEG | 1.170209171 | *** | 0.17373508 |
| 81 | 16-Hydroxyhexadecanoic acid | 9993 | C₁₆ H₃₂ O₃ | NEG | 1.173986909 | *** | 0.234435974 |
| 82 | Thromboxane B3 | 7584 | C₂₀ H₃₂ O₆ | NEG | 1.12122257 | *** | 0.466446043 |
| 83 | 3-Phosphoglyceric acid | 1398 | C₃ H₇ O₇ P | NEG | 1.16642614 | *** | 2.761091272 |
| 84 | Stachyose | 1482 | C₂₄ H₄₂ O₂₁ | NEG | 1.171019444 | *** | 4.799115642 |
| 85 | LPE O-18:0 | 1109 | C₂₃ H₅₀ N O₆ P | NEG | 1.160600639 | *** | 0.494907415 |
| 86 | Ethyl chrysanthemumate | 6297 | C₁₂ H₂₀ O₂ | NEG | 1.127361727 | *** | 2.482864952 |
| 87 | Esculin | 5339 | C₁₅ H₁₆ O₉ | NEG | 1.098748991 | *** | 2.234747377 |
| 88 | N1-[3-(trifluoromethyl)phenyl]-4-chlorobenzamide | 141 | C₁₄ H₉ Cl F₃ N O | NEG | 1.16642614 | *** | 2.761091272 |
| 89 | PE O-16:1_22:4 | 8705 | C₄₃ H₇₈ N O₇ P | NEG | 1.090324357 | *** | 2.717740358 |
| 90 | Lauric acid ethyl ester | 8168 | C₁₄ H₂₈ O₂ | NEG | 1.126076269 | *** | 0.496000529 |
| 91 | Ethylmalonic acid | 2123 | C₅ H₈ O₄ | NEG | 1.168767915 | *** | 2.015243518 |
| 92 | LPG O-15:1 | 10933 | C₂₁ H₄₃ O₈ P | NEG | 1.171940152 | *** | 0.478195446 |
| 93 | LPE O-17:1 | 10535 | C₂₂ H₄₆ N O₆ P | NEG | 1.172793723 | *** | 0.304572485 |
| 94 | Methyl-beta-galactopyranoside | 1528 | C₇ H₁₄ O₆ | NEG | 1.138844492 | *** | 0.325418056 |
| 95 | LPC 17:1 | 9731 | C₂₅ H₅₀ N O₇ P | NEG | 1.047787225 | *** | 0.264222129 |
| 96 | Orotic acid | 1644 | C₅ H₄ N₂ O₄ | NEG | 1.118426707 | *** | 0.44133712 |
| 97 | 3-[(4-chlorophenyl)thio]-1-(3-pyridylmethyl)pyrrolidine-2,5-dione | 157 | C₁₆ H₁₃ Cl N₂ O₂ S | NEG | 1.161796968 | *** | 3.223417597 |
| 98 | trans-10-Heptadecenoic acid | 1063 | C₁₇ H₃₂ O₂ | NEG | 1.169830164 | *** | 0.30519283 |
| 99 | LPG 14:0 | 9681 | C₂₀ H₄₁ O₉ P | NEG | 1.173725566 | *** | 0.292568535 |
| 100 | 8-iso-15-keto Prostaglandin F2α | 529 | C₂₀ H₃₂ O₅ | NEG | 1.003592202 | *** | 0.480890834 |
| 101 | D-Glucono-1,5-lactone | 1845 | C₆ H₁₀ O₆ | NEG | 1.16928269 | *** | 5.768848149 |
| 102 | trans-Petroselinic Acid | 8241 | C₁₈ H₃₄ O₂ | NEG | 1.169483102 | *** | 0.248898046 |
| 103 | FAHFA 18:1/3:0 | 11124 | C₂₁ H₃₈ O₄ | NEG | 1.164014256 | *** | 0.136997711 |
| 104 | LPC 22:5 | 9752 | C₃₀ H₅₂ N O₇ P | NEG | 1.143300282 | *** | 0.368365688 |
| 105 | 1-(3-phenylpropanoyl)-4-piperidinecarboxylic acid | 7104 | C₁₅ H₁₉ N O₃ | NEG | 1.102774672 | *** | 2.468001733 |
| 106 | LPC 18:3 | 9181 | C₂₆ H₄₈ N O₇ P | NEG | 1.166873006 | *** | 3.02216906 |
| 107 | (+/-)8(9)-DiHET | 8212 | C₂₀ H₃₄ O₄ | NEG | 1.171848617 | *** | 0.410311277 |
| 108 | 8-iso Prostaglandin F2α Ethanolamide | 9281 | C₂₂ H₃₉ N O₅ | NEG | 1.165163587 | *** | 0.32237149 |
| 109 | 2-deoxyglucose-6-phosphate | 1488 | C₆ H₁₃ O₈ P | NEG | 1.141134467 | *** | 2.68527857 |
| 110 | LPC 18:2 | 9232 | C₂₆ H₅₀ N O₇ P | NEG | 1.113568486 | *** | 2.466989926 |
| 111 | Oxytetracycline | 1444 | C₂₂ H₂₄ N₂ O₉ | NEG | 1.145673625 | *** | 3.590064626 |
| 112 | LPG 20:3 | 11409 | C₂₆ H₄₇ O₉ P | NEG | 1.1522336 | *** | 0.109690137 |
| 113 | geranyl pp | 5449 | C₁₀ H₂₀ O₇ P₂ | NEG | 1.122438882 | *** | 2.157267114 |
| 114 | Maltotriose | 5011 | C₁₈ H₃₂ O₁₆ | NEG | 1.171737311 | *** | 4.702055061 |
| 115 | FAHFA 20:4/3:0 | 10476 | C₂₃ H₃₆ O₄ | NEG | 1.158098743 | *** | 0.197922546 |
| 116 | cholesteryl sulfate | 7983 | C₂₇ H₄₆ O₄ S | NEG | 1.115445815 | *** | 2.31187144 |
| 117 | FAHFA 18:2/20:4 | 10477 | C₃₈ H₆₂ O₄ | NEG | 1.146268396 | *** | 2.469877418 |
| 118 | L-Methionine sulfone | 1835 | C₅ H₁₁ N O₄ S | NEG | 1.141078244 | *** | 2.021192786 |
| 119 | 4-(4-cyclohexylphenyl)-4-oxobut-2-enoic acid | 1879 | C₁₆ H₁₈ O₃ | NEG | 1.167406677 | *** | 4.079950017 |
| 120 | Imidazolelactic acid | 2631 | C₆ H₈ N₂ O₃ | NEG | 1.148316887 | *** | 3.209491842 |
| 121 | 3-Methylhippuric acid | 5976 | C₁₀ H₁₁ N O₃ | NEG | 1.162023788 | *** | 2.244053285 |
| 122 | 5-[(Benzoyloxy)methyl]-4,5,6-trihydroxy-2-cyclohexen-1-yl benzoate | 5133 | C₂₁ H₂₀ O₇ | NEG | 1.092957687 | *** | 3.672496173 |
| 123 | S-Sulfo-L-cysteine | 1396 | C₃ H₇ N O₅ S₂ | NEG | 1.16187271 | *** | 4.592467039 |
| 124 | 1-(2,4-diphenyl-2,3-dihydro-1H-1,5-benzodiazepin-1-yl)propan-1-one | 8965 | C₂₄ H₂₂ N₂ O | NEG | 1.172684324 | *** | 0.063459841 |
| 125 | FAHFA 4:0/16:0 | 11199 | C₂₀ H₃₈ O₄ | NEG | 1.165317583 | *** | 0.228391765 |
| 126 | FAHFA 16:0/18:2 | 10839 | C₃₄ H₆₂ O₄ | NEG | 1.163109229 | *** | 3.047362372 |
| 127 | Gluconolactone | 206 | C₆ H₁₀ O₆ | NEG | 1.168779354 | *** | 2.411751565 |
| 128 | 5-(3-chloro-4-methylanilino)-1-methyl-1H-pyrazol-3-ol | 5248 | C₁₁ H₁₂ Cl N₃ O | NEG | 1.105658861 | *** | 0.490895168 |
| 129 | Estriol 17-sulfate | 1916 | C₁₈ H₂₄ O₆ S | NEG | 1.119874331 | *** | 3.250478363 |
| 130 | LPI 22:6 | 1057 | C₃₁ H₄₉ O₁₂ P | NEG | 1.156367124 | *** | 0.353762031 |
| 131 | Kojic acid | 1572 | C₆ H₆ O₄ | NEG | 1.023407802 | *** | 2.246486054 |
| 132 | 3-Nitro-L-Tyrosine | 5393 | C₉ H₁₀ N₂ O₅ | NEG | 1.099517435 | *** | 2.262222287 |
| 133 | 2-Hydroxy-2-methylbutanedioic acid | 5641 | C₅ H₈ O₅ | NEG | 1.156418722 | *** | 2.220133446 |
| 134 | D-Fructose 6-phosphate | 2352 | C₆ H₁₃ O₉ P | NEG | 1.16689464 | *** | 2.021080341 |
| 135 | (+/-)-CP 47,497-C7-Hydroxy metabolite | 9622 | C₂₁ H₃₄ O₃ | NEG | 1.006860618 | *** | 0.466050009 |
| 136 | α,α-Trehalose | 1924 | C₁₂ H₂₂ O₁₁ | NEG | 1.172715379 | *** | 3.427664034 |
| 137 | Cyclic ADP-ribose | 1837 | C₁₅ H₂₁ N₅ O₁₃ P₂ | NEG | 1.139177886 | *** | 6.171730021 |
| 138 | N-(4-chlorophenyl)-N'-(2-phenoxyphenyl)urea | 2011 | C₁₉ H₁₅ Cl N₂ O₂ | NEG | 1.08849741 | *** | 2.967475614 |
| 139 | 3-Methylindole | 5632 | C₉ H₉ N | NEG | 1.017918729 | *** | 2.026830766 |
| 140 | LPS 16:1 | 9281 | C₂₂ H₄₂ N O₉ P | NEG | 1.168842063 | *** | 0.379795108 |
| 141 | LPA 17:1 | 10496 | C₂₀ H₃₉ O₇ P | NEG | 1.173599054 | *** | 0.249058713 |
| 142 | Reduced glutathione | 5529 | C₁₀ H₁₇ N₃ O₆ S | NEG | 1.067547133 | *** | 0.453455425 |
| 143 | 2-Hydroxy-2-methyl-3-buten-1-yl beta-D-glucopyranoside | 2374 | C₁₁ H₂₀ O₇ | NEG | 1.173333284 | *** | 3.472667658 |
| 144 | PE O-17:1_22:6 | 10475 | C₄₄ H₇₆ N O₇ P | NEG | 1.16670289 | *** | 0.348816227 |
| 145 | LPC 16:0 | 9951 | C₂₄ H₅₀ N O₇ P | NEG | 1.173369732 | *** | 0.300162396 |
| 146 | Trehalose | 5061 | C₁₂ H₂₂ O₁₁ | NEG | 1.150439777 | *** | 3.170571497 |
| 147 | 3-Oxo-7alpha,12alpha-hydroxy-5beta-cholanoic acid | 7266 | C₂₄ H₃₈ O₅ | NEG | 1.116414126 | *** | 2.226113009 |
| 148 | 3-[4-methyl-1-(2-methylpropanoyl)-3-oxocyclohexyl]butanoic acid | 627 | C₁₅ H₂₄ O₄ | NEG | 1.1705886 | *** | 3.012217348 |
| 149 | LPE 18:0 | 10593 | C₂₃ H₄₈ N O₇ P | NEG | 1.170318527 | *** | 0.21250097 |
| 150 | Isorhapontigenin | 6638 | C₁₅ H₁₄ O₄ | NEG | 1.171195146 | *** | 5.108890489 |
| 151 | N-Oleoyl Glycine | 10153 | C₂₀ H₃₇ N O₃ | NEG | 1.174388724 | *** | 0.052420599 |
| 152 | ringenin | 6267 | C₁₅ H₁₂ O₅ | NEG | 1.172850312 | *** | 0.382875482 |
| 153 | Arachidonic acid | 10371 | C₂₀ H₃₂ O₂ | NEG | 1.17082967 | *** | 0.148580166 |
| 154 | Tetradecanedioic acid | 763 | C₁₄ H₂₆ O₄ | NEG | 1.076246895 | *** | 2.061642651 |
| 155 | 7-Hydroxy-3,4-dihydrocarbostyril | 5635 | C₉ H₉ N O₂ | NEG | 1.171006931 | *** | 2.030354758 |
| 156 | LPC 16:1 | 9347 | C₂₄ H₄₈ N O₇ P | NEG | 1.144311934 | *** | 0.236332538 |
| 157 | all-cis-4,7,10,13,16-Docosapentaenoic acid | 10572 | C₂₂ H₃₄ O₂ | NEG | 1.169036844 | *** | 0.134526717 |
| 158 | Chenodeoxycholic acid-3-beta-D-glucuronide | 6967 | C₃₀ H₄₈ O₁₀ | NEG | 1.151410364 | *** | 4.091573605 |
| 159 | 10-Undecenoic acid | 5965 | C₁₁ H₂₀ O₂ | NEG | 1.166203133 | *** | 2.304747639 |
| 160 | LPE 20:5 | 9059 | C₂₅ H₄₂ N O₇ P | NEG | 1.13017169 | *** | 0.382866009 |
| 161 | Lysopg 18:1 | 9071 | C₂₄ H₄₇ O₉ P | NEG | 1.171586816 | *** | 0.356712528 |
| 162 | Nonoic acid | 6162 | C₉ H₁₈ O₂ | NEG | 1.168897777 | *** | 2.03562214 |
| 163 | Hydrocortisone | 6256 | C₂₁ H₃₀ O₅ | NEG | 1.170713926 | *** | 0.220599276 |
| 164 | Kynurenic acid O-hexside | 55 | C₁₆ H₁₇ N O₈ | NEG | 1.039102025 | *** | 2.273512888 |
| 165 | Lysope 14:0 | 6579 | C₁₉ H₄₀ N O₇ P | NEG | 1.14535821 | *** | 2.009128642 |
| 166 | Leu-Pro | 5908 | C₁₁ H₂₀ N₂ O₃ | NEG | 1.155287784 | *** | 2.833651333 |
| 167 | 23-Nordeoxycholic acid | 9084 | C₂₃ H₃₈ O₄ | NEG | 1.163834854 | *** | 0.412311395 |
| 168 | Estrone | 712 | C₁₈ H₂₂ O₂ | NEG | 1.148515734 | *** | 2.449731986 |
| 169 | Gamma-Caprolactone | 6075 | C₆ H₁₀ O₂ | NEG | 1.166889743 | *** | 2.204290005 |
| 170 | LPE 22:6 | 9402 | C₂₇ H₄₄ N O₇ P | NEG | 1.17159788 | *** | 0.190490207 |
| 171 | LPC 20:1 | 10936 | C₂₈ H₅₆ N O₇ P | NEG | 1.168899598 | *** | 0.222295754 |
| 172 | LPE 16:0 | 9625 | C₂₁ H₄₄ N O₇ P | NEG | 1.116834839 | *** | 0.480924812 |
| 173 | LysoPE 18:0 | 9949 | C₂₃ H₄₈ N O₇ P | NEG | 1.173420152 | *** | 0.296458877 |
| 174 | Cytidine-5'-monophosphate | 1499 | C₉ H₁₄ N₃ O₈ P | NEG | 1.131083318 | *** | 0.276352803 |
| 175 | 4-Pregnen-17alpha,20alpha-Diol-3-One | 8703 | C₂₁ H₃₂ O₃ | NEG | 1.138984416 | *** | 0.404274036 |
| 176 | Pantetheine | 5649 | C₁₁ H₂₂ N₂ O₄ S | NEG | 1.124286942 | *** | 2.23774237 |
| 177 | LPE 20:3 | 9793 | C₂₅ H₄₆ N O₇ P | NEG | 1.167936072 | *** | 0.094171504 |
| 178 | Cystine | 1248 | C₆ H₁₂ N₂ O₄ S₂ | NEG | 1.063145378 | *** | 2.536695903 |
| 179 | 11-Ketoetiocholanolone | 8085 | C₁₉ H₂₈ O₃ | NEG | 1.153890546 | *** | 0.140093662 |
| 180 | N-Acetylsphingosine | 9975 | C₂₀ H₃₉ N O₃ | NEG | 1.145512979 | *** | 0.14722621 |
| 181 | Estradiol Benzoate | 7924 | C₂₅ H₂₈ O₃ | NEG | 1.172597957 | *** | 3.824387751 |
| 182 | Methyltestosterone | 10461 | C₂₀ H₃₀ O₂ | NEG | 1.173986821 | *** | 0.144442654 |
| 183 | L-Cysteinesulfinic acid | 1445 | C₃ H₇ N O₄ S | NEG | 1.162116712 | *** | 2.026271828 |
| 184 | LPE 18:1 | 10023 | C₂₃ H₄₆ N O₇ P | NEG | 1.173511274 | *** | 0.192899885 |
| 185 | Corey Lactone Diol | 5903 | C₈ H₁₂ O₄ | NEG | 1.148364098 | *** | 0.462677514 |
| 186 | Glu-Glu | 1506 | C₁₀ H₁₆ N₂ O₇ | NEG | 1.143681836 | *** | 0.486866517 |
| 187 | Nicotinic acid | 1988 | C₆ H₅ N O₂ | POS | 1.148254525 | *** | 0.443746439 |
| 188 | L-(+)-Citrulline | 1365 | C₆ H₁₃ N₃ O₃ | POS | 1.164528735 | *** | 2.343097854 |
| 189 | 2-Hydroxycinmic acid | 2954 | C₉ H₈ O₃ | POS | 1.165327407 | *** | 3.092868602 |
| 190 | 2-Amino-1,3,4-octadecanetriol | 8404 | C₁₈ H₃₉ N O₃ | POS | 1.173279092 | *** | 0.135169081 |
| 191 | Urocanic acid | 1948 | C₆ H₆ N₂ O₂ | POS | 1.134196118 | *** | 2.36783656 |
| 192 | N-Benzylformamide | 2945 | C₈ H₉ N O | POS | 1.060346968 | *** | 2.495110824 |
| 193 | Cortisol | 6802 | C₂₁ H₃₀ O₅ | POS | 1.169258699 | *** | 0.09585258 |
| 194 | 4-Methyl-5-thiazoleethanol | 5265 | C₆ H₉ N O S | POS | 1.1692899 | *** | 2.698643654 |
| 195 | 5,7-dihydroxy-3-(4-hydroxyphenyl)-4H-chromen-4-one | 6095 | C₁₅ H₁₀ O₅ | POS | 1.173409763 | *** | 2.946881354 |
| 196 | 3'-Adenosine monophosphate (3'-AMP) | 1817 | C₁₀ H₁₄ N₅ O₇ P | POS | 1.16945925 | *** | 0.20355603 |
| 197 | Creatine | 1444 | C₄ H₉ N₃ O₂ | POS | 1.172611385 | *** | 0.141244952 |
| 198 | Daidzein | 627 | C₁₅ H₁₀ O₄ | POS | 1.174549092 | *** | 5.028245221 |
| 199 | Methionine sulfoxide | 1377 | C₅ H₁₁ N O₃ S | POS | 1.171453363 | *** | 5.892111468 |
| 200 | Eicosapentaenoic acid | 8661 | C₂₀ H₃₀ O₂ | POS | 1.1714248 | *** | 0.379452067 |
| 201 | Taurocholic acid | 7208 | C₂₆ H₄₅ N O₇ S | POS | 1.112949212 | *** | 0.440227031 |
| 202 | LPC 18:1-SN1 | 10178 | C₂₆ H₅₂ N O₇ P | POS | 1.173487533 | *** | 0.250981429 |
| 203 | LPC 22:6-SN1 | 9511 | C₃₀ H₅₀ N O₇ P | POS | 1.167143414 | *** | 0.109985083 |
| 204 | LPC 20:4-SN1 | 9573 | C₂₈ H₅₀ N O₇ P | POS | 1.17389926 | *** | 0.041432632 |
| 205 | Palmitic Acid | 7377 | C₁₆ H₃₂ O₂ | POS | 1.174260558 | *** | 0.41458378 |
| 206 | PC O-20:4 | 9412 | C₂₈ H₅₀ N O₇ P | POS | 1.172592981 | *** | 0.036542202 |
| 207 | PC O-16:0 | 9762 | C₂₄ H₅₀ N O₇ P | POS | 1.173218572 | *** | 0.124247728 |
| 208 | Stearamide | 10985 | C₁₈ H₃₇ N O | POS | 1.163681374 | *** | 4.429787602 |
| 209 | PC O-18:1 | 9968 | C₂₆ H₅₂ N O₇ P | POS | 1.16939007 | *** | 0.151276759 |
| 210 | LPC 22:6-SN2 | 9353 | C₃₀ H₅₀ N O₇ P | POS | 1.173592981 | *** | 0.031434673 |
| 211 | Palmitoylcarnitine | 8827 | C₂₃ H₄₅ N O₄ | POS | 1.174324624 | *** | 0.080745599 |
| 212 | CAR 18:1 | 8983 | C₂₅ H₄₈ N O₄ | POS | 1.174681375 | *** | 0.073555854 |
| 213 | Palmitoyl ethanolamide | 10073 | C₁₈ H₃₇ N O₂ | POS | 1.173447769 | *** | 0.321679513 |
| 214 | PC O-18:0 | 10527 | C₂₆ H₅₄ N O₇ P | POS | 1.173148113 | *** | 0.066064304 |
| 215 | CAR 18:2 | 8597 | C₂₅ H₄₆ N O₄ | POS | 1.16579644 | *** | 0.099364096 |
| 216 | 5-[(10Z)-14-(3,5-dihydroxyphenyl)tetradec-10-en-1-yl]benzene-1,3-diol | 8369 | C₂₆ H₃₆ O₄ | POS | 1.170832025 | *** | 2.01501566 |
| 217 | CAR 20:4 | 8553 | C₂₇ H₄₆ N O₄ | POS | 1.170550335 | *** | 0.049163041 |
| 218 | 5,6-dimethoxy-2-(2-methoxyphenyl)-4H-chromen-4-one | 6013 | C₁₈ H₁₆ O₅ | POS | 1.16813425 | *** | 2.785936197 |
| 219 | 1,4-dihydroxyheptadec-16-en-2-yl acetate | 9223 | C₁₉ H₃₆ O₄ | POS | 1.06884261 | *** | 0.247106456 |
| 220 | PC O-18:2 | 9459 | C₂₆ H₅₀ N O₇ P | POS | 1.174240801 | *** | 0.14675677 |
| 221 | 16(R)-HETE | 8863 | C₂₀ H₃₂ O₃ | POS | 1.148025363 | *** | 0.480021943 |
| 222 | Glutamic acid | 1531 | C₅ H₉ N O₄ | POS | 1.126893513 | *** | 0.292310307 |
| 223 | FMH | 5128 | C₂₀ H₂₇ N₅ O₄ S | POS | 1.171436674 | *** | 0.489103371 |
| 224 | Andamide (AEA) | 9732 | C₂₂ H₃₇ N O₂ | POS | 1.171368196 | *** | 0.060949737 |
| 225 | Tetrahydrocortisone | 7102 | C₂₁ H₃₂ O₅ | POS | 1.17307104 | *** | 0.127103061 |
| 226 | 2-Methoxybenzaldehyde | 6012 | C₈ H₈ O₂ | POS | 1.16813425 | *** | 2.785936197 |
| 227 | 2,4-Dimethylbenzaldehyde | 7058 | C₉ H₁₀ O | POS | 1.170698753 | *** | 2.019799855 |
| 228 | CAR 18:0 | 9414 | C₂₅ H₅₀ N O₄ | POS | 1.174001492 | *** | 0.121125582 |
| 229 | LPC O-14:0 | 9686 | C₂₂ H₄₈ N O₆ P | POS | 1.116794587 | *** | 0.437470004 |
| 230 | N-Acetyl-D-lactosamine | 1437 | C₁₄ H₂₅ N O₁₁ | POS | 1.165982415 | *** | 2.00719939 |
| 231 | 3-amino-2-phenyl-2H-pyrazolo[4,3-c]pyridine-4,6-diol | 6286 | C₁₂ H₁₀ N₄ O₂ | POS | 1.173531451 | *** | 0.247628028 |
| 232 | Mycophenolic acid | 1425 | C₁₇ H₂₀ O₆ | POS | 1.168634073 | *** | 2.952238651 |
| 233 | 4',7-Dihydroxyflavanone | 6168 | C₁₅ H₁₂ O₄ | POS | 1.174050099 | *** | 3.777808243 |
| 234 | LPC 17:0-SN1 | 10408 | C₂₅ H₅₂ N O₇ P | POS | 1.152878554 | *** | 0.112711144 |
| 235 | LPC 14:0-SN1 | 9174 | C₂₂ H₄₆ N O₇ P | POS | 1.172980504 | *** | 0.108745779 |
| 236 | CAR 12:0 | 7605 | C₁₉ H₃₈ N O₄ | POS | 1.174300739 | *** | 0.074202978 |
| 237 | LPC 15:0-SN1 | 9563 | C₂₃ H₄₈ N O₇ P | POS | 1.174011477 | *** | 0.055244179 |
| 238 | CAR 22:6 | 8519 | C₂₉ H₄₆ N O₄ | POS | 1.169433232 | *** | 0.025555829 |
| 239 | N-Methylisoleucine | 2075 | C₇ H₁₅ N O₂ | POS | 1.17239491 | *** | 2.653799549 |
| 240 | U-44069 | 9497 | C₂₁ H₃₄ O₄ | POS | 1.063572057 | *** | 0.169864401 |
| 241 | 7-Ketocholesterol | 10368 | C₂₇ H₄₄ O₂ | POS | 1.15894952 | *** | 0.478408733 |
| 242 | PC O-16:1 | 9203 | C₂₄ H₄₈ N O₇ P | POS | 1.162513709 | *** | 0.089212286 |
| 243 | LPC 20:1-SN1 | 11016 | C₂₈ H₅₆ N O₇ P | POS | 1.173629628 | *** | 0.111514564 |
| 244 | Stearoyl Ethanolamide | 10868 | C₂₀ H₄₁ N O₂ | POS | 1.172659384 | *** | 0.26734432 |
| 245 | L-Homocitrulline | 1542 | C₇ H₁₅ N₃ O₃ | POS | 1.158530577 | *** | 0.383644533 |
| 246 | 9-Oxo-10(E),12(E)-octadecadienoic acid | 8868 | C₁₈ H₃₀ O₃ | POS | 1.170243278 | *** | 73.27929984 |
| 247 | 2-Arachidonoyl glycerol | 9819 | C₂₃ H₃₈ O₄ | POS | 1.171684611 | *** | 0.124166337 |
| 248 | L-Alanyl-L-proline | 2167 | C₈ H₁₄ N₂ O₃ | POS | 1.173280124 | *** | 3.748709225 |
| 249 | VLK | 5391 | C₁₇ H₃₄ N₄ O₄ | POS | 1.17147108 | *** | 0.496705313 |
| 250 | Lysopc 18:2 | 10508 | C₂₆ H₅₀ N O₇ P | POS | 1.173419921 | *** | 0.022186362 |
| 251 | LPC 20:3-SN1 | 9934 | C₂₈ H₅₂ N O₇ P | POS | 1.171812949 | *** | 0.067671839 |
| 252 | 4-oxododecanedioic acid | 5915 | C₁₂ H₂₀ O₅ | POS | 1.165701563 | *** | 2.306984199 |
| 253 | LysoPC 20:2 | 10437 | C₂₈ H₅₄ N O₇ P | POS | 1.172464986 | *** | 0.077619194 |
| 254 | LPC 22:4-SN1 | 10252 | C₃₀ H₅₄ N O₇ P | POS | 1.128505807 | *** | 0.25009874 |
| 255 | Kahweol | 6168 | C₂₀ H₂₆ O₃ | POS | 1.168586873 | *** | 0.419574885 |
| 256 | CAR 20:1 | 9541 | C₂₇ H₅₂ N O₄ | POS | 1.172199138 | *** | 0.135202174 |
| 257 | Canbidiolic acid | 7603 | C₂₂ H₃₀ O₄ | POS | 1.150544794 | *** | 0.461109329 |
| 258 | LLK | 5569 | C₁₈ H₃₆ N₄ O₄ | POS | 1.171957967 | *** | 0.470196982 |
| 259 | PC O-22:4 | 10054 | C₃₀ H₅₄ N O₇ P | POS | 1.158587918 | *** | 0.225474155 |
| 260 | CAR 20:5 | 824 | C₂₇ H₄₄ N O₄ | POS | 1.173896755 | *** | 0.113766829 |
| 261 | KLK | 8216 | C₁₈ H₃₇ N₅ O₄ | POS | 1.168167689 | *** | 0.437499131 |
| 262 | 15(R)-Prostaglandin D2 | 7058 | C₂₀ H₃₂ O₅ | POS | 1.174233714 | *** | 0.324444325 |
| 263 | Arachidonoyl amide | 7813 | C₂₀ H₃₃ N O | POS | 1.155177664 | *** | 3.120592174 |
| 264 | Glu-Val-Phe | 5649 | C₁₉ H₂₇ N₃ O₆ | POS | 1.102712682 | *** | 0.490466637 |
| 265 | Palmitoleic Acid | 10181 | C₁₆ H₃₀ O₂ | POS | 1.16279385 | *** | 0.428317075 |
| 266 | Octadecamine | 9317 | C₁₈ H₃₉ N | POS | 1.17428622 | *** | 0.091597987 |
| 267 | 11-Deoxy prostaglandin F1α | 8801 | C₂₀ H₃₆ O₄ | POS | 1.096775322 | *** | 0.394241077 |
| 268 | Deoxyinosine | 5091 | C₁₀ H₁₂ N₄ O₄ | POS | 1.15953193 | *** | 2.267169281 |
| 269 | 2,4-dihydroxyheptadec-16-en-1-yl acetate | 7655 | C₁₉ H₃₆ O₄ | POS | 1.165999148 | *** | 2.812818996 |
| 270 | CAR 16:1 | 8637 | C₂₃ H₄₄ N O₄ | POS | 1.168094881 | *** | 0.066172479 |
| 271 | PC O-14:0 | 8979 | C₂₂ H₄₆ N O₇ P | POS | 1.128451277 | *** | 0.079255625 |
| 272 | 4-Hexyloxyaniline | 7204 | C₁₂ H₁₉ N O | POS | 1.173379022 | *** | 0.251483124 |
| 273 | Docosapentaenoic acid | 10618 | C₂₂ H₃₄ O₂ | POS | 1.160115369 | *** | 0.141198886 |
| 274 | 11(Z),14(Z),17(Z)-Eicosatrienoic acid | 10801 | C₂₀ H₃₄ O₂ | POS | 1.170083383 | *** | 0.113609248 |
| 275 | Andrographolide | 6843 | C₂₀ H₃₀ O₅ | POS | 1.173349542 | *** | 0.473832917 |
| 276 | CAR 20:2 | 9164 | C₂₇ H₅₀ N O₄ | POS | 1.165932367 | *** | 0.112788612 |
| 277 | PC O-17:1 | 9583 | C₂₅ H₅₀ N O₇ P | POS | 1.171172491 | *** | 0.154259375 |
| 278 | Tauroursodeoxycholic acid | 7996 | C₂₆ H₄₅ N O₆ S | POS | 1.155978708 | *** | 0.499329177 |
| 279 | N6-Isopentenyladenosine | 6296 | C₁₅ H₂₁ N₅ O₄ | POS | 1.0282651 | *** | 0.429870479 |
| 280 | FRH | 10006 | C₂₁ H₃₀ N₈ O₄ | POS | 1.171047523 | *** | 0.208464023 |
| 281 | 2-hydroxy-6-[(8Z,11Z)-pentadeca-8,11,14-trien-1-yl]benzoic acid | 1052 | C₂₂ H₃₀ O₃ | POS | 1.123652805 | *** | 2.343711936 |
| 282 | 16,16-Dimethyl prostaglandin A1 | 9201 | C₂₂ H₃₆ O₄ | POS | 1.005033741 | *** | 0.411562471 |
| 283 | 6 β-Hydroxycortisol | 5855 | C₂₁ H₃₀ O₆ | POS | 1.162216672 | *** | 0.175520857 |
| 284 | ALK | 5105 | C₁₅ H₃₀ N₄ O₄ | POS | 1.064518597 | *** | 0.379162583 |
| 285 | LPE 17:0 | 10239 | C₂₂ H₄₆ N O₇ P | POS | 1.039810624 | *** | 0.402717934 |
| 286 | CAR 17:1 | 866 | C₂₄ H₄₆ N O₄ | POS | 1.146148842 | *** | 0.077053264 |
| 287 | 2-(2,6-dimethoxyphenyl)-5,6-dimethoxy-4H-chromen-4-one | 6149 | C₁₉ H₁₈ O₆ | POS | 1.114179562 | *** | 2.639407955 |
| 288 | [5-(2-thienyl)-3-isoxazolyl]methanol | 1535 | C₈ H₇ N O₂ S | POS | 1.16721604 | *** | 2.134826405 |
| 289 | N-Acetyl-5-aminosalicylic acid | 1536 | C₉ H₉ N O₄ | POS | 1.169924595 | *** | 2.124233775 |
| 290 | Caffeine | 1374 | C₈ H₁₀ N₄ O₂ | POS | 1.028426881 | *** | 0.064884136 |
| 291 | LPC 16:0-SN1 | 10523 | C₂₄ H₅₀ N O₇ P | POS | 1.174391983 | *** | 0.097851886 |
| 292 | 6-methyl-4-(morpholinomethyl)-2H-chromen-2-one | 2056 | C₁₅ H₁₇ N O₃ | POS | 1.072369533 | *** | 2.259310105 |
| 293 | PC O-20:5 | 899 | C₂₈ H₄₈ N O₇ P | POS | 1.174001104 | *** | 0.058732203 |
| 294 | PC O-20:1 | 10749 | C₂₈ H₅₆ N O₇ P | POS | 1.170544043 | *** | 0.107502004 |
| 295 | PC O-20:2 | 10238 | C₂₈ H₅₄ N O₇ P | POS | 1.164853082 | *** | 0.084571315 |
| 296 | LPC 18:0-SN1 | 11312 | C₂₆ H₅₄ N O₇ P | POS | 1.173884693 | *** | 0.117094128 |
| 297 | LPC 20:5-SN1 | 915 | C₂₈ H₄₈ N O₇ P | POS | 1.164484153 | *** | 0.229854571 |
| 298 | N-acetyl-L-ornithine | 2946 | C₇ H₁₄ N₂ O₃ | POS | 1.166211699 | *** | 0.073051333 |
| 299 | 4-Guanidinobutanoic acid | 1887 | C₅ H₁₁ N₃ O₂ | POS | 1.157409328 | *** | 3.163140383 |
| 300 | 1,4-dihydroxy-1,4-dimethyl-7-(propan-2-ylidene)-decahydroazulen-6-one | 8675 | C₁₅ H₂₄ O₃ | POS | 1.162128122 | *** | 0.352291496 |
| 301 | 4-oxo-4-[(1-phenylethyl)amino]but-2-enoic acid | 5142 | C₁₂ H₁₃ N O₃ | POS | 1.159338313 | *** | 2.103115175 |
| 302 | SNH | 2362 | C₁₃ H₂₀ N₆ O₆ | POS | 1.116637756 | *** | 3.44422349 |
| 303 | 1-methyl-3-phenyl-1H-pyrazol-5-amine | 8353 | C₁₀ H₁₁ N₃ | POS | 1.148688259 | *** | 2.221488647 |
| 304 | LPC 17:1-SN1 | 9803 | C₂₅ H₅₀ N O₇ P | POS | 1.159918151 | *** | 0.098516015 |
| 305 | PC 19:0_19:1 | 10872 | C₄₆ H₉₀ N O₈ P | POS | 1.16755024 | *** | 0.455761548 |
| 306 | PPK | 239 | C₁₆ H₂₈ N₄ O₄ | POS | 1.170807191 | *** | 5.566105832 |
| 307 | dAMP | 3873 | C₁₀ H₁₄ N₅ O₆ P | POS | 1.169843228 | *** | 3.030481896 |
| 308 | 4-methyl-6-[(4-methylphenyl)thio]-2-(2-pyridyl)pyrimidine | 2419 | C₁₇ H₁₅ N₃ S | POS | 1.150977363 | *** | 0.375747335 |
| 309 | Stercobilin | 7046 | C₃₃ H₄₆ N₄ O₆ | POS | 1.036284054 | *** | 0.474453587 |
| 310 | 2-{[methyl(2,3,4,5,6-pentahydroxyhexyl)amino]methylidene}malononitrile | 4564 | C₁₁ H₁₇ N₃ O₅ | POS | 1.168568941 | *** | 2.120356807 |
| 311 | 16-Heptadecyne-1,2,4-triol | 8564 | C₁₇ H₃₂ O₃ | POS | 1.127288315 | *** | 0.456572616 |
| 312 | Linoleoyl ethanolamide | 9755 | C₂₀ H₃₇ N O₂ | POS | 1.173704374 | *** | 0.393848243 |
| 313 | LPC O-16:1 | 10349 | C₂₄ H₅₀ N O₆ P | POS | 1.170994191 | *** | 0.05170211 |
| 314 | Oleoyl ethanolamide | 10289 | C₂₀ H₃₉ N O₂ | POS | 1.169885073 | *** | 0.353722821 |
| 315 | Leucylproline | 5478 | C₁₁ H₂₀ N₂ O₃ | POS | 1.172573754 | *** | 2.39174132 |
| 316 | LPE 20:4 | 9499 | C₂₅ H₄₄ N O₇ P | POS | 1.173770746 | *** | 0.097175402 |
| 317 | LPC O-18:1 | 10695 | C₂₆ H₅₄ N O₆ P | POS | 1.174417328 | *** | 0.064530912 |
| 318 | Sphingosine (d18:1) | 807 | C₁₈ H₃₇ N O₂ | POS | 1.1731225 | *** | 2.373104055 |
| 319 | Oxaceprol | 1565 | C₇ H₁₁ N O₄ | POS | 1.170822078 | *** | 2.14626836 |
| 320 | Docosahexaenoyl Ethanolamide | 9679 | C₂₄ H₃₇ N O₂ | POS | 1.174333976 | *** | 0.103528847 |
| 321 | N-(5-Aminopentyl)acetamide | 1558 | C₇ H₁₆ N₂ O | POS | 1.169983581 | *** | 0.180436356 |
| 322 | L-Leucyl-L-Alanine | 5151 | C₉ H₁₈ N₂ O₃ | POS | 1.133161823 | *** | 0.383384173 |
| 323 | LPC O-15:0 | 10096 | C₂₃ H₅₀ N O₆ P | POS | 1.173767468 | *** | 0.134790511 |
| 324 | LPC O-15:1 | 9924 | C₂₃ H₄₈ N O₆ P | POS | 1.133589801 | *** | 0.122043323 |
| 325 | cis-7-Hexadecenoic Acid | 8862 | C₁₆ H₃₀ O₂ | POS | 1.15055118 | *** | 2.482195411 |
| 326 | LPE 16:1 | 9287 | C₂₁ H₄₂ N O₇ P | POS | 1.173296265 | *** | 0.09710354 |
| 327 | LPC O-18:3 | 10013 | C₂₆ H₅₀ N O₆ P | POS | 1.170518733 | *** | 0.058723621 |
| 328 | LPC O-18:0 | 11368 | C₂₆ H₅₆ N O₆ P | POS | 1.17176291 | *** | 0.231113102 |
| 329 | 12(S)-HETE | 8104 | C₂₀ H₃₂ O₃ | POS | 1.159844154 | *** | 0.378480031 |
| 330 | N6-Methyladenine | 5278 | C₆ H₇ N₅ | POS | 1.140070569 | *** | 2.002575482 |
| 331 | Lithocholic acid | 10355 | C₂₄ H₄₀ O₃ | POS | 1.169335075 | *** | 0.063401262 |
| 332 | JNJ-1661010 | 1459 | C₁₉ H₁₉ N₅ O S | POS | 1.160788012 | *** | 2.333261301 |
| 333 | LPE 22:5 | 9696 | C₂₇ H₄₆ N O₇ P | POS | 1.133232138 | *** | 0.251228525 |
| 334 | Ne-(1-Carboxymethyl)-L-lysine | 2369 | C₈ H₁₆ N₂ O₄ | POS | 1.17088193 | *** | 0.031858405 |
| 335 | Phenylpropiolic acid | 2976 | C₉ H₆ O₂ | POS | 1.171547083 | *** | 2.739520672 |
| 336 | O-Phospho-L-serine | 1337 | C₃ H₈ N O₆ P | POS | 1.157985207 | *** | 0.258473324 |
| 337 | Linolelaidic Acid (C18:2N6T) | 7785 | C₁₈ H₃₂ O₂ | POS | 1.162187787 | *** | 0.252031184 |
| 338 | N-Acetyl-aspartic acid | 1571 | C₆ H₉ N O₅ | POS | 1.023823065 | *** | 0.25948534 |
| 339 | Sodium cholate | 6985 | C₂₄ H₃₉ O₅ | POS | 1.092331813 | *** | 2.232978366 |
| 340 | LPC 20:2 | 10484 | C₂₈ H₅₄ N O₇ P | POS | 1.170943232 | *** | 0.266686336 |
| 341 | Desthiobiotin | 4584 | C₁₀ H₁₈ N₂ O₃ | POS | 1.160992867 | *** | 2.166642106 |
| 342 | 17α-Hydroxyprogesterone | 5956 | C₂₁ H₃₀ O₃ | POS | 1.1597612 | *** | 0.21843193 |
| 343 | 2-Deoxyuridine | 4015 | C₉ H₁₂ N₂ O₅ | POS | 1.142011819 | *** | 2.643324711 |
| 344 | LPC O-17:0 | 10957 | C₂₅ H₅₄ N O₆ P | POS | 1.135893358 | *** | 0.303406996 |
| 345 | N-Formylkynurenine | 485 | C₁₁ H₁₂ N₂ O₄ | POS | 1.169225082 | *** | 2.553679114 |
| 346 | P-Coumaroyl Agmatine | 5482 | C₁₄ H₂₀ N₄ O₂ | POS | 1.108383213 | *** | 2.297920357 |
| 347 | L-beta-Imidazolelactic acid | 2625 | C₆ H₈ N₂ O₃ | POS | 1.16548552 | *** | 0.491122945 |

^*^*P*<0.05，^**^*P*<0.01，^***^*P*<0.001 vs. the CUMS group

**Table S5 Differential metabolites in hippocampal samples**

| **NO** | **t_R_(min)** | **Metabolite** | **Formula** | **Ionizationmode** | **CUMS vs. CON** | | |  | **SCFs vs. CUMS** | |
| --- | --- | --- | --- | --- | --- | --- | --- | --- | --- | --- |
|  |  |  |  |  | **VIP** | **Fold** | **Ttrend** | **VIP** | **Fold** | **Trend** |
|  |  |  |  |  | **score** | **change** |  | **score** | **change** |  |
| 1 | 9686 | LPC O-14:0 | C₂₂ H₄₈ N O₆ P | NEG | 1.16521035 | 0.003487245 | ↓ △△△ | 1.116794587 | 0.437470004 | ↓ *** |
| 2 | 9353 | LPC 22:6-SN2 | C₃₀ H₅₀ N O₇ P | NEG | 1.165128366 | 0.013369295 | ↓ △△△ | 1.173592981 | 0.031434673 | ↓ *** |
| 3 | 1237 | D-Glucose 6-phosphate | C₆ H₁₃ O₉ P | NEG | 1.094736021 | 0.023755326 | ↓ △△△ | 1.100919518 | 0.043639039 | ↓ *** |
| 4 | 9477 | LPC 22:6 | C₃₀ H₅₀ N O₇ P | POS | 1.164617007 | 0.025108338 | ↓ △△△ | 1.171536756 | 0.278677169 | ↓ *** |
| 5 | 9347 | LPC 16:1 | C₂₄ H₄₈ N O₇ P | NEG | 1.151662954 | 0.029093094 | ↓ △△△ | 1.144311934 | 0.236332538 | ↓ *** |
| 6 | 1444 | Creatine | C₄ H₉ N₃ O₂ | POS | 1.164341381 | 0.034284015 | ↓ △△△ | 1.172611385 | 0.141244952 | ↓ *** |
| 7 | 915 | LPC 20:5-SN1 | C₂₈ H₄₈ N O₇ P | NEG | 1.163420045 | 0.03591499 | ↓ △△△ | 1.164484153 | 0.229854571 | ↓ *** |
| 8 | 10593 | LPE 18:0 | C₂₃ H₄₈ N O₇ P | NEG | 1.163353952 | 0.03618026 | ↓ △△△ | 1.170318527 | 0.21250097 | ↓ *** |
| 9 | 8519 | CAR 22:6 | C₂₉ H₄₆ N O₄ | NEG | 1.160531363 | 0.036823754 | ↓ △△△ | 1.169433232 | 0.025555829 | ↓ *** |
| 10 | 9861 | LPE O-18:3 | C₂₃ H₄₄ N O₆ P | NEG | 1.165605415 | 0.036869392 | ↓ △△△ | 1.174195841 | 0.0653045 | ↓ *** |
| 11 | 1366 | Gluconic acid | C₆ H₁₂ O₇ | NEG | 1.15348252 | 0.037643101 | ↓ △△△ | 1.170013472 | 3.728770723 | ↑ *** |
| 12 | 9511 | LPC 22:6-SN1 | C₃₀ H₅₀ N O₇ P | POS | 1.159563144 | 0.039473303 | ↓ △△△ | 1.167143414 | 0.109985083 | ↓ *** |
| 13 | 7204 | 4-Hexyloxyaniline | C₁₂ H₁₉ N O | POS | 1.165420555 | 0.047717893 | ↓ △△△ | 1.173379022 | 0.251483124 | ↓ *** |
| 14 | 8216 | KLK | C₁₈ H₃₇ N₅ O₄ | NEG | 1.162604364 | 0.047874223 | ↓ △△△ | 1.168167689 | 0.437499131 | ↓ *** |
| 15 | 7102 | Tetrahydrocortisone | C₂₁ H₃₂ O₅ | POS | 1.164878869 | 0.050570564 | ↓ △△△ | 1.17307104 | 0.127103061 | ↓ *** |
| 16 | 6075 | 2-Hydroxycaproic acid | C₆ H₁₂ O₃ | NEG | 1.164545798 | 0.051598474 | ↓ △△△ | 1.168234374 | 0.476764189 | ↓ *** |
| 17 | 10006 | FRH | C₂₁ H₃₀ N₈ O₄ | POS | 1.163635314 | 0.052079371 | ↓ △△△ | 1.171047523 | 0.208464023 | ↓ *** |
| 18 | 867 | 8(S),15(S)-DiHETE | C₂₀ H₃₂ O₄ | POS | 1.161544785 | 0.052816429 | ↓ △△△ | 1.168683744 | 0.20866493 | ↓ *** |
| 19 | 1374 | Caffeine | C₈ H₁₀ N₄ O₂ | NEG | 1.023098105 | 0.054256248 | ↓ △△△ | 1.028426881 | 0.064884136 | ↓ *** |
| 20 | 8601 | (+/-)5(6)-DiHET | C₂₀ H₃₄ O₄ | NEG | 1.163382121 | 0.054936098 | ↓ △△△ | 1.171429108 | 0.12074937 | ↓ *** |
| 21 | 9412 | PC O-20:4 | C₂₈ H₅₀ N O₇ P | NEG | 1.163924398 | 0.055913645 | ↓ △△△ | 1.172592981 | 0.036542202 | ↓ *** |
| 22 | 2011 | N-(4-chlorophenyl)-N'-(2-phenoxyphenyl)urea | C₁₉ H₁₅ Cl N₂ O₂ | NEG | 1.154021111 | 0.056086338 | ↓ △△△ | 1.08849741 | 2.967475614 | ↑ *** |
| 23 | 6802 | Cortisol | C₂₁ H₃₀ O₅ | NEG | 1.161534527 | 0.056666282 | ↓ △△△ | 1.169258699 | 0.09585258 | ↓ *** |
| 24 | 10348 | LPE O-18:2 | C₂₃ H₄₆ N O₆ P | POS | 1.157501557 | 0.056800337 | ↓ △△△ | 1.162501618 | 0.198976019 | ↓ *** |
| 25 | 295 | Thymidine 5'-monophosphate | C₁₀ H₁₅ N₂ O₈ P | POS | 1.162403406 | 0.0569696 | ↓ △△△ | 1.168856991 | 0.211828124 | ↓ *** |
| 26 | 10238 | PC O-20:2 | C₂₈ H₅₄ N O₇ P | POS | 1.156964695 | 0.057052379 | ↓ △△△ | 1.164853082 | 0.084571315 | ↓ *** |
| 27 | 899 | PC O-20:5 | C₂₈ H₄₈ N O₇ P | NEG | 1.165569268 | 0.057229328 | ↓ △△△ | 1.174001104 | 0.058732203 | ↓ *** |
| 28 | 1109 | LPE O-18:0 | C₂₃ H₅₀ N O₆ P | NEG | 1.162283506 | 0.057893796 | ↓ △△△ | 1.160600639 | 0.494907415 | ↓ *** |
| 29 | 7208 | Taurocholic acid | C₂₆ H₄₅ N O₇ S | NEG | 1.15186796 | 0.061587602 | ↓ △△△ | 1.112949212 | 0.440227031 | ↓ *** |
| 30 | 9793 | LPE 20:3 | C₂₅ H₄₆ N O₇ P | POS | 1.159512853 | 0.064233817 | ↓ △△△ | 1.167936072 | 0.094171504 | ↓ *** |
| 31 | 11312 | LPC 18:0-SN1 | C₂₆ H₅₄ N O₇ P | NEG | 1.165503324 | 0.068494275 | ↓ △△△ | 1.173884693 | 0.117094128 | ↓ *** |
| 32 | 9728 | Acardic acid | C₂₂ H₃₆ O₃ | POS | 1.164743809 | 0.0696019 | ↓ △△△ | 1.17121039 | 0.18123885 | ↓ *** |
| 33 | 10535 | LPE O-17:1 | C₂₂ H₄₆ N O₆ P | NEG | 1.16564898 | 0.069640242 | ↓ △△△ | 1.172793723 | 0.304572485 | ↓ *** |
| 34 | 6703 | Aldosterone | C₂₁ H₂₈ O₅ | NEG | 1.159408178 | 0.070066531 | ↓ △△△ | 1.161373177 | 0.301137245 | ↓ *** |
| 35 | 11368 | LPC O-18:0 | C₂₆ H₅₆ N O₆ P | NEG | 1.164160999 | 0.071956221 | ↓ △△△ | 1.17176291 | 0.231113102 | ↓ *** |
| 36 | 10695 | LPC O-18:1 | C₂₆ H₅₄ N O₆ P | POS | 1.165792379 | 0.07446631 | ↓ △△△ | 1.174417328 | 0.064530912 | ↓ *** |
| 37 | 10752 | Lysopc 17:0 | C₂₅ H₅₂ N O₇ P | NEG | 1.164211674 | 0.075051487 | ↓ △△△ | 1.171414445 | 0.261393754 | ↓ *** |
| 38 | 10167 | LPE O-16:1 | C₂₁ H₄₄ N O₆ P | NEG | 1.166025448 | 0.07625374 | ↓ △△△ | 1.174443426 | 0.203001299 | ↓ *** |
| 39 | 7368 | ST 24:1;O4;T | C₂₆ H₄₅ N O₇ S | POS | 1.16167541 | 0.077047556 | ↓ △△△ | 1.16147544 | 0.452485756 | ↓ *** |
| 40 | 10752 | LPC 18:0 | C₂₆ H₅₄ N O₇ P | NEG | 1.165016729 | 0.078024717 | ↓ △△△ | 1.172721921 | 0.262352258 | ↓ *** |
| 41 | 10466 | LPE O-18:1 | C₂₃ H₄₈ N O₆ P | NEG | 1.049605017 | 0.080643637 | ↓ △△△ | 1.006958659 | 0.258014882 | ↓ *** |
| 42 | 7813 | Arachidonoyl amide | C₂₀ H₃₃ N O | POS | 1.146388402 | 0.081819867 | ↓ △△△ | 1.155177664 | 3.120592174 | ↑ *** |
| 43 | 5855 | 6 β-Hydroxycortisol | C₂₁ H₃₀ O₆ | POS | 1.155957467 | 0.083922832 | ↓ △△△ | 1.162216672 | 0.175520857 | ↓ *** |
| 44 | 10013 | LPC O-18:3 | C₂₆ H₅₀ N O₆ P | POS | 1.161554023 | 0.083965421 | ↓ △△△ | 1.170518733 | 0.058723621 | ↓ *** |
| 45 | 10262 | LPG 16:1 | C₂₂ H₄₃ O₉ P | POS | 1.023446484 | 0.084008007 | ↓ △△△ | 1.007145394 | 0.167409243 | ↓ *** |
| 46 | 8703 | 4-Pregnen-17alpha,20alpha-Diol-3-One | C₂₁ H₃₂ O₃ | NEG | 1.152713611 | 0.085309745 | ↓ △△△ | 1.138984416 | 0.404274036 | ↓ *** |
| 47 | 99 | LPC 20:3 | C₂₈ H₅₂ N O₇ P | POS | 1.164537818 | 0.085622581 | ↓ △△△ | 1.173065146 | 0.052995707 | ↓ *** |
| 48 | 7603 | Canbidiolic acid | C₂₂ H₃₀ O₄ | POS | 1.157459598 | 0.085635177 | ↓ △△△ | 1.150544794 | 0.461109329 | ↓ *** |
| 49 | 9203 | PC O-16:1 | C₂₄ H₄₈ N O₇ P | NEG | 1.154151219 | 0.086094161 | ↓ △△△ | 1.162513709 | 0.089212286 | ↓ *** |
| 50 | 863 | (±)11-HETE | C₂₀ H₃₂ O₃ | NEG | 1.163964324 | 0.086769491 | ↓ △△△ | 1.169786261 | 0.371006567 | ↓ *** |
| 51 | 10936 | LPC 20:1 | C₂₈ H₅₆ N O₇ P | POS | 1.162182368 | 0.087489784 | ↓ △△△ | 1.168899598 | 0.222295754 | ↓ *** |
| 52 | 8085 | 11-Ketoetiocholanolone | C₁₉ H₂₈ O₃ | NEG | 1.148056582 | 0.087949224 | ↓ △△△ | 1.153890546 | 0.140093662 | ↓ *** |
| 53 | 10349 | LPC O-16:1 | C₂₄ H₅₀ N O₆ P | POS | 1.161996326 | 0.088134233 | ↓ △△△ | 1.170994191 | 0.05170211 | ↓ *** |
| 54 | 5569 | LLK | C₁₈ H₃₆ N₄ O₄ | NEG | 1.164955147 | 0.090814978 | ↓ △△△ | 1.171957967 | 0.470196982 | ↓ *** |
| 55 | 10023 | LPE 18:1 | C₂₃ H₄₆ N O₇ P | POS | 1.165224277 | 0.092561743 | ↓ △△△ | 1.173511274 | 0.192899885 | ↓ *** |
| 56 | 9174 | LPC 14:0-SN1 | C₂₂ H₄₆ N O₇ P | POS | 1.164463734 | 0.094550896 | ↓ △△△ | 1.172980504 | 0.108745779 | ↓ *** |
| 57 | 9402 | LPE 22:6 | C₂₇ H₄₄ N O₇ P | POS | 1.162659577 | 0.101031936 | ↓ △△△ | 1.17159788 | 0.190490207 | ↓ *** |
| 58 | 10523 | LPC 16:0-SN1 | C₂₄ H₅₀ N O₇ P | POS | 1.165562725 | 0.109234491 | ↓ △△△ | 1.174391983 | 0.097851886 | ↓ *** |
| 59 | 8404 | 2-Amino-1,3,4-octadecanetriol | C₁₈ H₃₉ N O₃ | NEG | 1.164753209 | 0.109907071 | ↓ △△△ | 1.173279092 | 0.135169081 | ↓ *** |
| 60 | 9602 | LPS 15:0 | C₂₁ H₄₂ N O₉ P | POS | 1.161629558 | 0.112874703 | ↓ △△△ | 1.164407019 | 0.408452991 | ↓ *** |
| 61 | 8965 | 1-(2,4-diphenyl-2,3-dihydro-1H-1,5-benzodiazepin-1-yl)propan-1-one | C₂₄ H₂₂ N₂ O | NEG | 1.163875719 | 0.115239745 | ↓ △△△ | 1.172684324 | 0.063459841 | ↓ *** |
| 62 | 11409 | LPG 20:3 | C₂₆ H₄₇ O₉ P | POS | 1.144009123 | 0.115469054 | ↓ △△△ | 1.1522336 | 0.109690137 | ↓ *** |
| 63 | 9949 | LysoPE 18:0 | C₂₃ H₄₈ N O₇ P | POS | 1.165662224 | 0.115577105 | ↓ △△△ | 1.173420152 | 0.296458877 | ↓ *** |
| 64 | 9934 | LPC 20:3-SN1 | C₂₈ H₅₂ N O₇ P | POS | 1.162580911 | 0.116727597 | ↓ △△△ | 1.171812949 | 0.067671839 | ↓ *** |
| 65 | 1916 | Estriol 17-sulfate | C₁₈ H₂₄ O₆ S | POS | 1.125019876 | 0.117557618 | ↓ △△△ | 1.119874331 | 3.250478363 | ↑ *** |
| 66 | 11016 | LPC 20:1-SN1 | C₂₈ H₅₆ N O₇ P | NEG | 1.164964374 | 0.119022934 | ↓ △△△ | 1.173629628 | 0.111514564 | ↓ *** |
| 67 | 9951 | LPC 16:0 | C₂₄ H₅₀ N O₇ P | NEG | 1.165709354 | 0.12071356 | ↓ △△△ | 1.173369732 | 0.300162396 | ↓ *** |
| 68 | 1412 | Xylitol | C₅ H₁₂ O₅ | POS | 1.121631694 | 0.12075218 | ↓ △△△ | 1.077459403 | 0.347620759 | ↓ *** |
| 69 | 10096 | LPC O-15:0 | C₂₃ H₅₀ N O₆ P | NEG | 1.15891468 | 0.126553502 | ↓ △△△ | 1.173767468 | 0.134790511 | ↓ *** |
| 70 | 9059 | LPE 20:5 | C₂₅ H₄₂ N O₇ P | NEG | 1.152516609 | 0.128080261 | ↓ △△△ | 1.13017169 | 0.382866009 | ↓ *** |
| 71 | 5391 | VLK | C₁₇ H₃₄ N₄ O₄ | NEG | 1.165397301 | 0.129892307 | ↓ △△△ | 1.17147108 | 0.496705313 | ↓ *** |
| 72 | 9573 | LPC 20:4-SN1 | C₂₈ H₅₀ N O₇ P | NEG | 1.164676563 | 0.130635592 | ↓ △△△ | 1.17389926 | 0.041432632 | ↓ *** |
| 73 | 10512 | LPS 18:1 | C₂₄ H₄₆ N O₉ P | POS | 1.033821439 | 0.130962304 | ↓ △△△ | 1.052830021 | 0.081420884 | ↓ *** |
| 74 | 9287 | LPE 16:1 | C₂₁ H₄₂ N O₇ P | NEG | 1.164580321 | 0.133266185 | ↓ △△△ | 1.173296265 | 0.09710354 | ↓ *** |
| 75 | 10461 | Methyltestosterone | C₂₀ H₃₀ O₂ | POS | 1.165314678 | 0.13710121 | ↓ △△△ | 1.173986821 | 0.144442654 | ↓ *** |
| 76 | 8799 | (±)11(12)-EET | C₂₀ H₃₂ O₃ | POS | 1.161000648 | 0.138373862 | ↓ △△△ | 1.130667851 | 0.422951566 | ↓ *** |
| 77 | 8127 | 15(S)-HpETE | C₂₀ H₃₂ O₄ | NEG | 1.156005901 | 0.14101203 | ↓ △△△ | 1.144722359 | 0.47088845 | ↓ *** |
| 78 | 9317 | Octadecamine | C₁₈ H₃₉ N | NEG | 1.164193879 | 0.145141567 | ↓ △△△ | 1.17428622 | 0.091597987 | ↓ *** |
| 79 | 10437 | LysoPC 20:2 | C₂₈ H₅₄ N O₇ P | NEG | 1.163226416 | 0.147016788 | ↓ △△△ | 1.172464986 | 0.077619194 | ↓ *** |
| 80 | 5956 | 17α-Hydroxyprogesterone | C₂₁ H₃₀ O₃ | POS | 1.153241894 | 0.147738974 | ↓ △△△ | 1.1597612 | 0.21843193 | ↓ *** |
| 81 | 1813 | Adenosine 5'-monophosphate | C₁₀ H₁₄ N₅ O₇ P | POS | 1.151739066 | 0.147756403 | ↓ △△△ | 1.150997552 | 0.313415843 | ↓ *** |
| 82 | 9563 | LPC 15:0-SN1 | C₂₃ H₄₈ N O₇ P | NEG | 1.164785416 | 0.147881613 | ↓ △△△ | 1.174011477 | 0.055244179 | ↓ *** |
| 83 | 2369 | Ne-(1-Carboxymethyl)-L-lysine | C₈ H₁₆ N₂ O₄ | POS | 1.160756915 | 0.149856743 | ↓ △△△ | 1.17088193 | 0.031858405 | ↓ *** |
| 84 | 10508 | Lysopc 18:2 | C₂₆ H₅₀ N O₇ P | NEG | 1.164410035 | 0.152309298 | ↓ △△△ | 1.173419921 | 0.022186362 | ↓ *** |
| 85 | 9968 | PC O-18:1 | C₂₆ H₅₂ N O₇ P | POS | 1.160776484 | 0.154033556 | ↓ △△△ | 1.16939007 | 0.151276759 | ↓ *** |
| 86 | 554 | Adipic acid | C₆ H₁₀ O₄ | NEG | 1.157402381 | 0.154576466 | ↓ △△△ | 1.15577988 | 2.318347049 | ↑ *** |
| 87 | 1879 | 4-(4-cyclohexylphenyl)-4-oxobut-2-enoic acid | C₁₆ H₁₈ O₃ | POS | 1.158378923 | 0.154799393 | ↓ △△△ | 1.167406677 | 4.079950017 | ↑ *** |
| 88 | 10957 | LPC O-17:0 | C₂₅ H₅₄ N O₆ P | NEG | 1.154512154 | 0.155058852 | ↓ △△△ | 1.135893358 | 0.303406996 | ↓ *** |
| 89 | 6256 | Hydrocortisone | C₂₁ H₃₀ O₅ | POS | 1.164716773 | 0.155904107 | ↓ △△△ | 1.170713926 | 0.220599276 | ↓ *** |
| 90 | 11035 | PG 4:0_14:0 | C₂₄ H₄₇ O₁₀ P | POS | 1.16510742 | 0.156160356 | ↓ △△△ | 1.173720689 | 0.162650868 | ↓ *** |
| 91 | 1571 | N-Acetyl-aspartic acid | C₆ H₉ N O₅ | POS | 1.045088684 | 0.156268961 | ↓ △△△ | 1.023823065 | 0.25948534 | ↓ *** |
| 92 | 9622 | (+/-)-CP 47,497-C7-Hydroxy metabolite | C₂₁ H₃₄ O₃ | NEG | 1.146726353 | 0.158102074 | ↓ △△△ | 1.006860618 | 0.466050009 | ↓ *** |
| 93 | 10252 | LPC 22:4-SN1 | C₃₀ H₅₄ N O₇ P | NEG | 1.129768598 | 0.158164491 | ↓ △△△ | 1.128505807 | 0.25009874 | ↓ *** |
| 94 | 5151 | L-Leucyl-L-Alanine | C₉ H₁₈ N₂ O₃ | POS | 1.165265101 | 0.159173257 | ↓ △△△ | 1.133161823 | 0.383384173 | ↓ *** |
| 95 | 6168 | Kahweol | C₂₀ H₂₆ O₃ | POS | 1.164771737 | 0.159628976 | ↓ △△△ | 1.168586873 | 0.419574885 | ↓ *** |
| 96 | 7584 | Thromboxane B3 | C₂₀ H₃₂ O₆ | POS | 1.140956986 | 0.15971478 | ↓ △△△ | 1.12122257 | 0.466446043 | ↓ *** |
| 97 | 9459 | PC O-18:2 | C₂₆ H₅₀ N O₇ P | NEG | 1.165682878 | 0.160269431 | ↓ △△△ | 1.174240801 | 0.14675677 | ↓ *** |
| 98 | 7055 | ent-Prostaglandin F2α | C₂₀ H₃₄ O₅ | NEG | 1.159940088 | 0.160277578 | ↓ △△△ | 1.169474052 | 0.44279099 | ↓ *** |
| 99 | 10408 | LPC 17:0-SN1 | C₂₅ H₅₂ N O₇ P | NEG | 1.14154221 | 0.162553281 | ↓ △△△ | 1.152878554 | 0.112711144 | ↓ *** |
| 100 | 10527 | PC O-18:0 | C₂₆ H₅₄ N O₇ P | NEG | 1.164135467 | 0.164295636 | ↓ △△△ | 1.173148113 | 0.066064304 | ↓ *** |
| 101 | 10835 | LPG 18:2 | C₂₄ H₄₅ O₉ P | NEG | 1.161721443 | 0.16517502 | ↓ △△△ | 1.170209171 | 0.17373508 | ↓ *** |
| 102 | 9028 | 8(S)-Hydroxy-(5Z,9E,11Z,14Z)-eicosatetraenoic acid | C₂₀ H₃₂ O₃ | POS | 1.152846776 | 0.166713008 | ↓ △△△ | 1.156697113 | 0.278986759 | ↓ *** |
| 103 | 9721 | 20-Hydroxy-(5Z,8Z,11Z,14Z)-eicosatetraenoic acid | C₂₀ H₃₂ O₃ | NEG | 1.149148683 | 0.167199809 | ↓ △△△ | 1.15153572 | 0.293310895 | ↓ *** |
| 104 | 8675 | 1,4-dihydroxy-1,4-dimethyl-7-(propan-2-ylidene)-decahydroazulen-6-one | C₁₅ H₂₄ O₃ | POS | 1.160805571 | 0.169061099 | ↓ △△△ | 1.162128122 | 0.352291496 | ↓ *** |
| 105 | 816 | (±)11(12)-DiHET | C₂₀ H₃₄ O₄ | POS | 1.162361383 | 0.16975655 | ↓ △△△ | 1.166955792 | 0.395940347 | ↓ *** |
| 106 | 8212 | (+/-)8(9)-DiHET | C₂₀ H₃₄ O₄ | POS | 1.165224697 | 0.170484463 | ↓ △△△ | 1.171848617 | 0.410311277 | ↓ *** |
| 107 | 9803 | LPC 17:1-SN1 | C₂₅ H₅₀ N O₇ P | POS | 1.148518511 | 0.172437985 | ↓ △△△ | 1.159918151 | 0.098516015 | ↓ *** |
| 108 | 9497 | U-44069 | C₂₁ H₃₄ O₄ | POS | 1.054893363 | 0.17365248 | ↓ △△△ | 1.063572057 | 0.169864401 | ↓ *** |
| 109 | 10178 | LPC 18:1-SN1 | C₂₆ H₅₂ N O₇ P | POS | 1.165433874 | 0.17544683 | ↓ △△△ | 1.173487533 | 0.250981429 | ↓ *** |
| 110 | 8589 | (±)8-HEPE | C₂₀ H₃₀ O₃ | NEG | 1.157851495 | 0.179068731 | ↓ △△△ | 1.084476526 | 0.48191871 | ↓ *** |
| 111 | 1528 | Methyl-beta-galactopyranoside | C₇ H₁₄ O₆ | NEG | 1.151070612 | 0.179461048 | ↓ △△△ | 1.138844492 | 0.325418056 | ↓ *** |
| 112 | 5105 | ALK | C₁₅ H₃₀ N₄ O₄ | NEG | 1.104895657 | 0.181603077 | ↓ △△△ | 1.064518597 | 0.379162583 | ↓ *** |
| 113 | 10749 | PC O-20:1 | C₂₈ H₅₆ N O₇ P | POS | 1.160488686 | 0.182573699 | ↓ △△△ | 1.170544043 | 0.107502004 | ↓ *** |
| 114 | 529 | 8-iso-15-keto Prostaglandin F2α | C₂₀ H₃₂ O₅ | NEG | 1.09046277 | 0.183449688 | ↓ △△△ | 1.003592202 | 0.480890834 | ↓ *** |
| 115 | 9414 | CAR 18:0 | C₂₅ H₅₀ N O₄ | NEG | 1.16527857 | 0.184365145 | ↓ △△△ | 1.174001492 | 0.121125582 | ↓ *** |
| 116 | 2419 | 4-methyl-6-[(4-methylphenyl)thio]-2-(2-pyridyl)pyrimidine | C₁₇ H₁₅ N₃ S | NEG | 1.151652086 | 0.184479212 | ↓ △△△ | 1.150977363 | 0.375747335 | ↓ *** |
| 117 | 9139 | (±)10(11)-EpDPA | C₂₂ H₃₂ O₃ | POS | 1.162010968 | 0.191440607 | ↓ △△△ | 1.168157146 | 0.297311751 | ↓ *** |
| 118 | 7605 | CAR 12:0 | C₁₉ H₃₈ N O₄ | POS | 1.165624913 | 0.192961822 | ↓ △△△ | 1.174300739 | 0.074202978 | ↓ *** |
| 119 | 9681 | LPG 14:0 | C₂₀ H₄₁ O₉ P | NEG | 1.165566244 | 0.192962119 | ↓ △△△ | 1.173725566 | 0.292568535 | ↓ *** |
| 120 | 7046 | Stercobilin | C₃₃ H₄₆ N₄ O₆ | POS | 1.133782071 | 0.198311802 | ↓ △△△ | 1.036284054 | 0.474453587 | ↓ *** |
| 121 | 9762 | PC O-16:0 | C₂₄ H₅₀ N O₇ P | POS | 1.164297838 | 0.200611662 | ↓ △△△ | 1.173218572 | 0.124247728 | ↓ *** |
| 122 | 8661 | Eicosapentaenoic acid | C₂₀ H₃₀ O₂ | POS | 1.163902116 | 0.201924047 | ↓ △△△ | 1.1714248 | 0.379452067 | ↓ *** |
| 123 | 10484 | LPC 20:2 | C₂₈ H₅₄ N O₇ P | NEG | 1.162254106 | 0.205774189 | ↓ △△△ | 1.170943232 | 0.266686336 | ↓ *** |
| 124 | 2362 | SNH | C₁₃ H₂₀ N₆ O₆ | NEG | 1.163489402 | 0.205799752 | ↓ △△△ | 1.116637756 | 3.44422349 | ↑ *** |
| 125 | 10303 | Docosahexaenoic acid | C₂₂ H₃₂ O₂ | NEG | 1.161178908 | 0.206064616 | ↓ △△△ | 1.171131823 | 0.13957783 | ↓ *** |
| 126 | 8801 | 11-Deoxy prostaglandin F1α | C₂₀ H₃₆ O₄ | NEG | 1.143983666 | 0.20614972 | ↓ △△△ | 1.096775322 | 0.394241077 | ↓ *** |
| 127 | 8827 | Palmitoylcarnitine | C₂₃ H₄₅ N O₄ | NEG | 1.165644543 | 0.207095532 | ↓ △△△ | 1.174324624 | 0.080745599 | ↓ *** |
| 128 | 11124 | FAHFA 18:1/3:0 | C₂₁ H₃₈ O₄ | POS | 1.151293102 | 0.215839989 | ↓ △△△ | 1.164014256 | 0.136997711 | ↓ *** |
| 129 | 9499 | LPE 20:4 | C₂₅ H₄₄ N O₇ P | NEG | 1.164830486 | 0.217235578 | ↓ △△△ | 1.173770746 | 0.097175402 | ↓ *** |
| 130 | 9924 | LPC O-15:1 | C₂₃ H₄₈ N O₆ P | POS | 1.157180036 | 0.217471518 | ↓ △△△ | 1.133589801 | 0.122043323 | ↓ *** |
| 131 | 9819 | 2-Arachidonoyl glycerol | C₂₃ H₃₈ O₄ | NEG | 1.161555451 | 0.220000805 | ↓ △△△ | 1.171684611 | 0.124166337 | ↓ *** |
| 132 | 10331 | LPI 20:4 | C₂₉ H₄₉ O₁₂ P | NEG | 1.164837015 | 0.2216799 | ↓ △△△ | 1.173790603 | 0.106124394 | ↓ *** |
| 133 | 6095 | 5,7-dihydroxy-3-(4-hydroxyphenyl)-4H-chromen-4-one | C₁₅ H₁₀ O₅ | NEG | 1.162657462 | 0.232724267 | ↓ △△△ | 1.173409763 | 2.946881354 | ↑ *** |
| 134 | 1845 | D-Glucono-1,5-lactone | C₆ H₁₀ O₆ | POS | 1.159938399 | 0.234180972 | ↓ △△△ | 1.16928269 | 5.768848149 | ↑ *** |
| 135 | 1885 | Guanosine monophosphate (GMP) | C₁₀ H₁₄ N₅ O₈ P | NEG | 1.115007419 | 0.238639104 | ↓ △△△ | 1.067686698 | 0.489456292 | ↓ *** |
| 136 | 8104 | 12(S)-HETE | C₂₀ H₃₂ O₃ | POS | 1.156101092 | 0.24322431 | ↓ △△△ | 1.159844154 | 0.378480031 | ↓ *** |
| 137 | 7058 | 15(R)-Prostaglandin D2 | C₂₀ H₃₂ O₅ | POS | 1.165890308 | 0.246259505 | ↓ △△△ | 1.174233714 | 0.324444325 | ↓ *** |
| 138 | 6552 | NSI-189 | C₂₂ H₃₀ N₄ O | POS | 1.144546272 | 0.248857282 | ↓ △△△ | 1.146767576 | 0.348553957 | ↓ *** |
| 139 | 1425 | Mycophenolic acid | C₁₇ H₂₀ O₆ | POS | 1.163507264 | 0.249281489 | ↓ △△△ | 1.168634073 | 2.952238651 | ↑ *** |
| 140 | 9583 | PC O-17:1 | C₂₅ H₅₀ N O₇ P | NEG | 1.146279833 | 0.255438558 | ↓ △△△ | 1.171172491 | 0.154259375 | ↓ *** |
| 141 | 6267 | ringenin | C₁₅ H₁₂ O₅ | POS | 1.164523621 | 0.265269024 | ↓ △△△ | 1.172850312 | 0.382875482 | ↓ *** |
| 142 | 9696 | LPE 22:5 | C₂₇ H₄₆ N O₇ P | POS | 1.122825076 | 0.265383502 | ↓ △△△ | 1.133232138 | 0.251228525 | ↓ *** |
| 143 | 10933 | LPG O-15:1 | C₂₁ H₄₃ O₈ P | POS | 1.165184158 | 0.267249365 | ↓ △△△ | 1.171940152 | 0.478195446 | ↓ *** |
| 144 | 4204 | 3-Methylglutaric acid | C₆ H₁₀ O₄ | POS | 1.163787968 | 0.272714767 | ↓ △△△ | 1.170963812 | 0.465853354 | ↓ *** |
| 145 | 10239 | LPE 17:0 | C₂₂ H₄₆ N O₇ P | NEG | 1.078237879 | 0.274292102 | ↓ △△△ | 1.039810624 | 0.402717934 | ↓ *** |
| 146 | 1644 | Orotic acid | C₅ H₄ N₂ O₄ | POS | 1.127308012 | 0.275676551 | ↓ △△△ | 1.118426707 | 0.44133712 | ↓ *** |
| 147 | 8863 | 16(R)-HETE | C₂₀ H₃₂ O₃ | NEG | 1.152735395 | 0.2801875 | ↓ △△△ | 1.148025363 | 0.480021943 | ↓ *** |
| 148 | 8637 | CAR 16:1 | C₂₃ H₄₄ N O₄ | POS | 1.153661436 | 0.283237995 | ↓ △△△ | 1.168094881 | 0.066172479 | ↓ *** |
| 149 | 10875 | 8Z,11Z,14Z-Eicosatrienoic acid | C₂₀ H₃₄ O₂ | NEG | 1.151328924 | 0.296690117 | ↓ △△△ | 1.167679851 | 0.058802477 | ↓ *** |
| 150 | 5529 | Reduced glutathione | C₁₀ H₁₇ N₃ O₆ S | NEG | 1.127243592 | 0.305027863 | ↓ △△△ | 1.067547133 | 0.453455425 | ↓ *** |
| 151 | 5011 | Maltotriose | C₁₈ H₃₂ O₁₆ | NEG | 1.162848043 | 0.307274602 | ↓ △△△ | 1.171737311 | 4.702055061 | ↑ *** |
| 152 | 10371 | Arachidonic acid | C₂₀ H₃₂ O₂ | POS | 1.159118743 | 0.322465168 | ↓ △△△ | 1.17082967 | 0.148580166 | ↓ *** |
| 153 | 6843 | Andrographolide | C₂₀ H₃₀ O₅ | NEG | 1.154041609 | 0.326594905 | ↓ △△△ | 1.173349542 | 0.473832917 | ↓ *** |
| 154 | 9679 | Docosahexaenoyl Ethanolamide | C₂₄ H₃₇ N O₂ | NEG | 1.165011584 | 0.332929905 | ↓ △△△ | 1.174333976 | 0.103528847 | ↓ *** |
| 155 | 5635 | 7-Hydroxy-3,4-dihydrocarbostyril | C₉ H₉ N O₂ | POS | 1.045083721 | 0.336305785 | ↓ △△△ | 1.171006931 | 2.030354758 | ↑ *** |
| 156 | 7924 | Estradiol Benzoate | C₂₅ H₂₈ O₃ | NEG | 1.127950436 | 0.338796568 | ↓ △△△ | 1.172597957 | 3.824387751 | ↑ *** |
| 157 | 627 | Daidzein | C₁₅ H₁₀ O₄ | NEG | 1.165468954 | 0.340813054 | ↓ △△△ | 1.174549092 | 5.028245221 | ↑ *** |
| 158 | 1482 | Stachyose | C₂₄ H₄₂ O₂₁ | NEG | 1.163649109 | 0.343628342 | ↓ △△△ | 1.171019444 | 4.799115642 | ↑ *** |
| 159 | 1817 | 3'-Adenosine monophosphate (3'-AMP) | C₁₀ H₁₄ N₅ O₇ P | POS | 1.091464818 | 0.346520525 | ↓ △△△ | 1.16945925 | 0.20355603 | ↓ *** |
| 160 | 5061 | Trehalose | C₁₂ H₂₂ O₁₁ | POS | 1.150371523 | 0.348565906 | ↓ △△△ | 1.150439777 | 3.170571497 | ↑ *** |
| 161 | 1444 | Oxytetracycline | C₂₂ H₂₄ N₂ O₉ | NEG | 1.098899909 | 0.348880043 | ↓ △△△ | 1.145673625 | 3.590064626 | ↑ *** |
| 162 | 9769 | LPE O-15:1 | C₂₀ H₄₂ N O₆ P | NEG | 1.108860874 | 0.352292829 | ↓ △△△ | 1.169642279 | 0.246121401 | ↓ *** |
| 163 | 1924 | α,α-Trehalose | C₁₂ H₂₂ O₁₁ | POS | 1.163446468 | 0.35471549 | ↓ △△△ | 1.172715379 | 3.427664034 | ↑ *** |
| 164 | 10839 | FAHFA 16:0/18:2 | C₃₄ H₆₂ O₄ | NEG | 1.123879243 | 0.354812 | ↓ △△△ | 1.163109229 | 3.047362372 | ↑ *** |
| 165 | 206 | Gluconolactone | C₆ H₁₀ O₆ | NEG | 1.158826594 | 0.357733242 | ↓ △△△ | 1.168779354 | 2.411751565 | ↑ *** |
| 166 | 9408 | (+/-)13-HODE | C₁₈ H₃₂ O₃ | POS | 1.162106017 | 0.358045415 | ↓ △△△ | 1.170605389 | 0.384808983 | ↓ *** |
| 167 | 824 | CAR 20:5 | C₂₇ H₄₄ N O₄ | POS | 1.125223936 | 0.361373045 | ↓ △△△ | 1.173896755 | 0.113766829 | ↓ *** |
| 168 | 10181 | Palmitoleic Acid | C₁₆ H₃₀ O₂ | NEG | 1.15586569 | 0.366441301 | ↓ △△△ | 1.16279385 | 0.428317075 | ↓ *** |
| 169 | 8597 | CAR 18:2 | C₂₅ H₄₆ N O₄ | NEG | 1.159323433 | 0.366495623 | ↓ △△△ | 1.16579644 | 0.099364096 | ↓ *** |
| 170 | 9993 | 16-Hydroxyhexadecanoic acid | C₁₆ H₃₂ O₃ | NEG | 1.164601272 | 0.376599186 | ↓ △△△ | 1.173986909 | 0.234435974 | ↓ *** |
| 171 | 9071 | Lysopg 18:1 | C₂₄ H₄₇ O₉ P | NEG | 1.161719776 | 0.383296049 | ↓ △△△ | 1.171586816 | 0.356712528 | ↓ *** |
| 172 | 6638 | Isorhapontigenin | C₁₅ H₁₄ O₄ | POS | 1.14786468 | 0.384673301 | ↓ △△△ | 1.171195146 | 5.108890489 | ↑ *** |
| 173 | 3417 | 3-Hydroxy-3-methylglutaric acid | C₆ H₁₀ O₅ | POS | 1.161151169 | 0.38751292 | ↓ △△△ | 1.099645449 | 2.464348866 | ↑ *** |
| 174 | 8935 | FAHFA 2:0/18:1 | C₂₀ H₃₆ O₄ | POS | 1.13110317 | 0.39107215 | ↓ △△△ | 1.146197146 | 0.312419059 | ↓ *** |
| 175 | 9181 | LPC 18:3 | C₂₆ H₄₈ N O₇ P | POS | 1.104126847 | 0.397608272 | ↓ △△△ | 1.166873006 | 3.02216906 | ↑ *** |
| 176 | 5128 | FMH | C₂₀ H₂₇ N₅ O₄ S | NEG | 1.163633048 | 0.400331096 | ↓ △△△ | 1.171436674 | 0.489103371 | ↓ *** |
| 177 | 7105 | Taurochenodeoxycholic acid | C₂₆ H₄₅ N O₆ S | POS | 1.160083733 | 0.400666865 | ↓ △△△ | 1.169627787 | 0.42661746 | ↓ *** |
| 178 | 5649 | Glu-Val-Phe | C₁₉ H₂₇ N₃ O₆ | NEG | 1.116891195 | 0.408239154 | ↓ △△△ | 1.102712682 | 0.490466637 | ↓ *** |
| 179 | 807 | Sphingosine (d18:1) | C₁₈ H₃₇ N O₂ | NEG | 1.16169276 | 0.408796044 | ↓ △△△ | 1.1731225 | 2.373104055 | ↑ *** |
| 180 | 1499 | Cytidine-5'-monophosphate | C₉ H₁₄ N₃ O₈ P | NEG | 1.098000952 | 0.413115863 | ↓ △△△ | 1.131083318 | 0.276352803 | ↓ *** |
| 181 | 10355 | Lithocholic acid | C₂₄ H₄₀ O₃ | POS | 1.150867794 | 0.418991748 | ↓ △△△ | 1.169335075 | 0.063401262 | ↓ *** |
| 182 | 8553 | CAR 20:4 | C₂₇ H₄₆ N O₄ | NEG | 1.155010559 | 0.421742891 | ↓ △△△ | 1.170550335 | 0.049163041 | ↓ *** |
| 183 | 8983 | CAR 18:1 | C₂₅ H₄₈ N O₄ | NEG | 1.165620393 | 0.424010495 | ↓ △△△ | 1.174681375 | 0.073555854 | ↓ *** |
| 184 | 9541 | CAR 20:1 | C₂₇ H₅₂ N O₄ | NEG | 1.160471469 | 0.430241567 | ↓ △△△ | 1.172199138 | 0.135202174 | ↓ *** |
| 185 | 1337 | O-Phospho-L-serine | C₃ H₈ N O₆ P | POS | 1.117521668 | 0.431457163 | ↓ △△△ | 1.157985207 | 0.258473324 | ↓ *** |
| 186 | 1572 | Kojic acid | C₆ H₆ O₄ | POS | 1.113721302 | 0.432540009 | ↓ △△△ | 1.023407802 | 2.246486054 | ↑ *** |
| 187 | 5641 | 2-Hydroxy-2-methylbutanedioic acid | C₅ H₈ O₅ | NEG | 1.137865362 | 0.438122629 | ↓ △△△ | 1.156418722 | 2.220133446 | ↑ *** |
| 188 | 1506 | Glu-Glu | C₁₀ H₁₆ N₂ O₇ | NEG | 1.141903507 | 0.442811382 | ↓ △△△ | 1.143681836 | 0.486866517 | ↓ *** |
| 189 | 1473 | D-Raffinose | C₁₈ H₃₂ O₁₆ | NEG | 1.154475294 | 0.443938185 | ↓ △△△ | 1.164879963 | 4.839427748 | ↑ *** |
| 190 | 8241 | trans-Petroselinic Acid | C₁₈ H₃₄ O₂ | POS | 1.080765586 | 0.445159974 | ↓ △△△ | 1.169483102 | 0.248898046 | ↓ *** |
| 191 | 1479 | 3-(2-thienyl)-1,2,4-oxadiazole-5-carbohydrazide | C₇ H₆ N₄ O₂ S | NEG | 1.073203308 | 0.450591194 | ↓ △△△ | 1.169619582 | 3.67334019 | ↑ *** |
| 192 | 866 | CAR 17:1 | C₂₄ H₄₆ N O₄ | NEG | 1.038711968 | 0.45453839 | ↓ △△△ | 1.146148842 | 0.077053264 | ↓ *** |
| 193 | 239 | PPK | C₁₆ H₂₈ N₄ O₄ | POS | 1.156687653 | 0.455351191 | ↓ △△△ | 1.170807191 | 5.566105832 | ↑ *** |
| 194 | 8564 | 16-Heptadecyne-1,2,4-triol | C₁₇ H₃₂ O₃ | POS | 1.062524545 | 0.456835807 | ↓ △△△ | 1.127288315 | 0.456572616 | ↓ *** |
| 195 | 8168 | Lauric acid ethyl ester | C₁₄ H₂₈ O₂ | POS | 1.11963336 | 0.462882731 | ↓ △△△ | 1.126076269 | 0.496000529 | ↓ *** |
| 196 | 5903 | Corey Lactone Diol | C₈ H₁₂ O₄ | POS | 1.139831498 | 0.462946242 | ↓ △△△ | 1.148364098 | 0.462677514 | ↓ *** |
| 197 | 2625 | L-beta-Imidazolelactic acid | C₆ H₈ N₂ O₃ | NEG | 1.159787859 | 0.464506803 | ↓ △△△ | 1.16548552 | 0.491122945 | ↓ *** |
| 198 | 2123 | Ethylmalonic acid | C₅ H₈ O₄ | POS | 1.159554547 | 0.465052808 | ↓ △△△ | 1.168767915 | 2.015243518 | ↑ *** |
| 199 | 9752 | LPC 22:5 | C₃₀ H₅₂ N O₇ P | NEG | 1.127230784 | 0.477531145 | ↓ △△△ | 1.143300282 | 0.368365688 | ↓ *** |
| 200 | 6162 | Nonoic acid | C₉ H₁₈ O₂ | NEG | 1.162217466 | 0.481819487 | ↓ △△△ | 1.168897777 | 2.03562214 | ↑ *** |
| 201 | 5248 | 5-(3-chloro-4-methylanilino)-1-methyl-1H-pyrazol-3-ol | C₁₁ H₁₂ Cl N₃ O | NEG | 1.098834002 | 0.484324079 | ↓ △△△ | 1.105658861 | 0.490895168 | ↓ *** |
| 202 | 5849 | 5-Hydroxyindole-3-acetic acid | C₁₀ H₉ N O₃ | NEG | 1.16324224 | 0.491320459 | ↓ △△△ | 1.172278302 | 2.408646007 | ↑ *** |
| 203 | 6579 | Lysope 14:0 | C₁₉ H₄₀ N O₇ P | POS | 1.142573405 | 2.065650981 | ↑ △△△ | 1.14535821 | 2.009128642 | ↑ *** |
| 204 | 7374 | β-Muricholic acid | C₂₄ H₄₀ O₅ | NEG | 1.155827417 | 2.108470926 | ↑ △△△ | 1.172453762 | 3.578535067 | ↑ *** |
| 205 | 1459 | JNJ-1661010 | C₁₉ H₁₉ N₅ O S | NEG | 1.138495081 | 2.160767791 | ↑ △△△ | 1.160788012 | 2.333261301 | ↑ *** |
| 206 | 3873 | dAMP | C₁₀ H₁₄ N₅ O₆ P | NEG | 1.164554366 | 2.205766815 | ↑ △△△ | 1.169843228 | 3.030481896 | ↑ *** |
| 207 | 1437 | N-Acetyl-D-lactosamine | C₁₄ H₂₅ N O₁₁ | NEG | 1.150187968 | 2.250791437 | ↑ △△△ | 1.165982415 | 2.00719939 | ↑ *** |
| 208 | 9732 | Andamide (AEA) | C₂₂ H₃₇ N O₂ | POS | 1.161495239 | 2.338685222 | ↑ △△△ | 1.171368196 | 0.060949737 | ↓ *** |
| 209 | 10477 | FAHFA 18:2/20:4 | C₃₈ H₆₂ O₄ | POS | 1.119716822 | 2.366193399 | ↑ △△△ | 1.146268396 | 2.469877418 | ↑ *** |
| 210 | 8744 | 1a,1b-Dihomo prostaglandin E1 | C₂₂ H₃₈ O₅ | POS | 1.150576272 | 2.47151921 | ↑ △△△ | 1.147262598 | 0.378041821 | ↓ *** |
| 211 | 1052 | 2-hydroxy-6-[(8Z,11Z)-pentadeca-8,11,14-trien-1-yl]benzoic acid | C₂₂ H₃₀ O₃ | NEG | 1.1309038 | 2.593899571 | ↑ △△△ | 1.123652805 | 2.343711936 | ↑ *** |
| 212 | 4861 | Guanosine | C₁₀ H₁₃ N₅ O₅ | POS | 1.153031845 | 2.62381992 | ↑ △△△ | 1.15493014 | 2.066318601 | ↑ *** |
| 213 | 5618 | 4-Toluenesulfonic acid | C₇ H₈ O₃ S | POS | 1.160833875 | 2.663845375 | ↑ △△△ | 1.171087497 | 9.039083189 | ↑ *** |
| 214 | 6286 | 3-amino-2-phenyl-2H-pyrazolo[4,3-c]pyridine-4,6-diol | C₁₂ H₁₀ N₄ O₂ | NEG | 1.165633138 | 2.75606022 | ↑ △△△ | 1.173531451 | 0.247628028 | ↓ *** |
| 215 | 5339 | Esculin | C₁₅ H₁₆ O₉ | NEG | 1.152178261 | 2.768741423 | ↑ △△△ | 1.098748991 | 2.234747377 | ↑ *** |
| 216 | 7009 | Hexadecanedioic acid | C₁₆ H₃₀ O₄ | POS | 1.152528867 | 2.885154019 | ↑ △△△ | 1.157759398 | 2.694605356 | ↑ *** |
| 217 | 7104 | 1-(3-phenylpropanoyl)-4-piperidinecarboxylic acid | C₁₅ H₁₉ N O₃ | NEG | 1.14587823 | 3.149167475 | ↑ △△△ | 1.102774672 | 2.468001733 | ↑ *** |
| 218 | 8353 | 1-methyl-3-phenyl-1H-pyrazol-5-amine | C₁₀ H₁₁ N₃ | POS | 1.158184857 | 3.26564966 | ↑ △△△ | 1.148688259 | 2.221488647 | ↑ *** |
| 219 | 8705 | PE O-16:1_22:4 | C₄₃ H₇₈ N O₇ P | NEG | 1.162034009 | 3.27215215 | ↑ △△△ | 1.090324357 | 2.717740358 | ↑ *** |
| 220 | 1837 | Cyclic ADP-ribose | C₁₅ H₂₁ N₅ O₁₃ P₂ | POS | 1.13398209 | 3.324430267 | ↑ △△△ | 1.139177886 | 6.171730021 | ↑ *** |
| 221 | 5974 | Phenylacetaldehyde | C₈ H₈ O | POS | 1.160925316 | 3.411110352 | ↑ △△△ | 1.136561614 | 3.949182223 | ↑ *** |
| 222 | 6013 | 5,6-dimethoxy-2-(2-methoxyphenyl)-4H-chromen-4-one | C₁₈ H₁₆ O₅ | POS | 1.165773849 | 3.646298503 | ↑ △△△ | 1.16813425 | 2.785936197 | ↑ *** |
| 223 | 6012 | 2-Methoxybenzaldehyde | C₈ H₈ O₂ | POS | 1.165773849 | 3.646298503 | ↑ △△△ | 1.16813425 | 2.785936197 | ↑ *** |
| 224 | 5095 | 2'-Deoxyinosine | C₁₀ H₁₂ N₄ O₄ | POS | 1.15172293 | 3.69893746 | ↑ △△△ | 1.172189499 | 3.333726211 | ↑ *** |
| 225 | 5091 | Deoxyinosine | C₁₀ H₁₂ N₄ O₄ | NEG | 1.151984731 | 3.944242728 | ↑ △△△ | 1.15953193 | 2.267169281 | ↑ *** |
| 226 | 5278 | N6-Methyladenine | C₆ H₇ N₅ | POS | 1.163840226 | 4.220536957 | ↑ △△△ | 1.140070569 | 2.002575482 | ↑ *** |
| 227 | 81 | Glycodeoxycholic acid | C₂₆ H₄₃ N O₅ | NEG | 1.155302774 | 4.468583235 | ↑ △△△ | 1.096723179 | 2.14566455 | ↑ *** |
| 228 | 10117 | Ursolic acid | C₃₀ H₄₈ O₃ | POS | 1.161072969 | 4.589525123 | ↑ △△△ | 1.167490681 | 2.906830544 | ↑ *** |
| 229 | 4015 | 2-Deoxyuridine | C₉ H₁₂ N₂ O₅ | POS | 1.154930695 | 4.666352927 | ↑ △△△ | 1.142011819 | 2.643324711 | ↑ *** |
| 230 | 8369 | 5-[(10Z)-14-(3,5-dihydroxyphenyl)tetradec-10-en-1-yl]benzene-1,3-diol | C₂₆ H₃₆ O₄ | POS | 1.163275066 | 4.838249844 | ↑ △△△ | 1.170832025 | 2.01501566 | ↑ *** |
| 231 | 6985 | Sodium cholate | C₂₄ H₃₉ O₅ | POS | 1.151140413 | 5.143912788 | ↑ △△△ | 1.092331813 | 2.232978366 | ↑ *** |
| 232 | 5617 | (3-Methoxy-4-hydroxyphenyl)ethylene glycol sulfate | C₉ H₁₂ O₇ S | NEG | 1.164174377 | 5.164253182 | ↑ △△△ | 1.138577671 | 2.228497137 | ↑ *** |
| 233 | 7655 | 2,4-dihydroxyheptadec-16-en-1-yl acetate | C₁₉ H₃₆ O₄ | POS | 1.162752287 | 5.427509451 | ↑ △△△ | 1.165999148 | 2.812818996 | ↑ *** |
| 234 | 1835 | L-Methionine sulfone | C₅ H₁₁ N O₄ S | POS | 1.164220708 | 6.157361376 | ↑ △△△ | 1.141078244 | 2.021192786 | ↑ *** |
| 235 | 9084 | 23-Nordeoxycholic acid | C₂₃ H₃₈ O₄ | NEG | 1.163896434 | 6.485012844 | ↑ △△△ | 1.163834854 | 0.412311395 | ↓ *** |
| 236 | 8862 | cis-7-Hexadecenoic Acid | C₁₆ H₃₀ O₂ | NEG | 1.165264295 | 6.619668489 | ↑ △△△ | 1.15055118 | 2.482195411 | ↑ *** |
| 237 | 2649 | Uridine | C₉ H₁₂ N₂ O₆ | NEG | 1.144848175 | 9.349842207 | ↑ △△△ | 1.091761785 | 6.768374842 | ↑ *** |
| 238 | 6816 | Glycocholic acid | C₂₆ H₄₃ N O₆ | POS | 1.161029144 | 40.93484813 | ↑ △△△ | 1.167286383 | 8.917803483 | ↑ *** |
| 239 | 8868 | 9-Oxo-10(E),12(E)-octadecadienoic acid | C₁₈ H₃₀ O₃ | NEG | 1.050844286 | 97.51205054 | ↑ △△△ | 1.170243278 | 73.27929984 | ↑ *** |

^△^*P*<0.05，^△△^*P*<0.01，^△△△^*P*<0.001 vs. the CON group ，^*^*P*<0.05，^**^*P*<0.01， ^***^*P*<0.001 vs. the CUMS group

**Table S6 KEGG enrichment analysis on the differential metabolite data between CON and CUMS groups**

| **Pathway** | **Gene Ratio** | **Bg Ratio** | **p value** | **p.adjust** | **q value** | **Gene Name** |
| --- | --- | --- | --- | --- | --- | --- |
| Aldosterone synthesis and secretion | 6/84 | 7/239 | 0.008349841 | 0.68756553 | 0.68756553 | AMP/Aldosterone/Arachidonate/Progesterone/12(S)-HETE/11-Deoxycorticosterone |
| Arachidonic acid metabolism | 9/84 | 13/239 | 0.010911688 | 0.68756553 | 0.68756553 | 8(S)-HETE/15(S)-HPETE/20-HETE/Prostaglandin B2/Arachidonate/20-COOH-Leukotriene B4/Prostaglandin E2/16(R)-HETE/12(S)-HETE |
| Cortisol synthesis and secretion | 3/84 | 3/239 | 0.042408023 | 0.68756553 | 0.68756553 | AMP/Cortisol/Progesterone |
| Cushing syndrome | 3/84 | 3/239 | 0.042408023 | 0.68756553 | 0.68756553 | AMP/Cortisol/Progesterone |
| Parathyroid hormone synthesis, secretion and action | 3/84 | 3/239 | 0.042408023 | 0.68756553 | 0.68756553 | AMP/Calcitriol/Calcidiol |
| Vascular smooth muscle contraction | 3/84 | 3/239 | 0.042408023 | 0.68756553 | 0.68756553 | 20-HETE/Arachidonate/Adenosine |

**Table S7 KEGG enrichment analysis on the differential metabolite between CUMS and H-SCFs groups**

| **Pathway** | **Gene Ratio** | **Bg Ratio** | **p value** | **p.adjust** | **q value** | **Gene Name** |
| --- | --- | --- | --- | --- | --- | --- |
| Taurine and hypotaurine metabolism | 4/68 | 4/239 | 0.006143358 | 0.436216222 | 0.436216222 | Taurine/3-Sulfino-L-alanine/Taurocholate/L-Glutamate |
| Biosynthesis of unsaturated fatty acids | 6/68 | 8/239 | 0.007520969 | 0.436216222 | 0.436216222 | (4Z,7Z,10Z,13Z,16Z,19Z)-Docosahexaenoic acid/Adrenic acid/Dihomo-gamma-linolenate/Arachidonate/(5Z,8Z,11Z,14Z,17Z)-Icosapentaenoic acid/Hexadecanoic acid |
| Primary bile acid biosynthesis | 3/68 | 3/239 | 0.022305114 | 0.733426313 | 0.733426313 | Glycocholate/Taurine/Taurocholate |

**Table S8.1 Correlations between potential microbial biomarkers and depressive-like behaviors involving the anti-depression effects of SCFs**

|  | **Bacteroidota** | | **Firmicutes** | | **Proteobacteria** | | **Actinobacteriota** | | **Actinobacteria** | | **Erysipelotrichaceae** | | **Lactobacillus** | | **Dubosiella** | | **Bacteroides** | | **Limosilactobacillus** | |
| --- | --- | --- | --- | --- | --- | --- | --- | --- | --- | --- | --- | --- | --- | --- | --- | --- | --- | --- | --- | --- |
|  | p | r | p | r | p | r | p | r | p | r | p | r | p | r | p | r | p | r | p | r |
| **Bacteroidota** | NA | 1.00 | 0.00 | -0.77 | 0.17 | -0.31 | 0.03 | -0.70 | 0.10 | -0.51 | 0.02 | -0.64 | 0.00 | -0.86 | 0.03 | -0.68 | 0.05 | 0.37 | 0.04 | -0.62 |
| **Firmicutes** | 0.00 | -0.77 | NA | 1.00 | 0.69 | -0.16 | 0.79 | 0.32 | 0.78 | 0.05 | 0.11 | 0.56 | 0.02 | 0.65 | 0.18 | 0.64 | 0.30 | -0.12 | 0.00 | 0.65 |
| **Proteobacteria** | 0.17 | -0.31 | 0.69 | -0.16 | NA | 1.00 | 0.00 | 0.40 | 0.00 | 0.55 | 0.14 | 0.20 | 0.29 | 0.39 | 0.04 | 0.20 | 0.37 | -0.33 | 0.70 | -0.04 |
| **Actinobacteriota** | 0.03 | -0.70 | 0.79 | 0.32 | 0.00 | 0.40 | NA | 1.00 | 0.00 | 0.69 | 0.07 | 0.44 | 0.47 | 0.61 | 0.01 | 0.52 | 0.26 | -0.35 | 0.83 | 0.46 |
| **Actinobacteria** | 0.10 | -0.51 | 0.78 | 0.05 | 0.00 | 0.55 | 0.00 | 0.69 | NA | 1.00 | 0.26 | 0.44 | 0.44 | 0.62 | 0.12 | 0.39 | 0.27 | -0.52 | 0.60 | 0.41 |
| **Erysipelotrichaceae** | 0.02 | -0.64 | 0.11 | 0.56 | 0.14 | 0.20 | 0.07 | 0.44 | 0.26 | 0.44 | NA | 1.00 | 0.06 | 0.62 | 0.00 | 0.87 | 0.29 | -0.55 | 0.85 | 0.40 |
| **Lactobacillus** | 0.00 | -0.86 | 0.02 | 0.65 | 0.29 | 0.39 | 0.47 | 0.61 | 0.44 | 0.62 | 0.06 | 0.62 | NA | 1.00 | 0.17 | 0.57 | 0.23 | -0.36 | 0.22 | 0.71 |
| **Dubosiella** | 0.03 | -0.68 | 0.18 | 0.64 | 0.04 | 0.20 | 0.01 | 0.52 | 0.12 | 0.39 | 0.00 | 0.87 | 0.17 | 0.57 | NA | 1.00 | 0.40 | -0.35 | 0.97 | 0.40 |
| **Bacteroides** | 0.05 | 0.37 | 0.30 | -0.12 | 0.37 | -0.33 | 0.26 | -0.35 | 0.27 | -0.52 | 0.29 | -0.55 | 0.23 | -0.36 | 0.40 | -0.35 | NA | 1.00 | 0.49 | -0.05 |
| **Body weight** | 0.00 | -0.58 | 0.03 | 0.44 | 0.24 | 0.11 | 0.15 | 0.56 | 0.15 | 0.48 | 0.40 | 0.41 | 0.11 | 0.48 | 0.51 | 0.43 | 0.02 | -0.44 | 0.04 | 0.68 |
| **Food intake** | 0.01 | -0.44 | 0.10 | 0.22 | 0.45 | 0.06 | 0.30 | 0.22 | 0.18 | 0.39 | 0.27 | 0.20 | 0.08 | 0.36 | 0.43 | 0.11 | 0.00 | -0.41 | 0.09 | 0.48 |
| **SPT** | 0.00 | -0.53 | 0.04 | 0.35 | 0.22 | 0.12 | 0.15 | 0.42 | 0.08 | 0.60 | 0.29 | 0.47 | 0.09 | 0.49 | 0.41 | 0.43 | 0.02 | -0.44 | 0.05 | 0.65 |
| **OFT- total distance** | 0.00 | -0.71 | 0.06 | 0.58 | 0.24 | 0.31 | 0.13 | 0.56 | 0.13 | 0.57 | 0.04 | 0.77 | 0.01 | 0.76 | 0.07 | 0.75 | 0.00 | -0.67 | 0.16 | 0.57 |
| **OFT-central residence times** | 0.21 | -0.46 | 0.11 | 0.30 | 0.91 | -0.03 | 0.97 | 0.40 | 0.92 | 0.46 | 0.95 | 0.43 | 0.79 | 0.40 | 0.66 | 0.37 | 0.11 | -0.50 | 0.13 | 0.47 |
| **TST** | 0.01 | 0.48 | 0.03 | -0.40 | 0.48 | -0.03 | 0.42 | -0.35 | 0.28 | -0.53 | 0.50 | -0.49 | 0.08 | -0.58 | 0.73 | -0.36 | 0.03 | 0.48 | 0.03 | -0.71 |
| **FST** | 0.00 | 0.61 | 0.01 | -0.50 | 0.32 | -0.12 | 0.29 | -0.47 | 0.24 | -0.51 | 0.45 | -0.46 | 0.05 | -0.65 | 0.64 | -0.34 | 0.02 | 0.55 | 0.03 | -0.73 |

**Table S8.2 Correlations between potential differential metabolites and depressive-like behaviors involving the anti-depression effects of SCFs**

|  | **Adenosine 5'-monophosphate(AMP)** | | **Aldosterone** | | **Taurochenodeoxycholic acid** | | **Tetrahydrocortisone** | | **Taurocholic acid** | | **Glycocholic acid** | | **Arachidonic acid** | | **12(S)-HETE** | | **15(S)-HpETE** | | **20-Hydroxy-(5Z,8Z,11Z,14Z)-eicosatetraenoic acid** | | **8(S)-Hydroxy-(5Z,9E,11Z,14Z)-eicosatetraenoic acid** | |
| --- | --- | --- | --- | --- | --- | --- | --- | --- | --- | --- | --- | --- | --- | --- | --- | --- | --- | --- | --- | --- | --- | --- |
|  | p | r | p | r | p | r | p | r | p | r | p | r | p | r | p | r | p | r | p | r | p | r |
| **Body weight** | 0.00 | -0.91 | 0.00 | -0.91 | 0.00 | -0.83 | 0.00 | -0.89 | 0.00 | -0.92 | 0.00 | 0.90 | 0.00 | -0.43 | 0.00 | -0.91 | 0.00 | -0.90 | 0.00 | -0.88 | 0.00 | -0.92 |
| **Food intake** | 0.00 | -0.62 | 0.00 | -0.60 | 0.00 | -0.55 | 0.00 | -0.61 | 0.00 | -0.69 | 0.01 | 0.73 | 0.00 | -0.14 | 0.00 | -0.63 | 0.00 | -0.64 | 0.00 | -0.76 | 0.00 | -0.77 |
| **SPT** | 0.00 | -0.88 | 0.00 | -0.83 | 0.00 | -0.77 | 0.00 | -0.91 | 0.00 | -0.93 | 0.00 | 0.88 | 0.00 | -0.45 | 0.00 | -0.90 | 0.00 | -0.88 | 0.00 | -0.93 | 0.00 | -0.92 |
| **OFT- total distance** | 0.00 | -0.66 | 0.00 | -0.71 | 0.00 | -0.70 | 0.00 | -0.64 | 0.00 | -0.61 | 0.01 | 0.72 | 0.00 | -0.62 | 0.00 | -0.62 | 0.00 | -0.71 | 0.00 | -0.58 | 0.00 | -0.67 |
| **OFT-central residence times** | 0.00 | -0.69 | 0.00 | -0.71 | 0.00 | -0.69 | 0.00 | -0.80 | 0.00 | -0.80 | 0.00 | 0.70 | 0.02 | -0.50 | 0.00 | -0.79 | 0.00 | -0.72 | 0.00 | -0.84 | 0.00 | -0.78 |
| **TST** | 0.00 | 0.88 | 0.00 | 0.86 | 0.00 | 0.81 | 0.00 | 0.87 | 0.00 | 0.87 | 0.00 | -0.85 | 0.00 | 0.40 | 0.00 | 0.88 | 0.00 | 0.86 | 0.00 | 0.90 | 0.00 | 0.84 |
| **FST** | 0.00 | 0.89 | 0.00 | 0.88 | 0.00 | 0.87 | 0.00 | 0.87 | 0.00 | 0.85 | 0.00 | -0.93 | 0.00 | 0.47 | 0.00 | 0.87 | 0.00 | 0.87 | 0.00 | 0.83 | 0.00 | 0.86 |

**Table S8.3 Correlations between potential microbial biomarkers and differential**

**metabolites involving the anti-depression effects of SCFs**

|  | **Bacteroidota** | | **Firmicutes** | | **Proteobacteria** | | **Actinobacteriota** | | **Actinobacteria** | | **Erysipelotrichaceae** | | **Lactobacillus** | | **Dubosiella** | | **Bacteroides** | | **Limosilactobacillus** | |
| --- | --- | --- | --- | --- | --- | --- | --- | --- | --- | --- | --- | --- | --- | --- | --- | --- | --- | --- | --- | --- |
|  | p | r | p | r | p | r | p | r | p | r | p | r | p | r | p | r | p | r | p | r |
| **Adenosine 5'-monophosphate(AMP)** | 0.00 | 0.62 | 0.02 | -0.48 | 0.34 | -0.12 | 0.17 | -0.57 | 0.13 | -0.51 | 0.28 | -0.43 | 0.05 | -0.60 | 0.42 | -0.38 | 0.04 | 0.41 | 0.04 | -0.77 |
| **Aldosterone** | 0.00 | 0.72 | 0.01 | -0.65 | 0.34 | -0.11 | 0.23 | -0.52 | 0.16 | -0.44 | 0.30 | -0.47 | 0.03 | -0.71 | 0.47 | -0.53 | 0.02 | 0.32 | 0.03 | -0.80 |
| **Taurochenodeoxycholic acid** | 0.00 | 0.39 | 0.03 | -0.22 | 0.31 | -0.21 | 0.17 | -0.50 | 0.09 | -0.55 | 0.18 | -0.38 | 0.03 | -0.46 | 0.32 | -0.31 | 0.00 | 0.65 | 0.07 | -0.52 |
| **Tetrahydrocortisone** | 0.00 | 0.65 | 0.01 | -0.54 | 0.34 | -0.11 | 0.17 | -0.52 | 0.10 | -0.53 | 0.17 | -0.58 | 0.03 | -0.60 | 0.31 | -0.57 | 0.01 | 0.40 | 0.05 | -0.69 |
| **Taurocholic acid** | 0.01 | 0.53 | 0.02 | -0.45 | 0.25 | -0.16 | 0.37 | -0.35 | 0.35 | -0.43 | 0.44 | -0.46 | 0.08 | -0.46 | 0.57 | -0.43 | 0.02 | 0.50 | 0.02 | -0.60 |
| **Glycocholic acid** | 0.03 | -0.70 | 0.04 | 0.52 | 0.37 | 0.16 | 0.60 | 0.56 | 0.68 | 0.57 | 0.88 | 0.53 | 0.21 | 0.64 | 0.78 | 0.47 | 0.11 | -0.48 | 0.03 | 0.75 |
| **Arachidonic acid** | 0.00 | 0.49 | 0.03 | -0.31 | 0.39 | -0.16 | 0.14 | -0.49 | 0.06 | -0.41 | 0.09 | -0.43 | 0.03 | -0.54 | 0.19 | -0.34 | 0.01 | 0.56 | 0.13 | -0.31 |
| **12(S)-HETE** | 0.00 | 0.60 | 0.01 | -0.46 | 0.31 | -0.18 | 0.19 | -0.48 | 0.12 | -0.52 | 0.23 | -0.49 | 0.03 | -0.59 | 0.37 | -0.43 | 0.04 | 0.50 | 0.04 | -0.67 |
| **15(S)-HpETE** | 0.00 | 0.67 | 0.01 | -0.58 | 0.25 | -0.22 | 0.26 | -0.46 | 0.23 | -0.45 | 0.35 | -0.59 | 0.05 | -0.59 | 0.50 | -0.54 | 0.02 | 0.47 | 0.03 | -0.69 |
| **20-Hydroxy-(5Z,8Z,11Z,14Z)-eicosatetraenoic acid** | 0.00 | 0.55 | 0.03 | -0.42 | 0.31 | -0.12 | 0.27 | -0.32 | 0.14 | -0.43 | 0.26 | -0.40 | 0.04 | -0.54 | 0.43 | -0.41 | 0.02 | 0.38 | 0.04 | -0.65 |
| **8(S)-Hydroxy-(5Z,9E,11Z,14Z)-eicosatetraenoic acid** | 0.00 | 0.64 | 0.03 | -0.45 | 0.29 | -0.25 | 0.21 | -0.43 | 0.12 | -0.52 | 0.20 | -0.49 | 0.03 | -0.57 | 0.34 | -0.49 | 0.00 | 0.45 | 0.05 | -0.67 |
